# Pathogen-induced biosynthetic pathways encode defense-related molecules in bread wheat

**DOI:** 10.1073/pnas.2123299119

**Published:** 2022-04-11

**Authors:** Guy Polturak, Martin Dippe, Michael J. Stephenson, Rajesh Chandra Misra, Charlotte Owen, Ricardo H. Ramirez-Gonzalez, John F. Haidoulis, Henk-Jan Schoonbeek, Laetitia Chartrain, Philippa Borrill, David R. Nelson, James K.M. Brown, Paul Nicholson, Cristobal Uauy, Anne Osbourn

**Affiliations:** ^a^Department of Biochemistry and Metabolism, John Innes Centre, Norwich NR4 7UH, United Kingdom;; ^b^Department of Crop Genetics, John Innes Centre, Norwich NR4 7UH, United Kingdom;; ^c^Department of Microbiology, Immunology and Biochemistry, University of Tennessee Health Science Center, Memphis, TN 38163

**Keywords:** natural products, phytoalexins, wheat, biosynthetic gene clusters, terpenes

## Abstract

Wheat is a widely grown food crop that suffers major yield losses due to attack by pests and pathogens. A better understanding of biotic stress responses in wheat is thus of major importance. The recently assembled bread wheat genome coupled with extensive transcriptomic resources provides unprecedented new opportunities to investigate responses to pathogen challenge. Here, we analyze gene coexpression networks to identify modules showing consistent induction in response to pathogen exposure. Within the top pathogen-induced modules, we identify multiple clusters of physically adjacent genes that correspond to six pathogen-induced biosynthetic pathways that share a common regulatory network. Functional analysis reveals that these pathways, all of which are encoded by biosynthetic gene clusters, produce various different classes of compounds—namely, flavonoids, diterpenes, and triterpenes, including the defense-related compound ellarinacin. Through comparative genomics, we also identify associations with the known rice phytoalexins momilactones, as well as with a defense-related gene cluster in the grass model plant *Brachypodium distachyon*. Our results significantly advance the understanding of chemical defenses in wheat and open up avenues for enhancing disease resistance in this agriculturally important crop. They also exemplify the power of transcriptional networks to discover the biosynthesis of chemical defenses in plants with large, complex genomes.

## Significance

Wheat is a globally important food crop that suffers major yield losses due to outbreaks of severe disease. A better mechanistic understanding of how wheat responds to pathogen attack could identify new strategies for enhancing disease resistance. Here, we discover six pathogen-induced biosynthetic pathways that share a common regulatory network and form part of an orchestrated defense response. Investigation of the wheat genome reveals that these pathways are each encoded by biosynthetic gene clusters (BGCs). We further show that these BGCs produce flavonoids and terpenes that may serve as phytoalexins or defense-related signaling molecules. Our results provide key insights into the molecular basis of biotic stress responses in wheat and open potential avenues for crop improvement.

The allohexaploid bread wheat (*Triticum aestivum* L.) accounts for ∼20% of the calories consumed by humans worldwide (1). Around one-fifth of the global annual wheat yield is lost due to pest and pathogen attack (2), a value that is expected to sharply rise as the climate warms (3, 4). A better understanding of how wheat responds to biotic stresses could enable the development of strategies for minimizing yield losses and reducing reliance on pesticides. Significant advances have been made in identification of wheat resistance genes (R genes) involved in pathogen recognition and the immune response (5). However, very little is known about the chemical defenses produced by wheat. The occurrence of the antimicrobial peptides defensins (6) and the constitutively produced defense compounds benzoxazinoids in wheat (7) has been known for decades. However, wheat phytoalexins (i.e., pathogen-induced small molecules) have only recently first been reported, with discovery of a variety of phenylamides that accumulate in wheat leaves inoculated with fungal pathogens (8).

The agronomic importance of wheat has led to extensive research into its genetics, and to the generation of a vast body of transcriptomic data from numerous studies into wheat development, physiology, and interactions with the environment. However, the first bread wheat genome assembly became available only recently because of the challenges associated with its large genome size, high repetitive sequence content, and relatedness between homologous subgenomes (namely the A, B, and D genomes) (9). The co-occurrence of these three subgenomes in modern bread wheat (AABBDD) is the result of a natural hybridization event(s) between the wild Tausch’s goatgrass (*Aegilops tauschii*; donor of the D genome) and domesticated emmer wheat (*Triticum turgidum* L.; donor of the A and B genomes) (10). The availability of the assembled wheat genome, together with vast transcriptomic resources, now offers the opportunity to employ a genomics-driven approach to uncover novel chemical defense molecules and biosynthetic pathways in this valuable crop. Such an approach is particularly useful for uncovering metabolites that are produced in small quantities or under specific conditions (e.g., pathogen-induced), thereby eluding traditional chemical analyses (11).

Here, by coupling gene coexpression network analysis with genome mining, we identify six defense-related candidate biosynthetic gene clusters (BGCs) in bread wheat. We show by expression of cluster genes in *Nicotiana benthamiana* that these BGCs encode pathways for the production of flavonoid, diterpene, and triterpene compounds that likely serve as broad-spectrum phytoalexins in wheat. Through comparative genomics, we also identify associations with phytoalexin clusters in other cereals and grasses. We further report the full characterization of the pathways for the defense compounds ellarinacin and brachynacin, which are, respectively, produced by related gene clusters in wheat and the grass purple false brome (*Brachypodium distachyon*). Our work uncovers biosynthetic pathways for pathogen-induced compounds in wheat and demonstrates a powerful approach for rapid discovery of defense-related molecules and metabolic pathways in crop plants, which may have future applications in crop protection.

## Results

### Gene Coexpression Network Analysis Coupled with Genome Analysis Identifies Candidate Pathogen-Induced Biosynthetic Gene Clusters in Wheat.

In a recently published study, 850 transcriptome datasets were compiled and analyzed to produce a genome-wide view of homolog expression patterns in hexaploid bread wheat. Weighted gene coexpression network analysis (WGCNA) was carried out based on gene expression patterns in the compiled datasets, and an additional set of networks was built for six separate subsample sets: grain, leaf, spike, root, abiotic, and disease (12).

**Fig. 1.**
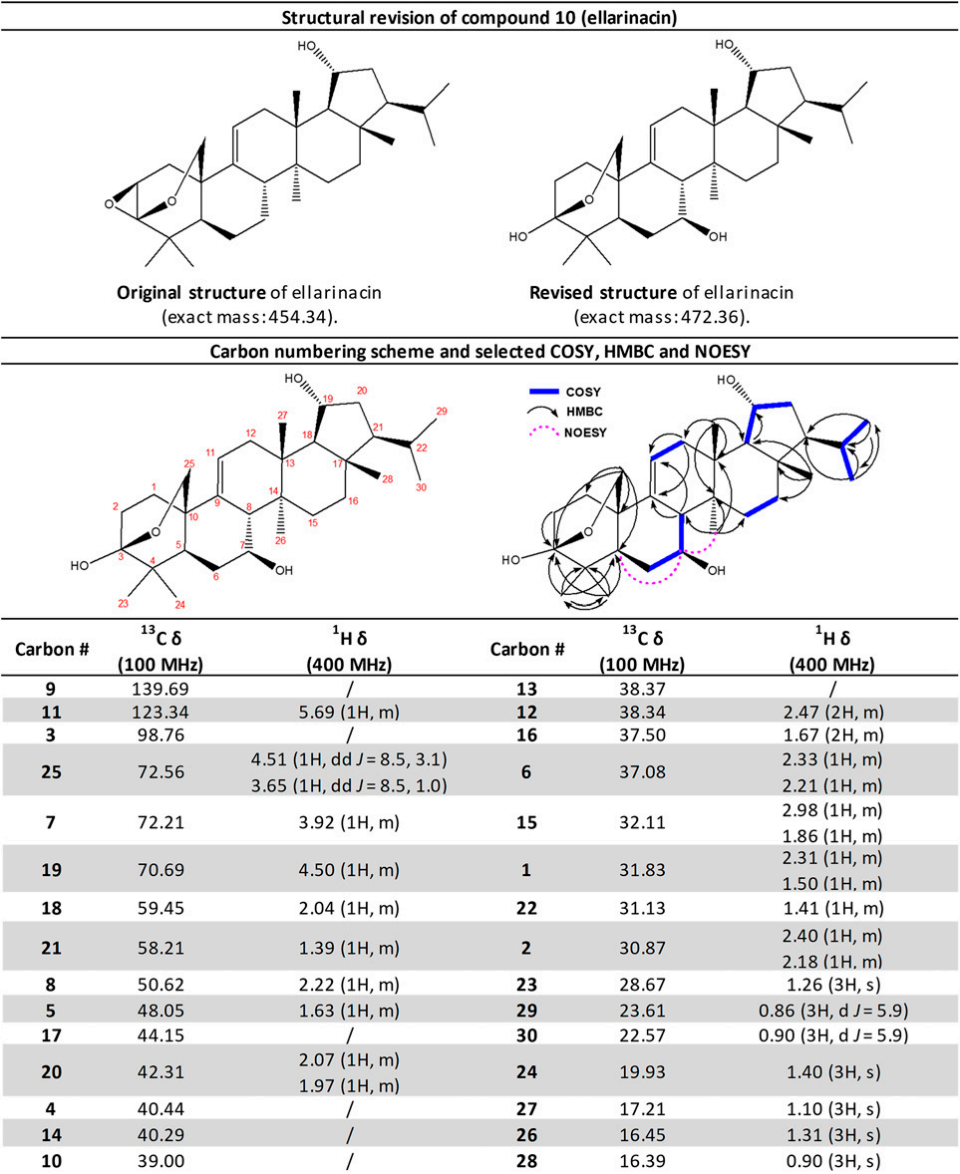
Top: The original and revised structure of compound **10** (**ellarinacin**). Bottom: The revised ^13^C & ^1^H δ assignments for **ellarinacin. Pyridine-d5** [referenced to residual solvent peak (^1^H δ: 8.74) (^13^C δ: 150.3)]. Assignments were made via a combination of ^1^H, ^13^C, DEPT-edited HSQC, HMBC, COSY and 2D NOESY experiments. Where signals overlap ^1^H δ is reported as the centre of the respective HSQC crosspeak. C7-OH was assigned as beta due to NOEs observed between C7-H and C5-H, and between C7-H and C26-H3, via a 2D NOESY experiment.

We hypothesized that new defense-related metabolites and metabolic pathways in wheat could be found by mining the “disease” gene network. Specifically, genes that are physically clustered in the genome and are coinduced by pathogens or pathogen-associated molecules could serve as excellent candidates for biosynthesis of defense compounds (11). WGCNA assigned 55,646 genes from the disease network (generated from 163 RNA-sequencing [RNA-seq] samples) into 69 modules based on their expression, and expression values of all genes in each module were averaged to get a single “eigengene” expression pattern per module (12). To find genes that exhibit a general, nonspecific induction by exposure to pathogens or pathogen-associated molecular patterns (PAMPs), we averaged for each module the difference in normalized eigengene expression between treatment and control in seven different studies and sorted the modules by the average expression delta (Fig. 1*A*). The top five modules (i.e., the modules represented by the most highly induced eigengenes), namely ME34, ME25, ME12, ME36, and ME8, showed consistent induction in all seven experiments used in the analysis (Fig. 1*B*) and were selected for further investigation.

To determine whether any of these gene expression modules contained genes that form putative biosynthetic gene clusters, we next mined the five modules by filtering for groups of three or more genes with successive accession numbers, that is, that are physically adjacent in the genome. A total of 55 groups were found (Dataset S1), which include groups of tandem duplicates, as expected. Twenty contain protein kinase genes with possible roles in biotic stress responses. A further six consist of genes for different types of enzyme families associated with plant-specialized metabolism, and so were identified as possible BGCs for synthesis of defense compounds. These six putative BGCs include two pairs of homologous clusters and were thus defined as four cluster types (1 to 4), and assigned as clusters 1(2A), 1(2D), 2(2B), 3(5A), 3(5D), and 4(5D). The bracketed numbers refer to the chromosomes that the clusters are located on (Fig. 1*C* and *SI Appendix*, Table S1). The two homologous cluster pairs are 1(2A) and 1(2D), and 3(5A) and 3(5D), respectively.

The majority of genes in the six putative BGCs were found in a single module, ME25, indicating highly similar expression patterns and suggesting possible coregulation of the BGCs by a shared network of transcription factors (TFs) (*SI Appendix*, Table S1). Analysis of a previously generated GENIE3-based wheat regulatory network (12) indeed revealed a highly overlapping network of TFs predicted to interact with the six BGCs. Specifically, 137 TFs predicted to interact with genes from two or more of the BGCs were found, including 21 TFs from 10 groups (i.e., groups of homoeologs or tandem duplicates) predicted to interact with genes from all six clusters. The top five most highly interacting TF groups included TFs from the WRKY, bHLH (two groups), NAC, and HSF families (Fig. 1*D* and Dataset S2), all of which have been associated with regulation of phytoalexin biosynthesis or pathogen resistance in plants (13, 14). Examination of Gene Ontology (GO) term enrichment of the predicted target genes of representative TFs from each of the five groups showed that the most significantly enriched terms are related to immune response or defense from biotic stress, for all five TFs excluding the NAC TF, for which the most significantly enriched GO terms were related to response to chemicals/toxins (Dataset S2). Of the 21 TFs that are associated with all six BGCs, none interact with any of the characterized genes for the biosynthetic pathway of the benzoxazinoids (e.g., DIBOA, DIMBOA), a group of well-characterized defense compounds found in several cereal crops, including wheat (15, 16) (Dataset S2). This is consistent with the definition of benzoxazinoids as phytoanticipins (constitutively produced defense compounds) (17), also reflected by the fact that the benzoxazinoid biosynthetic genes are not found in the pathogen-induced WGCNA expression modules. Interestingly, in contrast to maize, where benzoxazinoid biosynthesis is largely mediated by a BGC (18), the benzoxazinoid pathway in wheat is mostly dispersed (19). Only minimal clustering occurs in this pathway in wheat, in which the tryptophan synthase alpha subunit (*TaBX1*) and the cytochrome P450 (*TaBX2*) genes are paired on group 4 chromosomes, in addition to the tandem duplication of benzoxazinoid-related genes occurring in several loci (*SI Appendix*, Table S2).

### The Six Predicted Biosynthetic Gene Clusters Comprise Coexpressed Genes Potentially Involved in Diterpene, Triterpene, and Flavonoid Metabolism.

Plant BGCs typically contain one or more genes required for generation of a natural product scaffold, along with genes encoding downstream tailoring enzymes that modify this scaffold (e.g., cytochrome P450s [CYPs], sugar transferases [UGTs], methyltransferases [MTs]) (20). The six predicted pathogen-induced wheat BGCs each contain five to seven coexpressed biosynthetic genes (Fig. 1*C* and *SI Appendix*, Fig. S1). Based on the gene annotations, the predicted scaffold-forming enzymes for the clusters are terpene synthases (TPSs) [clusters 1(2A), 1(2D), and 2(2B)], oxidosqualene cyclases (OSCs) [clusters 3(5A) and 3(5D)], and a chalcone synthase (CHS) [cluster 4(5D)], hallmarks of diterpene, triterpene, and flavonoid biosynthesis, respectively. Notably, all three classes of compounds are associated with plant defense, including in the grasses (21–24).

Coexpression within each cluster was assessed by calculation of the Pearson correlation coefficient (*r* value) between the expression of a representative scaffold-forming gene from each cluster and other cluster genes, within an RNA-seq dataset including 68 experiments from the Wheat Expression Browser (http://www.wheat-expression.com) (12, 25). In the putative diterpene clusters 1(2A) and 1(2D), several genes were found to be highly coexpressed with the TPS bait (*r* > 0.8), including a copalyl diphosphate synthase (CPS), encoding a key enzyme in diterpene biosynthesis that typically catalyzes the preceding step to TPS; one 1(2D) or two 1(2A) UGTs; and three CYPs. In cluster 2(2B), two TPSs, two CYPs, and a CPS are coexpressed. In clusters 3(5A) and 3(5D) all five genes are coexpressed, while in cluster 4(5D) all genes are coexpressed with the exception of one CHS duplicate and a chalcone–flavanone isomerase (Fig. 1*C* and *SI Appendix*, Table S1).

### The Type 4 Biosynthetic Gene Cluster 4(5D) Encodes a Functional Flavonoid Biosynthetic Pathway.

To establish whether the predicted BGCs were likely to be functional, we first investigated the candidate flavonoid BGC 4(5D) (Fig. 1*C* and *SI Appendix*, Fig. S2 and Table S1). The genes for the predicted scaffold-generating enzyme (TaCHS1) and coexpressed tailoring enzymes (TaCYP71C164 and TaOMT3/6/8) were cloned and transiently expressed in *N. benthamiana* by agroinfiltration (26), together with the clustered chalcone–flavanone isomerase (chi-D1), and an additional CYP71 gene (TaCYP71F53_5D), which is located 425 kb upstream of the terminal O-methyltransferase (OMT) of the cluster and also belongs to the ME25 expression module. A fourth OMT in the cluster (TaOMT7) is a tandem duplicate of TaOMT6 with a single–amino acid difference and was not included in the analysis. The combined expression of all genes resulted in formation of a new product exhibiting ultraviolet (UV) absorbance (λ_max_ 260 nm) with exact mass [M+H = 329.1010] (*SI Appendix*, Fig. S2) and predicted elemental composition C_18_H_17_O_6_ (−1.78 ppm [parts per million]). This product was not produced in combinations in which TaCHS1 or any of the two CYPs and three OMTs were omitted, indicating that the proteins encoded by all six genes are enzymatically active (Fig. 1*E* and *SI Appendix*, Fig. S2). Inclusion of chi-D1 was not essential for formation of this product in *N. benthamiana* (*SI Appendix*, Fig. S2). Thus, the coexpressed genes within cluster 4(5D) encode a functional pathway that, based on UV absorbance, exact mass, and the calculated elemental composition of the putative end product, is likely to produce a hydroxy-trimethoxy-flavone. Future work is needed to fully elucidate the structures of the pathway end product and intermediates.

### The Homologous Type 1 Biosynthetic Gene Clusters 1(2A) and 1(2D) Are Related to but Functionally Distinct from the Rice Momilactone Cluster.

Rice produces a variety of diterpene phytoalexins for which the biosynthetic pathways are well-characterized. The genes for several of the pathways for labdane-related diterpenes (e.g., momilactones, phytocassanes/oryzalides) are clustered in the rice genome (27–29). These labdane-related diterpenes are formed from the universal diterpenoid precursor geranylgeranyl diphosphate (GGPP) (**1**) via initial cyclization reactions catalyzed by CPSs that produce normal, *ent*, or *syn* stereoisomers of copalyl diphosphate (CPP). The CPP intermediates are subsequently utilized by TPSs to form various diterpene backbones, which then typically undergo further tailoring reactions (30). In wheat, diterpene metabolism is considerably less well characterized than in rice. In previous studies aimed at functional characterization of diterpene-related genes in wheat, five CPSs (CPS1 to 5) and six kaurene synthase-like terpene TPSs (KSL1 to 6) were cloned and characterized by recombinant expression (31–33). Four of the CPS enzymes catalyzed production of normal or *ent* stereoisomers of CPP, while five of the KSL enzymes were shown to convert normal-, *ent*-, or *syn*-CPP to several different diterpene products. The physical location and general expression patterns of these genes were, however, unknown.

**Fig. 2.**
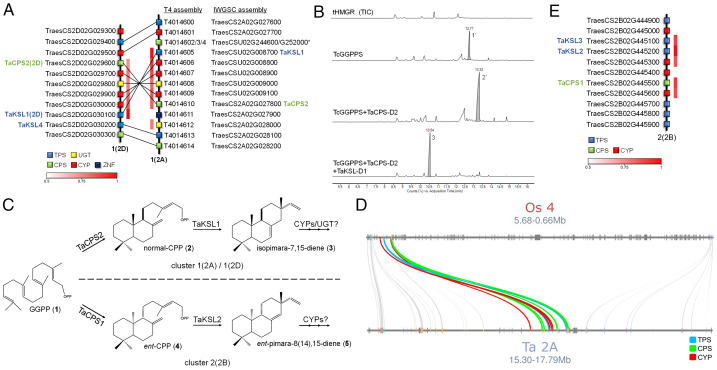
Diterpene-producing BGCs are found on group 2 chromosomes in bread wheat. (*A*) Assignment of homologous genes in the type 1 clusters 1(2A) and 1(2D), including the previously characterized genes *TaCPS2*, *TaKSL1*, and *TaKSL4*. Chr.2A genes were positioned based on the T4 wheat genome assembly and homoeologs were assigned based on pairwise sequence alignments. The T4 assembly reveals the presence of five Chr.2D homoeologs in inverted positions on Chr.2A (*TraesCSU02G008700-G009100*), which were previously unmapped in the IWGSC assembly. *CPS* genes *TraesCSU02G252000* and *TraesCSU02G244600* (asterisk) have partial coding sequences and are orthologous to the *OsCPS4* gene in the rice momilactone BGC. The white-to-red color coding denotes Pearson correlation (*r*) values for expression of each gene with a representative bait gene from the cluster. (*B*) GC-MS analysis of leaf extracts following expression of the wheat TaKSL-D1 and TaCPS-D2 enzymes in *N. benthamiana*. Cytosol-targeted TaKSL-D1 and TaCPS-D2 were transiently expressed together with a *Taxus canadensis* GGPP synthase and oat tHMGR. Total-ion chromatograms (TICs) are shown. Peaks were putatively identified as geranylgeraniol (**1′**), copalol (**2′**), and isopimara-7,15-diene (**3**), based on comparison of mass spectra with the National Institute of Standards and Technology (NIST) database and the literature (*SI Appendix*, Fig. S3). (*C*) Predicted pathways for diterpene production by the type 1 BGCs 1(2A) and 1(2D) and the type 2 BGC 2(2B). The type 1 clusters 1(2A) and 1(2D) comprise coexpressed genes for TaCPS2 and TaKSL1, CYPs, and UGTs, predicted to form isopimara-7,15-diene–derived diterpenoids from GGPP. Cluster 2(2B) includes coexpressed genes for TaCPS1, TaKSL2, TaKSL3, and two CYPs, putatively forming pimara-8(14),15-diene–derived diterpenoids from GGPP. (*D*) Microsynteny analysis of wheat BGC 1(2A) (T4 assembly) and the momilactone cluster in a syntenic region in rice Chr.4. (*E*) Structure of BGC 2(2B) and assignment of the previously characterized genes *TaCPS1*, *TaKSL2*, and *TaKSL3*.

Interestingly, our study identified two of these genes, namely *TaKSL1* and *TaCPS2*, as the coexpressed TPS and CPS genes in cluster 1(2A). A third gene, *TaKSL4*, is found in the homologous 1(2D) cluster (Fig. 2*A*). *TaKSL4* is not coexpressed with other cluster genes and generally exhibits a root-specific, noninduced expression pattern (*SI Appendix*, Fig. S1). The colocalization and coexpression of *TaKSL1* and *TaCPS2* coincide with their previously ascribed enzymatic functions—TaCPS2 produces normal-CPP (**2**), while TaKSL1 acts on normal-CPP to produce isopimara-7,15-diene (**3**) (31, 32). TaKSL1 can also react with a *syn*-CPP substrate, but a *syn*-CPP–producing copalyl synthase is yet to be identified in wheat (32). Transient expression of the chromosome 2D (Chr.2D) homoeologs of TaCPS2 and TaKSL1 (named TaCPS-D2 and TaKSL-D1 hereinafter) in *N. benthamiana* revealed that these enzymes are functional and produce compounds with mass spectra matching copalol and isopimara-7,15-diene, respectively, confirming the activity of this pair of genes in the 1(2D) cluster (Fig. 2*B* and *SI Appendix*, Fig. S3). The occurrence of additional coexpressed CYP genes and a UGT gene in the 1(2D) and 1(2A) clusters (Fig. 2*A*) suggests that these clusters form pathogen-induced pathways for production of isopimara-7,15-diene–derived diterpenes (Fig. 2*C*).

Intriguingly, microsynteny analysis between wheat and rice suggests that the type 1 clusters present on wheat chromosomes 2A and 2D [BGCs 1(2A) and 1(2D)] likely share a common evolutionary origin with the rice momilactone cluster. The *KSL* genes in clusters 1(2A) and 1(2D) are close homolog of the *OsKSL4* gene from the rice momilactone BGC (27, 28). Directly adjacent to *TaKSL*1 is a *CPS* gene that is orthologous to *OsCPS4*. The wheat cluster also includes four cytochrome P450s belonging to the CYP99 family that are homologs of the CYP99A2/A3 P450 pair in the rice momilactone BGC (28). Furthermore, the chromosomal regions harboring wheat clusters 1(2A) and 1(2D) are syntenic to the region of the rice genome containing the momilactone cluster, which is found on rice Chr.4, the corresponding chromosome of wheat Chr.2 (34) (Fig. 2*D*). However, although these clusters may share a common evolutionary origin, they produce different types of diterpenes: the rice momilactones are derivatives of the *syn*-CPP–derived scaffold *syn*-pimara-7,15-diene (27), while functional characterization and gene expression data of the wheat 1(2D) cluster and previous characterization of the *TaKSL1* and *TaCPS2* genes (31, 32), which we have shown to be in wheat cluster 1(2A), imply that these two BGCs encode pathways that yield derivatives of the normal-CPP–derived isopimara-7,15-diene scaffold. Of note, the rice momilactone cluster also includes two short-chain dehydrogenase/reductase (SDR) genes, *OsMAS* and *OsMAS2* (28, 35), that do not have apparent orthologs in the wheat type 1 clusters or elsewhere in the wheat genome.

The third predicted diterpene BGC that we found, cluster 2(2B), also includes three other previously characterized wheat genes, namely *TaCPS1*, *TaKSL2*, and *TaKSL3* (31, 32), all of which are coexpressed (Fig. 2*E*). TaCPS1 catalyzes formation of *ent*-CPP (**4**), while TaKSL2 acts on *ent*-CPP to produce pimara-8(14),15-diene (**5**). TaKSL3, a tandem duplicate of TaKSL2, only exhibits low activity, selectively acting on *ent*-CPP to produce two unknown products (32). The combined functions of TaCPS1 and TaKSL2, together with the presence of additional coexpressed CYPs in the cluster, suggest that BGC 2(2B) encodes a pathway for production of *ent*-pimara-8(14),15-diene derivatives (Fig. 2*C*).

### The Homologous Type 3 Cluster 3(5D) Encodes a Biosynthetic Pathway to Ellarinacin, an Arborinane-Type Triterpenoid.

**Fig. 3.**
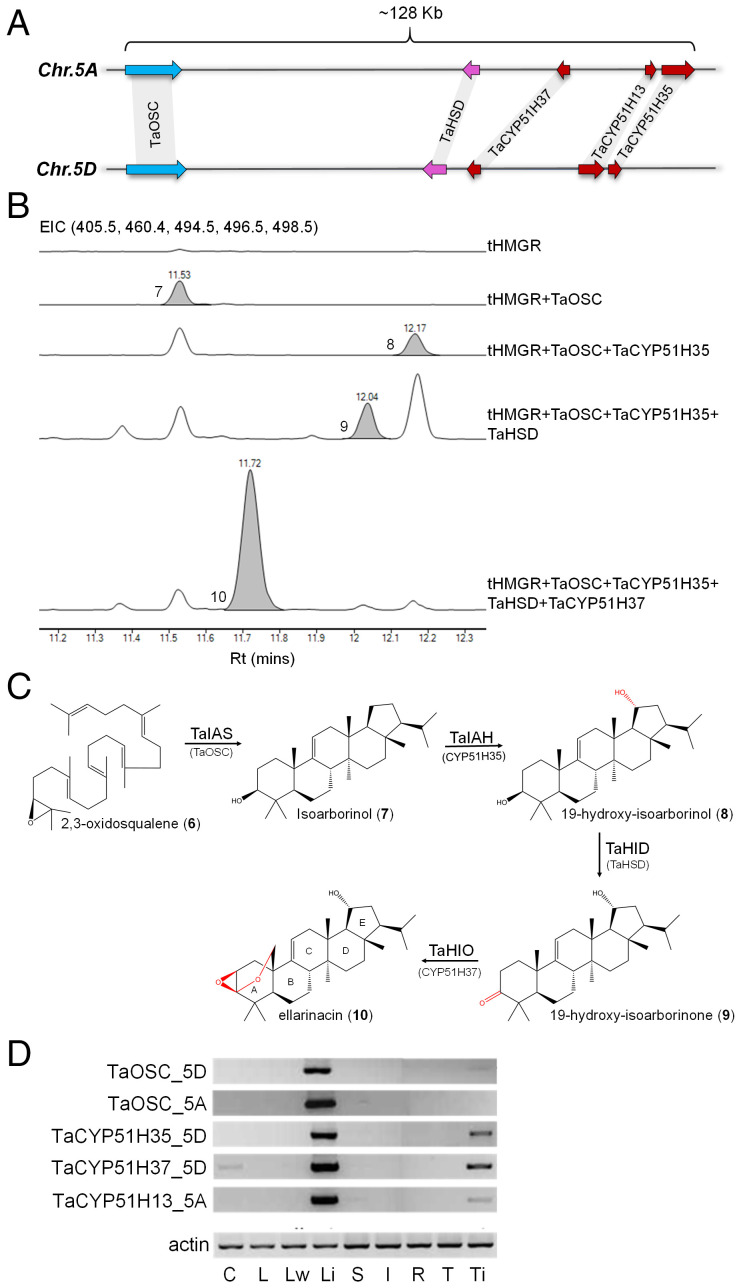
Wheat cluster 3(5D) produces an isoarborinol-derived triterpenoid. (*A*) Structures of homologous triterpene biosynthetic gene clusters identified on wheat chromosomes 5A and 5D. (*B*) GC-MS traces for wheat BGC 3(5D) genes transiently expressed in *N. benthamiana*. EIC, extracted-ion chromatogram for ions representing isoarborinol (**7**) (498.5), 19-hydroxy-isoarborinol (**8**) (496.5), 19-hydroxy-isoarborinone (**9**) (494.5), ellarinacin (**10**) (405.5), and internal standard 5α-cholestan-3β-ol (460.4). (*C*) Assigned structure of ellarinacin and predicted biosynthetic pathway in wheat. TaIAH, isoarborinol 19-hydroxylase; TaIAS, isoarborinol synthase; TaHID, 19-hydroxy-isoarborinol dehydrogenase; TaHIO, 19-hydroxy-isoarborinone oxidase. Rings A through E are annotated. (*D*) Semiquantitative RT-PCR of selected genes from type 3 clusters 3(5A) and 3(5D) in Chinese Spring wheat tissues. C, coleoptile; I, inflorescence; L, leaf; Li, leaf infected with *B. graminis* f. sp. *tritici*; Lw, leaf after wounding; R, root; S, stem; T, root tip; Ti, root tip after infection with *G. graminis*.

The type 3 cluster 3(5D) contains genes implicated in triterpenoid biosynthesis, most notably a predicted oxidosqualene cyclase gene (*TaOSC*). Flanking *TaOSC* are three cytochrome P450s (*TaCYP51H35*, *TaCYP51H37*, and *TaCYP51H13P*) and a gene annotated as a 3β-hydroxysteroid-dehydrogenase/decarboxylase (*TaHSD*) (Fig. 3*A*). The genomic sequences of *TaOSC*, *TaHSD*, *TaCYP51H35*, and *TaCYP51H37* predict full coding sequences for all four genes, while *TaCYP51H13P* was found by manual annotation to carry two premature stop codons (*SI Appendix*, Fig. S4) and was designated a pseudogene. The homologous cluster on Chr.5A is similarly structured (Fig. 3*A*), but with a predicted full coding sequence for *TaCYP51H13_5A*. Amino acid sequence identity between homologous pairs in the 3(5D) and 3(5A) clusters is >99% for TaOSC, TaHSD, and TaCYP51H37 and >97% for TaCYP51H35 and TaCYP51H13. As for the type 1 diterpene cluster, homoeologs of the type 3 cluster genes are not found in the B genome. However, a homologous gene cluster is present adjacent to the 3(5D) cluster on Chr.5D, which includes paralogs of the *TaOSC*, *TaHSD*, and *TaCYP51H* genes. Similarly, one *TaOSC* and two *CYP51H* paralogs are also found on Chr.5A, adjacent to the 3(5A) cluster (*SI Appendix*, Fig. S5). These Chr.5A and Chr.5D paralogs, however, in general have low expression across all transcriptomic data available at www.wheat-expression.com, and so are not likely to belong to active BGCs (*SI Appendix*, Table S3).

Functional analysis of the cluster 3(5D) genes was carried out by transient expression in *N. benthamiana*. All genes were coinfiltrated with an *Agrobacterium* strain harboring an expression construct for a feedback-insensitive form of 3-hydroxy-3-methylglutaryl coenzyme A reductase (tHMGR) from oat, which enhances triterpenoid precursor supply (36). Gas chromatography–mass spectrometry (GC-MS) and liquid chromatography–mass spectrometry (LC-MS) analyses of leaf extracts revealed that the four enzymes TaOSC, TaHSD, TaCYP51H35, and TaCYP51H37 form a sequential biosynthetic pathway (Fig. 3*B* and *SI Appendix*, Figs. S6–S9). As the *TaCYP51H13P* pseudogene from cluster 3(5D) does not encode a complete functional protein, we tested the activity of its Chr.5A homolog, *TaCYP51H13_5A*, through agroinfiltration with the four 3(5D) cluster genes in different combinations. TaCYP51H13_5A exhibited the same activity as TaCYP51H35, but to a lower extent, resulting in lower levels of product compared with TaCYP51H35 (*SI Appendix*, Fig. S10). This redundant activity provides a possible explanation why *TaCYP51H13* is not conserved in the Chr.5D cluster.

The structures of the purified products of coexpression of TaOSC + TaCYP51H35, and of the combined four cluster genes [all from cluster 3(5D)], were determined by NMR following large-scale vacuum-mediated agroinfiltration and purification (*SI Appendix*, Figs. S11 and S12 and Tables S4 and S5). The product of coexpression of TaOSC + TaCYP51H35 was identified as 19-hydroxy-isoarborinol (**8**), indicating that TaOSC (hereinafter isoarborinol synthase; TaIAS) synthesizes the triterpene scaffold isoarborinol (**7**) which is subsequently hydroxylated by TaCYP51H35 (hereinafter isoarborinol 19-hydroxylase; TaIAH). The product of coexpression of all four cluster genes was found to have an unusual triterpenoid structure, with a β-epoxy group and an ether bridge attached to the A ring (Fig. 3*C*). The GC/LC-MS data and NMR-assigned structure together suggest oxidation of the 3-alcohol to the ketone 19-hydroxy-isoarborinone (**9**) by TaHSD (hereinafter 19-hydroxy-isoarborinol dehydrogenase; TaHID); TaCYP51H37 (hereinafter 19-hydroxy-isoarborinone oxidase; TaHIO) likely then hydroxylates the C25-methyl carbon, leading to nucleophilic attack of the A ring ketone, thus forming a hemiacetal intermediate which further reacts to produce the epoxide. This unusual reaction may involve two independent catalytical cycles mediated by TaHIO. This would be in line with the only other previously reported noncanonical CYP51 enzyme (AsCYP51H10, *Sad2*) which hydroxylates the C16 position of the β-amyrin scaffold and also converts an alkene to an epoxide at C12–C13 via two independent reactions (37). However, a mechanism involving just one catalytic cycle may also be possible (*SI Appendix*, Fig. S13). The structure of the BGC 3(5D) product was named ellarinacin (**10**). The proposed biosynthetic pathway is shown in Fig. 3*C*.

Interestingly, the ellarinacin cluster [BGC 3(5D)] provides multiple links to sterol metabolism. Production of plant sterols from 2,3-oxidosqualene (**6**) is initiated by highly conserved OSCs known as cycloartenol synthases (CASs), while triterpene scaffolds are generated from 2,3-oxidosqualene by other diverse OSCs (triterpene synthases) (22). TaOSC shares higher sequence similarity with known monocot CAS enzymes than with any of the other triterpene synthases that have been functionally characterized from monocots to date (*SI Appendix*, Figs. S14 and S15). Plant 3β-hydroxysteroid-dehydrogenase/decarboxylases belong to the SDR superfamily and are involved in biosynthesis of phytosterols and steroidal glycoalkaloids (38–41). Phylogenetic analysis shows that TaHSD is related to *Arabidopsis thaliana* genes *3*β*HSD/D1* and *3*β*HSD/D2*, that take part in sterol biosynthesis (*SI Appendix*, Fig. S16) (38, 42). The cytochrome P450 genes found in the cluster provide further connections to sterol metabolism, as they belong to the sterol-related CYP51 family (*SI Appendix*, Figs. S17 and S18). CYP51 enzymes catalyze 14α‐demethylation of sterols in all eukaryotes and are the only family of cytochrome P450s that are evolutionarily conserved from prokaryotes through fungi, plants, and mammals (43). To date, only one plant CYP51 has been found to catalyze a reaction different from the canonical sterol demethylase activity—AsCYP51H10 (*Sad2*), which is involved in biosynthesis of an antifungal triterpene glycoside known as avenacin in oat (37, 44). Several members of the ellarinacin cluster thus appear to have been recruited from sterol biosynthetic genes, most likely through gene duplication and neofunctionalization.

### The Ellarinacin Cluster Is Highly Induced by Biotic Stress.

We next sought to determine whether the type 3 clusters are likely to be involved in plant defense by further investigating the expression patterns of the clustered genes. Analysis of the http://www.wheat-expression.com dataset revealed that the expression patterns of the genes were consistent with their positioning in the ME25 and ME34 modules, namely that they showed induction by various fungal pathogens and by the PAMPs chitin and flg22 (*SI Appendix*, Fig. S19). Notably, the clusters were not substantially induced in response to various abiotic stresses, including drought, heat, cold, phosphate starvation, and drought-simulating treatment with polyethylene glycol 6000 (*SI Appendix*, Fig. S20).

**Fig. 4.**
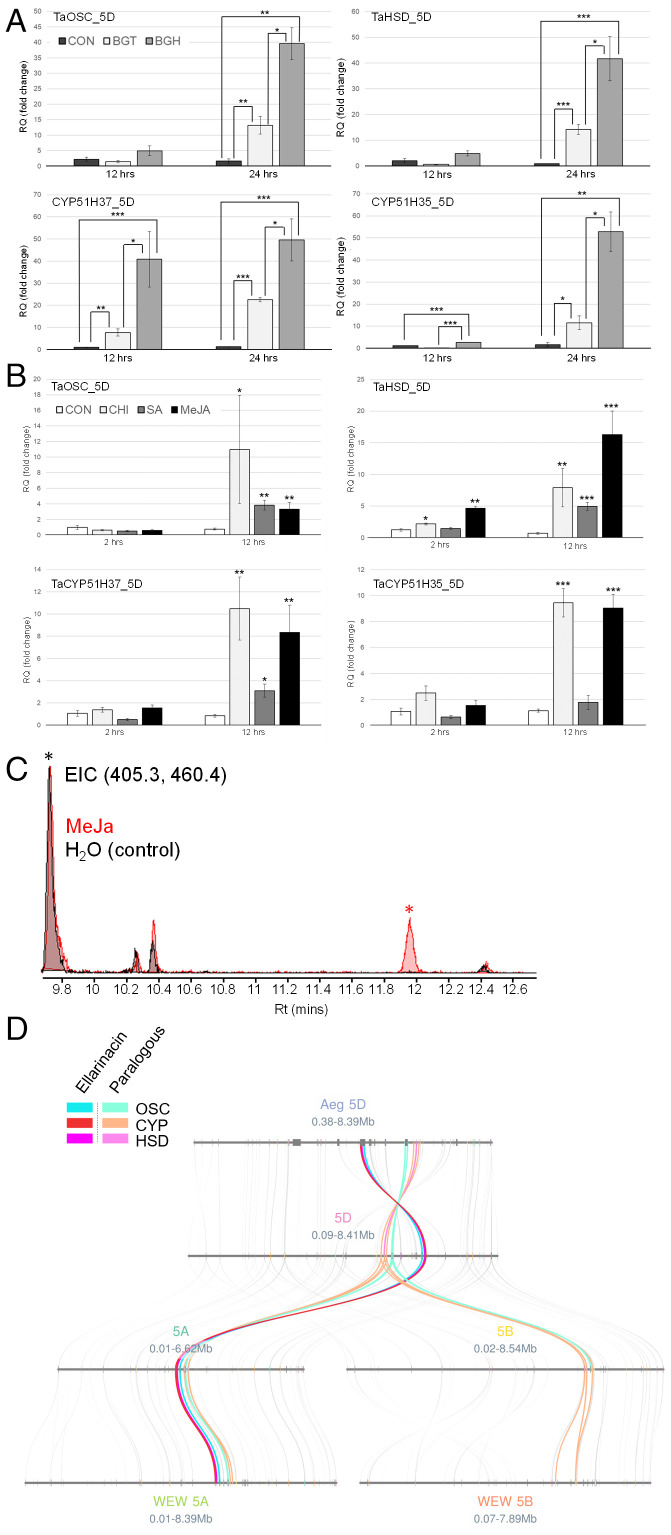
Ellarinacin BGC 3(5D) is induced by pathogens and elicitors. (*A*) Quantitative real-time PCR of ellarinacin BGC genes in detached wheat leaves infected with two powdery mildew isolates, 12 and 24 h post infection. BGT and BGH, infected with wheat-adapted isolate *B. graminis* f. sp. *tritici* or the nonadapted isolate *B. graminis* f. sp. *Hordei*, respectively; CON, control (noninfected). (*B*) Quantitative real-time PCR for ellarinacin BGC genes in detached wheat leaves treated with MeJa, SA, chitin (CHI), or H_2_O (CON), for 2 or 12 h. For *A* and *B*, relative quantification values (in fold change) indicate means of three biological replicates ± SEM. Asterisks denote *t* test statistical significance of differential expression. *P < 0.05, **P < 0.01, ***P < 0.001. (*C*) GC-MS analysis of TMS-derivatized extracts from wheat leaves treated with MeJa or H_2_O (control) for 3 d. EICs are for ions representing ellarinacin (405.3, Rt 11.94, red asterisk) and 5α-cholestan-3β-ol (460.4, Rt 9.70, black asterisk). (*D*) Microsynteny analysis of the region surrounding the ellarinacin BGC and its paralogous cluster on Chr.5 of the wheat A, B, and D genomes, and wheat progenitors *A. tauschii* (Aeg) and wild emmer wheat (WEW).

The observed expression pattern of the type 3 clusters was further supported by semiquantitative reverse-transcription PCR (RT-PCR) analysis of selected genes from BGCs 3(5A) and 3(5D) using homolog-specific primers: Expression of all tested genes was strongly induced in leaves infected with powdery mildew but not by mechanical wounding, with little or no expression in the other various wheat tissues analyzed (Fig. 3*D*). Weak induction was also observed in roots infected with *Gaeumannomyces graminis*, a soil-borne fungus that causes “take-all” disease. Induction of the entire BGC 3(5D) by infection with powdery mildew was further validated by quantitative real-time PCR. Detached wheat leaves were exposed to spores of either wheat-adapted (*Blumeria graminis* f. sp. *tritici*; Bgt) or nonadapted (*Blumeria graminis* f. sp. *hordei*; Bgh) isolates of powdery mildew, and relative transcript abundance was determined 12 and 24 h post infection. Treatment with Bgt or Bgh resulted, in both cases, in strong induction of the four cluster genes. Interestingly, induction was more marked for Bgh (nonadapted) compared with Bgt (Fig. 4*A*). Our analyses of transcriptome data from previously published studies (45, 46) in which wheat plants were challenged with the fungal pathogens powdery mildew, cereal blast (*Magnaporthe* spp.), and leaf or yellow rust (*Puccinia* spp.) also revealed stronger induction of the cluster genes by nonhost vs. host interactions (*SI Appendix*, Figs. S21 and S22).

Finally, we analyzed gene expression in detached wheat leaves treated with the elicitors methyl jasmonate (MeJa) and salicylic acid (SA), as well as with the PAMP chitin. All four cluster 3(5D) genes analyzed (*TaOSC*, *TaHSD*, *TaCYP51H35*, *TaCYP51H37*) were significantly induced compared with the control 12 h after treatment with MeJa, SA, or chitin, with the exception of *TaCYP51H35* in SA-treated leaves (Fig. 4*B*). Thus, the ellarinacin cluster is highly induced by biotic stress, suggesting a possible function in wheat response against pathogens. The very low basal expression in various wheat tissues, as observed in the RT-PCR and RNA-seq data analysis, and strong induction by pathogens, defense-related hormones, and PAMPs further suggest that ellarinacin serves as a phytoalexin rather than a phytoanticipin. Correspondingly, GC-MS analysis detected ellarinacin in extracts of MeJa-treated but not control detached wheat leaves (Fig. 4*C*). A 60% increase in isoarborinol levels was also observed in MeJa-treated leaves compared with control leaves (*SI Appendix*, Fig. S23).

### The Ellarinacin Cluster Is Conserved in Wheat Ancestors.

**Fig. 5.**
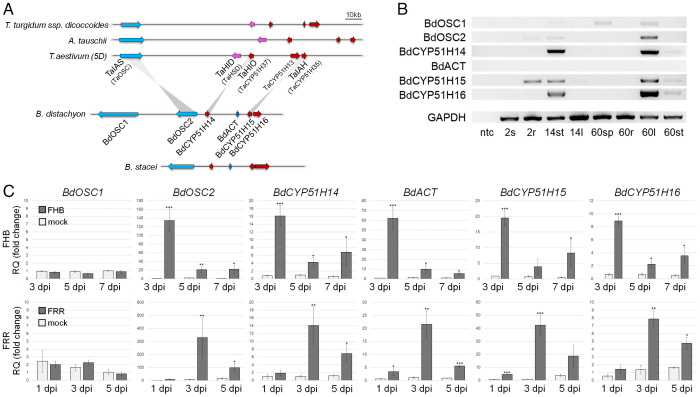
Occurrence and expression of ellarinacin-like BGCs in *Brachypodium* and wheat ancestral species. (*A*) Wheat ellarinacin BGC is conserved in wheat wild ancestors *A. tauschii* and wild emmer wheat (*T. turgidum* subsp. *dicoccoides*), and homologous to a BGC identified on chromosome 3 of *B. distachyon*. Gray lines link between wheat and *B. distachyon* BlastP reciprocal best hits. (*B*) Semiquantitative RT-PCR of *B. distachyon* Chr.3 clustered genes. ntc, no template control; 2s, seedling shoot (2 d old); 2r, seedling root; 14st, young plant stem base (14 d old); 14l, young plant leaf; 60sp, mature plant spike (60 d old); 60r, mature plant root; 60l, mature plant leaf; 60st, mature plant stem base. (*C*) Quantitative real-time PCR of brachynacin BGC genes in *B. distachyon* plants infected with FHB or FRR. Con, control (noninfected); dpi, days post infection. Relative quantification values (in fold change) indicate means of three biological replicates ± SEM. Asterisks denote *t* test statistical significance of differential expression. *P < 0.05, **P < 0.01, ***P < 0.001.

We next investigated whether ellarinacin-like clusters also exist in the genomes of ancestral species of common wheat. Specifically, we looked for related clusters in two wild progenitors that have sequenced genomes: *A. tauschii* (Tausch’s goatgrass; donor of the D genome of bread wheat) and *Triticum turgidum* subsp. *dicoccoides* (wild emmer wheat, progenitor of cultivated emmer; the donor of the A and B genomes of bread wheat). Microsynteny analysis of the regions surrounding the homologous type 3 BGCs on Chr.5A and Chr.5D shows that while these clusters appear to be conserved on chromosome 5 of the A and D genomes of *A. tauschii* and wild emmer wheat, a homologous cluster could not be found on chromosome 5B of bread wheat or wild emmer wheat. Chromosome 5B of both species do, however, contain homoeologs of the OSC and/or P450s of the paralogous, transcriptionally nonactive cluster in the A and D genomes (Fig. 4*D*). The wild emmer wheat and *A. tauschii* clusters each contain an OSC, an HSD, and three CYP51 genes, in the same order and orientation as in wheat (Fig. 5*A*). Sequence comparison of the cluster genes in wheat and its two wild progenitors revealed that the predicted protein sequences are also highly conserved (>99.4% amino acid identity for all proteins in both species; *SI Appendix*, Table S6). To assess the functionality of the *A. tauschii* cluster, we transiently expressed the first two genes of the predicted *A. tauschii* pathway, namely the orthologs of *TaIAS* and *TaIAH*, in *N. benthamiana*. Coexpression of the two genes resulted in formation of 19-hydroxy-isoarborinol (*SI Appendix*, Fig. S24), the same product obtained by *TaIAS* and *TaIAH* expression. The coding sequence of the *A. tauschii* ortholog of *TaCYP51H13P* contains one of the premature stop codons found in its wheat homolog (*SI Appendix*, Fig. S4), and so is likely to be nonfunctional. The remaining predicted active enzymes in the *A. tauschii* pathway, orthologs of TaHID and TaHIO, exhibit 100% amino acid identity with their wheat counterparts, suggesting that the *A. tauschii* cluster encodes a complete biosynthetic pathway for ellarinacin.

### Arborinane-Type Clusters Are Found in Other Grasses.

The occurrence of conserved ellarinacin-like clusters in wheat and its progenitors raised the possibility that BGCs for ellarinacin or other arborinane-type terpenoids may also occur in other grasses. The isoarborinol scaffold has been reported from other Poaceae species, including sorghum (47) and rice (48). We therefore searched for orthologs of *TaIAS* in additional Poaceae species, based on sequence similarity. Orthologs for *TaIAS* could not be identified in maize, sorghum, barley, and rice. The latter has a previously characterized isoarborinol synthase gene (48), but this gene bears low similarity to *TaIAS* (56% similarity on the amino acid level) and has most likely evolved independently.

A BlastP search of TaIAS against the recently published genome of the diploid oat species *Avena strigosa* (49) found a candidate OSC gene on chromosome 1, herein named *AsOSC1*, with high predicted amino acid sequence similarity to the TaIAS protein (91.2%). This was also the reciprocal best hit (RBH) of TaIAS. Flanking *AsOSC1* (∼25 kb away) is a *CYP51H* gene, herein named *CYP51H73*. Transient expression of AsOSC1 in *N. benthamiana* yielded a new product, which was verified by GC-MS as isoarborinol. No additional products were detected when AsCYP51H73 was coexpressed with AsOSC1 (*SI Appendix*, Fig. S25). Since *AsCYP51H73* is orthologous with wheat *TaCYP51H37* (*TaHIO*), we also tested if AsCYP51H73 would exhibit the same or similar activity, by coexpressing AsCYP51H73 together with TaIAS, TaIAH, and TaHID. However, no activity was detected. Examination of transcriptomic data from six *A. strigosa* tissues (49) reveals that similar to the ellarinacin cluster in wheat, *AsOSC1* and *AsCYP51H73* exhibit near zero normalized expression values (reads per kilobase of transcript, per million mapped reads [RPKM]) in all analyzed tissues, including leaf, shoot, panicle, spikelet, root, and root tip (*SI Appendix*, Table S7). It remains to be seen, however, whether these genes are also similarly induced by pathogen infection.

In the genome of the grass model plant *B. distachyon* (strain *Bd21*) (50), a *TaIAS* homolog was identified on chromosome 3, *BdOSC2*, which was the RBH of *TaIAS*. Flanking this gene were genes predicted to encode another highly similar OSC (*BdOSC1*), and three cytochrome P450s of the CYP51H subfamily (*BdCYP51H14*, *BdCYP51H15*, and *BdCYP51H16*) (Fig. 5*A*). A predicted BAHD-type acyltransferase gene (*BdACT*) was also found between *BdCYP51H14* and *BdCYP51H15*. Thus, together, these genes form a potential BGC for production of arborinane-type or similar triterpenoids in *B. distachyon*. A conserved cluster that has a similar gene structure to the *B. distachyon* BGC but with one OSC gene only was also found in the genome of the closely related species *Brachypodium stacei* (Fig. 5*A*).

### The *B. distachyon* Chr.3 BGC Is Induced by Fungal Pathogens.

To test whether the clustered genes identified on chromosome 3 of *B. distachyon* might form an active BGC, their expression profiles were examined. Analysis of *B. distachyon* gene expression datasets in the Joint Genome Institute Gene Atlas (https://phytozome.jgi.doe.gov/) (51) and PlaNet (https://aranet.mpimp-golm.mpg.de/) (52, 53) showed that the three *CYP51* and two *OSC* genes are coexpressed, with highest expression in the mature leaf and stem base. The BAHD acyltransferase gene displayed a similar pattern, but with markedly lower overall expression values (*SI Appendix*, Fig. S26). Relative expression of all six genes was further assessed by semiquantitative RT-PCR of seven *B. distachyon* tissues at different developmental stages. The cluster genes generally exhibited highest expression in the leaves and stem base (Fig. 5*B*). A *BdACT* amplicon could only be detected with extended exposure (*SI Appendix*, Fig. S27). Unlike the other cluster genes, *BdOSC1* was also expressed in the spikes of mature plants.

qRT-PCR analysis of *B. distachyon* plants infected with *Fusarium graminearum* causing *Fusarium* head blight or *Fusarium* root rot showed that, as for the wheat cluster, the *B. distachyon* cluster is highly induced by fungal pathogens. Significant increases in gene expression following infection were observed in both experiments for all clustered genes except *BdOSC1* (Fig. 5*C*).

### The *B. distachyon* Chr.3 BGC Produces an Arborinane-Type Triterpenoid.

**Fig. 6.**
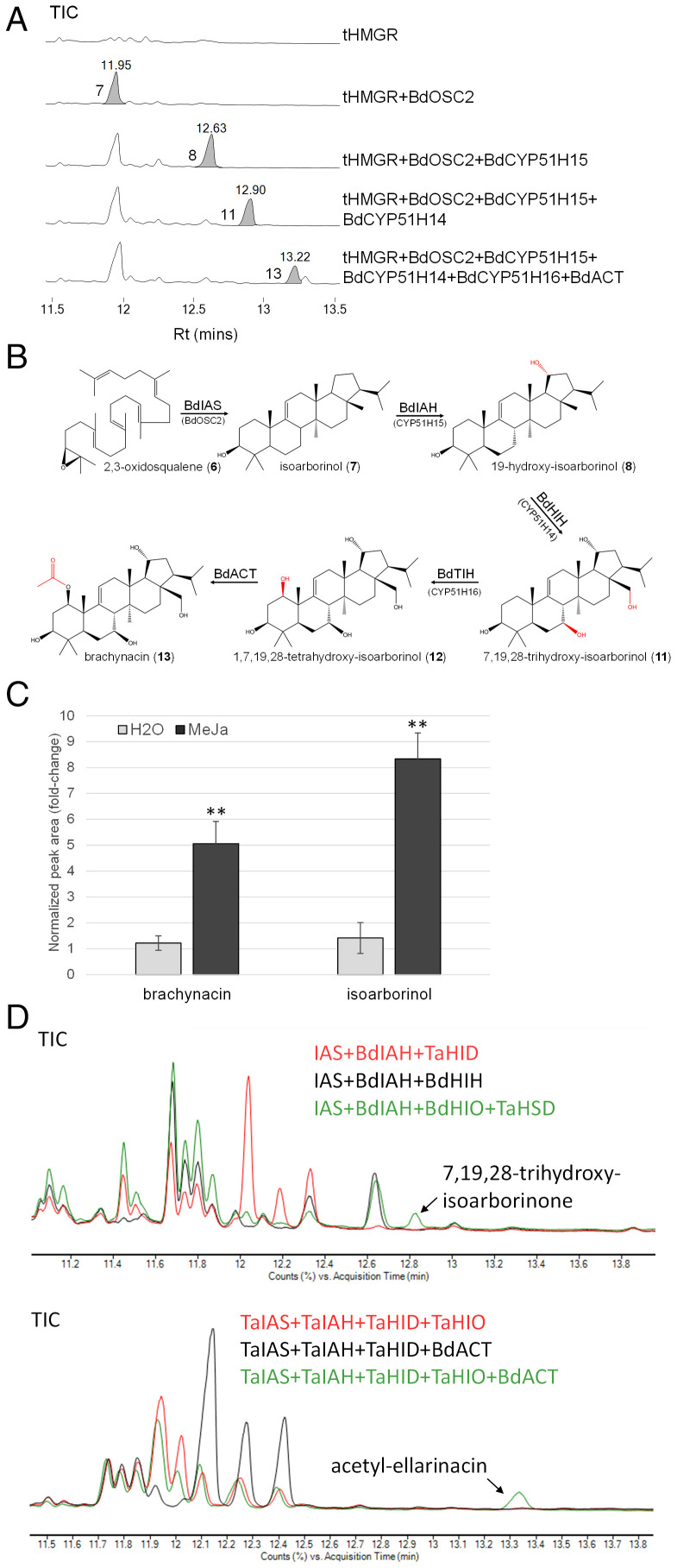
*B. distachyon* BGC produces the isoarborinol-derived triterpenoid, brachynacin. (*A*) GC-MS traces for *B. distachyon* cluster genes transiently expressed in *N. benthamiana*. Marked peaks were identified as isoarborinol (**7**), 19-hydroxy-isoarborinol (**8**), 7,19,28-trihydroxy-isoarborinol (**11**), and brachynacin (**13**) (494.5). (*B*) Assigned structures and predicted biosynthetic pathway of brachynacin in *B. distachyon*. BdACT, 1,7,19,28-tetrahydroxy-isoarborinol acetyltransferase; BdIAH, isoarborinol hydroxylase; BdIAS, isoarborinol synthase; BdHIH, 19-hydroxy-isoarborinol hydroxylase; BdTIH, 7,19,28-trihydroxy-isoarborinol hydroxylase. (*C*) Relative abundance of isoarborinol and brachynacin in TMS-derivatized extracts of *B. distachyon* leaves treated with MeJa or H_2_O for 12 h. Relative quantification is based on normalized peak areas in GC-MS analysis of four biological replicates. Means of four biological replicates ± SEM are shown. Asterisks denote *t* test statistical significance. **P < 0.01. (*D*) GC-MS TICs of *N. benthamiana* leaves transiently expressing combinations of wheat and *B. distachyon* genes.

Since gene expression analysis suggested an active BGC, we next investigated the functions of the cluster genes by transient expression in *N. benthamiana* (Fig. 6*A*). GC-MS analysis revealed that BdOSC2 and CYP51H15 exhibit the same activities as their respective wheat orthologs, namely production of isoarborinol and its 19-hydroxylated derivative (*SI Appendix*, Fig. S28). Coexpression of BdOSC2 and CYP51H15 together with the two additional CYP51s and the BdACT acyltransferase resulted in formation of the putative BGC end product, with a mass signal of [M+H−H_2_O = 515.3] (*SI Appendix*, Figs. S29 and S30). This product was purified following large-scale transient expression of the *B. distachyon* cluster genes in *N. benthamiana* and found by ^1^H and ^13^C NMR analyses to be an isoarborinol-derived triterpenoid with hydroxyl groups on the C7, C19, and C28 carbons and an acetoxy group on the C1 carbon (*SI Appendix*, Fig. S31 and Table S8). The assigned structure allowed the full elucidation of the biosynthetic pathway from 2,3-oxidosqualene, in which BdOSC2 and BdCYP51H15 generate 19-OH-isoarborinol, BdCYP51H14 hydroxylates the C7 and C28 carbons to give 7,19,28-trihydroxy-isoarborinol (**11**), and BdCYP51H16 hydroxylates the C1 carbon to give 1,7,19,28-tetrahydroxy-isoarborinol (**12**), which is further acetylated by BdACT (Fig. 6*B*). This compound was named brachynacin (**13**). The occurrence of brachynacin in *B. distachyon* was verified by GC-MS analysis of leaf extracts. As for ellarinacin in wheat, the relative abundance of brachynacin, as well as of isoarborinol, were found to be significantly higher in MeJa-treated vs. nontreated detached leaves (Fig. 6*C* and *SI Appendix*, Fig. S32).

### Combination of Ellarinacin and Brachynacin Biosynthetic Genes Yields Novel Compounds.

The similarities between the ellarinacin and brachynacin BGCs indicates that they possibly originate from a common ancestral cluster but have evolved to produce different end products, through duplication and neofunctionalization of CYP51H enzymes and recruitment of additional modifying enzymes (TaHSD and BdACT, respectively). The evolution of these two pathways may have been facilitated by a degree of promiscuity that enabled the pathway enzymes to accept different substrates. Indeed, coexpression of different combinations of genes from the two BGCs in *N. benthamiana* did yield new products. Expression of TaHSD with BdCYP51H14 and BdCYP51H15 led to production of a new compound with a molecular mass [M+H−H_2_O = 455.2; M+H−2H_2_O = 437.2], matching the expected product, 7,19,28-trihydroxy-isoarborinone (Fig. 6*D*). Likewise, expression of BdACT with the ellarinacin cluster resulted in formation of a new compound identified as acetyl-ellarinacin, based on its molecular mass signal [M+H = 497.3] and the fact that its formation required expression of the entire wheat BGC (Fig. 6*D* and *SI Appendix*, Figs. S33 and S34). The formation of novel compounds through combining genes from wheat and *B. distachyon* BGCs demonstrates the promiscuous nature of enzymes encoded by genes within these two clusters.

## Discussion

Despite the importance of wheat as a food and feed crop, our understanding of the molecules that it produces in response to biotic stress remains limited. Conversely, various phytoalexins and their biosynthetic pathways have been well-characterized in other major cereal crops such as rice, maize, oat, and sorghum (54, 55), and serve as potential targets for crop improvement (54, 56). Research into specialized metabolism in wheat has until recently been hindered by the lack of a fully assembled genome. The availability of a newly assembled genome coupled with the vast amount of available transcriptomic data now opens up opportunities to deploy genomics-driven approaches for discovery of novel metabolic pathways in wheat, including those implicated in plant defense. Here, utilization of wheat genomic and transcriptomic resources has enabled us to identify pathogen-induced biosynthetic pathways for flavonoids, diterpenes, and triterpenes. These pathways are driven by sets of genes that are colocalized in the wheat genome, forming six BGCs, including two pairs of homologous clusters. Strikingly, analysis of the bread wheat genome with the BGC mining tool plantiSMASH (57) reveals that the six BGCs described in this work are only a fraction of the total of 239 candidate BGCs found in this genome (Dataset S3). It remains to be seen how many of the other predicted BGCs encode functional biosynthetic pathways.

We identified a cluster on wheat Chr.5D, BGC 4(5D), encoding a biosynthetic pathway for O-methylated flavonoids. Although flavonoids form a ubiquitous and highly diverse class of compounds in plants, BGC 4(5D) is the only identified and functionally validated flavonoid BGC to date. Examination of the *A. tauschii* genome reveals a conserved cluster, also located on Chr.5D (*SI Appendix*, Fig. S35), indicating that this cluster was formed prior to the hybridization events between *A. tauschii* and tetraploid emmer wheat. Further research will be needed for full structural assignment of the product encoded by this flavonoid BGC. Additionally, two different types of diterpene-producing clusters [BGC 1(2A/2D) and BGC 2(2B)] were identified on group 2 chromosomes, one of which is syntenic to the rice momilactone cluster. However, the pathways encoded by these syntenic BGCs diverge in their early steps due to differential CPS activities (i.e., producing *syn*-CPP or normal-CPP). Notably, momilactone BGCs were also found in genomes of barnyard grass (*Echinochloa crus-galli*) (58) and the bryophyte *Calohypnum plumiforme* (59).

The orthologous relationships between the *KSL*, *CPS*, and *CYP99* genes and the occurrence of the clusters in syntenic loci in the wheat, rice, and barnyard grass genomes indicate that these clusters arose from a common ancestral BGC, formed prior to the divergence of the PACMAD and BOP clades, the two major lineages of the Poaceae family (60). However, the more closely related functions of the BGCs from rice (BOP) and the more distant barnyard grass (PACMAD) raise interesting questions. Specifically, as opposed to the wheat type 1 BGCs that appear to produce normal-CPP–derived diterpenoids, the rice and barnyard grass clusters both produce momilactones, a capability dependent on acquisition of *syn*-CPS rather than normal-CPS activity, as well as the recruitment of the reductase (MAS) genes. Independent formation by convergent evolution of barnyard grass and rice momilactone BGCs (as well as in distant bryophytes) was previously suggested (59). However, the discovery of the momilactone-like cluster in wheat implies that a more extensive survey of grass genomes may be required in order to definitively track the intriguing evolutionary development of this cluster.

**Fig. 7.**
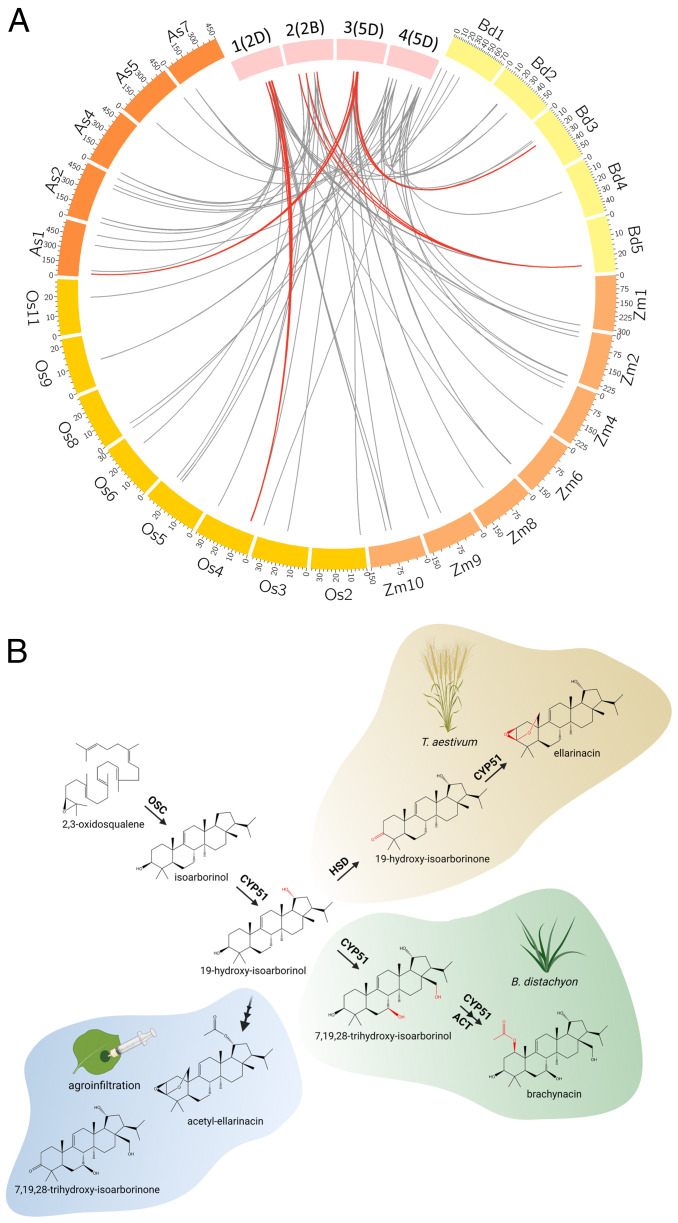
Phylogenetic and chemical divergence of arborinane-type biosynthetic gene clusters. (*A*) Circos plot depicting genomic locations of the closest matching homologs of coexpressed genes from wheat BGCs 1(2D), 2(2B), 3(5D), and 4(5D) on chromosomes of *B. distachyon* (Bd), diploid oat *A. strigosa* (As), maize (Zm), or rice (Os). Gray: links to homologs dispersed across the analyzed genomes. Red: links where two or more matching homologs from different gene families colocalize in the analyzed genomes. Cluster 1(2D) is linked to the momilactone BGC on rice Chr.4; cluster 2(2B) is linked to a putative terpene BGC in *B. distachyon* Chr.5; cluster 3(5D) is linked to the brachynacin BGC in *B. distachyon* Chr.3 and OSC–CYP51H pair in oat Chr.1; cluster 4(5D) homologs are dispersed in all grass genomes included in the analysis. (*B*) Clustered biosynthetic pathways for arborinane-type triterpenoids in wheat and *B. distachyon* diverge from a common precursor, 19-hydroxy-isoarborinol, due to neofunctionalization of CYP51 enzymes and recruitment of other gene families. These pathways can be further artificially “diverged” by recombinant expression of combined genes from the two clusters. The image was created with BioRender.

We did not identify a homologous cluster to BGC 1(2A/2D) in the other grass genomes that we analyzed, which included *B. distachyon*, oat, barley, and maize. A putative terpene cluster homologous to cluster 2(2B) was, however, found in *B. distachyon* (Fig. 7*A* and *SI Appendix*, Table S9). Notably, several wheat *KSL* genes were previously shown to be induced by UV irradiation, including *TaKSL1* found in cluster 1(2A) and *TaKSL2/TaKSL3* from cluster 2(2B) (32). Physiological functions for wheat diterpenes in response to biotic and/or abiotic stress, similar to those observed for diterpenes in rice and maize, could thus be hypothesized (24). However, expression analysis of the genes comprising the 1(2A/2D) and 2(2B) BGCs in wheat RNA-seq data does not reveal any notable induction in response to various abiotic stresses, with the exception of a single *KSL* gene on cluster 2(2B), TraesCS2B02G445900, which exhibits mild induction under cold stress (*SI Appendix*, Figs. S36 and S37). Therefore, a role for the wheat diterpene clusters in response to abiotic stress cannot be currently postulated on the basis of these gene expression data.

Finally, a pathogen-induced cluster [BGC 3(5A/5D)] for a novel isoarborinol-derived triterpenoid, ellarinacin, was found on Chr.5 of the A and D genomes, which is conserved in its wild ancestral species, wild emmer wheat and *A. tauschii*, and is composed of genes co-opted from sterol primary metabolism. Interestingly, we found the ellarinacin BGC to be more highly induced by nonadapted strains of several fungal pathogens. This cluster may thus form part of a wider set of defense responses found to be actively suppressed in wheat by adapted fungal pathogens, presumably via suppression of plant immune response regulators by pathogen-secreted effector proteins (45). The reduced induction of the ellarinacin BGC by host-adapted strains also possibly alludes to a more specific function for ellarinacin, in response to nonhost interactions. A similar case was observed in rice, in which *syn*-CPP–derived diterpenoids were implicated in increased resistance to nonadapted pathovars of the fungal pathogen *Magnaporthe* spp. but not to rice-adapted strains of *Magnaporthe oryzae* (61). However, this diminished influence on susceptibility in host interactions could also be due to the ability of *M. oryzae* to metabolize momilactones, rather than as a result of reduced induction (61, 62).

Microsynteny and homology searches in other grasses revealed the occurrence of a pathogen-induced cluster in *B. distachyon*, homologous to the ellarinacin cluster in wheat. The pathways encoded by these two clusters diverge from the shared intermediate 19-hydroxy-isoarborinol by neofunctionalization of duplicated CYP51 genes, together with recruitment of additional genes for other enzyme families. Recombinant expression experiments showed that at least some components of these clusters are interchangeable, enabling production of molecules that are not produced by either cluster alone (Fig. 7*B*), and pointing to the importance of enzyme promiscuity in facilitating chemical diversification.

In summary, a genomics-driven approach has enabled us to rapidly identify and characterize compounds and biosynthetic pathways in bread wheat. These clusters are highly induced in response to infection by various fungal pathogens and PAMPs, suggesting a broad-spectrum role for these clusters in chemical defense against biotic stresses. Correspondingly, coexpressed genes within these clusters were found to be part of a shared regulatory network that includes various transcription factors predicted to be associated with biotic stress responses. Future work is needed to further understand the interactions and potential contribution of each of these pathways to protection from pathogens in wheat and other grasses, as well as to elucidate the regulatory network which governs the expression of these pathways.

## Materials and Methods

### Computational Analyses.

Methods for regulatory network analysis, coexpression analysis, microsynteny analysis and pairwise alignments are detailed in *SI Appendix*, Materials and Methods.

### Semiquantitative RT-PCR in Wheat Tissues.

RNA from all samples was extracted using TRIzol reagent (Sigma-Aldrich), according to the manufacturer’s protocol. RNA (5 µg total) of each sample was used in 20-µL reverse-transcription reactions with SuperScript III (Thermo Fisher Scientific), according to the manufacturer’s protocol. Thirty-cycle PCR containing 0.2 µL complementary DNA (cDNA) template in a 10-µL total reaction volume was performed and analyzed on 1% agarose gels. Oligonucleotides used are specified in *SI Appendix*, Table S10. Specific details of wheat tissue sampling and preparation for RT-PCR analysis are detailed in *SI Appendix*, *Materials and Methods*.

### Semiquantitative RT-PCR in *B. distachyon* Tissues.

Tissues for RT-PCR were sampled from greenhouse-grown *B. distachyon* (Bd21) plants or 2-d-old seedlings grown on a Petri dish in a growth cabinet (28 °C, 16-h photoperiod). RNA was extracted using the RNeasy Plant Mini Kit (Qiagen), treated with DNase (RQ1; Promega), and used for cDNA library preparation with GoScript reverse transcriptase (Promega), using oligo(dT) primers. All PCR was carried out on an Eppendorf Mastercycler Pro thermal cycler, for 40 cycles with 55 °C annealing temperature, using GoTaq G2 Green Master Mix (Promega) and oligonucleotides detailed in *SI Appendix*, Table S10. Electrophoresis of PCR products was done on EtBr-stained 1% agarose gels and photographed on a Gel Doc XR instrument (Bio-Rad).

### Plant treatment with elicitors and pathogens.

Methods for inoculation of detached wheat leaves with powdery mildew, treatment of detached wheat leaves with elicitors, and treatment of *B. distachyon* with methyl jasmonate are detailed in *SI Appendix*, Materials and Methods.

### Quantitative Real-Time PCR of Wheat.

For quantitative real-time PCR analysis of wheat leaves inoculated with powdery mildew or treated with elicitors, three biological replicates, each containing three leaf samples, were tested for each time point. Methods for RNA extraction, reverse transcription, and quantitative real-time PCR analysis are specified in *SI Appendix*, *Materials and Methods*.

### Quantitative Real-Time PCR of *B. distachyon.*

For quantitative real-time PCR of *Fusarium*-infected *B. distachyon* plants, *B. distachyon* accession Bd3-1 plants were treated with *F. graminearum* isolate PH1. For the *Fusarium* root rot (FRR) experiment, three biological replicates were used, each consisting of roots from 10 plants. For the *Fusarium* head blight (FHB) experiment, three biological replicates were used, each consisting of three spikes from different plants. Methods for sample preparation, RNA extraction, reverse transcription, and quantitative real-time PCR analysis are further specified in *SI Appendix*, *Materials and Methods*.

### Generation of DNA Constructs.

Gene cloning methods and full coding sequences and GenBank accession numbers of all genes used in this study can be found in *SI Appendix*, *Materials and Methods*. Oligonucleotides used for amplification and subcloning are specified in *SI Appendix*, Table S10.

### Agroinfiltration-Mediated Transient Expression in *N. benthamiana*.

Plant expression vectors were transformed into *Agrobacterium tumefaciens* GV3101 via electroporation. Agrobacteria cultures were grown overnight in 28 °C in Luria-Bertani (LB) media and resuspended in MMA buffer (10 mM MgCl_2_, 10 mM 2-[*N*-morpholino]ethanesulfonic acid, pH 5.6, 100 µM acetosyringone) to an optical density at 600 nm (OD_600_) of 0.2. For coexpression of several genes, OD_600_ 0.2 cultures of strains expressing different genes were mixed 1:1 prior to infiltration. Cultures were infiltrated by syringe into leaves of 5-wk-old greenhouse-grown *N. benthamiana* plants. The plants were further maintained in the greenhouse after infiltration. Infiltrated leaves were harvested 5 d post infection, freeze-dried, and ground.

### GC-MS Analysis of Diterpenes and Triterpenes from *N. benthamiana* and Grass Leaf Extracts.

GC-MS analysis was performed using an Agilent 7890B instrument with a Zebron ZB5-HT Inferno column (Phenomenex). Specific methods for the extraction and analysis of diterpenes and triterpenes are detailed in *SI Appendix*, *Materials and Methods*.

### LC-MS Analysis of *N. benthamiana* Leaf Extracts.

Leaf extracts were analyzed by reverse-phase high-performance liquid chromatography on a Shimadzu LCMS-2020 single-quadrupole mass spectrometer. High-resolution MS analysis of the metabolites was carried out on a Q Exactive instrument (Thermo Scientific). Specific methods for the extraction and analysis of flavonoids and triterpenes are detailed in *SI Appendix*, *Materials and Methods*.

### Large-Scale Agroinfiltration, Extraction, and Purification of Triterpenoids.

Vacuum-mediated large-scale agroinfiltrations of *N. benthamiana* plants and downstream extraction and purification of triterpenoid products were based on a previously described method (36, 63). Specific methods for extraction and purification of the metabolites are detailed in *SI Appendix*, *Materials and Methods*.

### General Considerations for NMR.

NMR spectra were recorded in Fourier transform mode at a nominal frequency of 600 MHz for ^1^H NMR, and 150 MHz for ^13^C NMR (unless specified otherwise), using the specified deuterated solvent. Chemical shifts were recorded in ppm and referenced to the residual solvent peak or to an internal tetramethylsilane (TMS) standard. Multiplicities are described as the following: s, singlet; d, doublet; dd, doublet of doublets; dt, doublet of triplets; t, triplet; q, quartet; quint, quintet; tquin, triplet of quintets; m, multiplet; br, broad; appt, apparent. Coupling constants are reported in hertz as observed and are not corrected for second-order effects.

## Supplementary Material

Supplementary File

Supplementary File

Supplementary File

Supplementary File

## Data Availability

All study data are included in the article and/or supporting information.

## Change History

September 1, 2022: A new figure (Figure 1) has been added as a Correction; please see accompanying Correction for details.
