# Supplementary material for: Pathogen-induced biosynthetic pathways encode defense-related molecules in bread wheat
Source: Proc Natl Acad Sci U S A. 2022 Apr 11;119(16):e2123299119. doi: 10.1073/pnas.2123299119 (PMC9169793; doi:10.1073/pnas.2123299119)
Supplement: Supplementary File [file pnas.2123299119.sapp.pdf]

Supplementary Information for:

Pathogen-Induced Biosynthetic Pathways Encode Defense-Related Molecules in Bread Wheat

Guy Polturak[1*], Martin Dippe[1,2], Michael J. Stephenson[1], Rajesh Chandra Misra[1], Charlotte Owen[1], Ricardo H. Ramirez-Gonzalez[3], John F. Haidoulis[3], Henk-Jan Schoonbeek[3], Laetitia Chartrain[3], Philippa Borrill[1], David R. Nelson[4], James K.M. Brown[3], Paul Nicholson[3], Cristobal Uauy[3] and Anne Osbourn[1*]

[1]Department of Biochemistry and Metabolism, John Innes Centre, Norwich NR4 7UH, United Kingdom

[2]Current address: Department of Bioorganic Chemistry, Leibniz Institute of Plant Biochemistry, D-06120 Halle (Saale), Germany

[3]Department of Crop Genetics, John Innes Centre, Norwich NR4 7UH, United Kingdom

[4]Department of Microbiology, Immunology and Biochemistry, University of Tennessee Health Science Center, Memphis, TN 38163

[*]To whom correspondence should be addressed: Email: guy.polturak@jic.ac.uk; anne.osbourn@jic.ac.uk

SI Materials and Methods. Coding sequences of genes used in this study. Synthesized genes are asterisked.

```
>TaCHS1* (TraesCS5D02G488700; GenBank ON108659)

ATGGCCGCCGCTGTTACCGTAGACGAAGTAATGAAGATGCAGAGAGCGCAGGGCCCCGCAACTGTGTTAGCAATA
GGTACGGCCACTCCACATAACTGCGTTTACCAAGCAGACTACGCTGACTACTACTTTAGGATCACCAATAGTGAA
CATCTGACAGATCTAAAAGACAAATTCCAAAGAATGTGTGACCGAAGTATGATCAGAAAGCGTTACATGCATGTG
ACCGAGGAGATTCTCAAAGAGAACCCCAACGTTTGCGCTTACATGGCGCCTAGCCTAGATGCTCGCCAAGACATG
GTGCTTGGTGAGGTTCCCAAGCTGGGGAAAGAGGCTGCTCATAAGGCAATCAAGGAATGGGGACAGCCTCTCTCC
AAGATCACTCACCTCGTTTTCTGCACAACGAGTGGAGTGGACATGCCAGGTGCCGACTATCAGCTGACTAAGATG
CTTGGTCTGCACCCATCTGTAAGAAGGGTGATGTTATACCAGCAAGGTTGCTTTGCTGGTGGTACAGTGCTACGT
GTGGCTAAGGACTTAGCCGAGAACAACAGAGGAGCTCGTGTGCTTGTAGTGTGCTCCGAGATAACAGCAGTTACT
TTTCGAGGCCCGTGTGACACCCAGTTGGATTCCATGGTCGGACAGGCTTTGTTTGGCGATGGCGCAGCTGCAGTC
GTTGTGGGCGCTGACCCCGACGTATTGGTGGAACGGCCCTTATTCCAACTTGTATCTGCTTCCCAGACCATATTA
CCAGACACAGATGGTTTCATCAAGGGCCACCTTCGAGAGGTTGGACTTACATTCCACCTTCACAAGGATGTACCT
GTTGCGATATCAAAGAACATACAACAAGCACTTGAGGACGCGTTCGCACCACTCGGCATCGATGACTGGAACTCT
ATATTTTGGGTAGCCCACCCGGGTGGCCCAGCCATTTTGGACATGGTCGAGGCTGAGGCTAAGCTTGACAAACGA
AGAATGAGGGCAACTAGGCACATATTGTCTGAGTACGGGAACATGTCCAGCGCTTGTGTGTTGTTCATCTTAGAC
GAGATGAGGAAGAGGTCTGCAGAGGACGGCCACGCTACCACAGGCGAGGGTCTAGACTGGGGCGTCCTGTTTGGA
TTCGGACCCGGTCTTACGGTAGAGACAGTTGTCCTACACAGTGTGAGGACAACAGCCGCTTGA


>chi-D1* (TraesCS5D02G489000; GenBank JN039039)

ATGGCAGTTTCCGAACTAGAAGTTGATGGTGTTGTATTTCCACCCTTAGCAAGACCACCAGGAAGCGAACATGCG
CATTTCTTGGCTGGTGCCGGTGTTAGAGGCATGGAAATTGGTGGGAATTTTATTAAATTTACAGCTATAGGAGTT
TATTTGCAAGCTGATGCTGCTGTTAGTGCTCTTGCGGCTAAATGGGCTGGAAAACCTGCAGCGGATTTAGCATCA
GATGCAGCTTTCTTTCGTGATGTAGTTACTGGAGAATTTGAAAAGTTTACTCGGGTTACTATGATTTTGCCACTT
ACTGGTGCTCAATATTCTGATAAAGTCACCGAAAATTGTGTTGCGTATTGGAAAGCAACAGGGGTCTATACTGAT
GCTGAAGCAGCTGCTGTTGATAAATTTAAAGAAGCATTTGGCCCTCATAGTTTTGCTCCTGGGGCTTCTATTTTA
TTTACTCATTCACCTGCTGGACTTCTGACAGTGGCTTTTAGCAAAGATTCCTCAGTTCCAGAATCTGGTGGTGTT
GCTATTGATAATGCTAGGTTGTGTGAAGCAGTACTTGAATCTATTATAGGTGAACATGGTGTTTCTCCCGCAGCT
AAACTTTCCTTAGCAACACGTGTTGCAGAACTTCTTGAAGGTGCTGCACTGGCAGGTGGTGAACCTGCTGCTGAA
GCAGTCAGTGTCTCAGTATGA


>TaCYP71C164_5D (TraesCS5D02G488500; GenBank ON108660)

ATGGAAGATCTCGTGAAGAAACCCATGCCAGAGACACCTCCGCGGGCATTGTTTCTGTTCCTATTCTTCCTCTTC
CTCTTGTTTGTAAGCTGGTTCACTGGAAAGACAAGAAAAATGCAGCATCAGCAGCGAGAAAACAACCACCTCCCG
CTCCCGCCTTCGCCGCCGGCACTGCCCATCATTGGTCACCTGCACCTCGTCGGCTCCCTCCCGCACGTCTCTCTC
CGCAGCCTTGCCAGGAAGTATGGTCCCGACATGATGCTTCTGCGCCTGGGCGCCGCGCGGACCCTCGTGGTGTCG
TCGCTGCGTGGCGCAGAGGCAGTGCTGCGCACCCACGACCACATCTTGGCGTCGCGGCCCAGCTCCGTAGTCTCC
GACATCCTCACGTACGGCTCGTCTGATATGGCCTTCGCGCCATACGGCGAGTACTGGCGGCAGGTGAGGAAGCTG
GTCACCACCCACATGTTGAGCGTTAAGAAGGTGCAGTCTTTCCGCAGCGCTGCCATGGAGGAGGTGAGCTTGGTG
ATGGCCAAGATTAATGAGGTGGCCACAACCGATGGTACGGTGGACATGAGAGAGCTTCTCAGCTTATTCGCGAAT
GACATGGGGTGCCGCATCGTATCGGGAAAGTTCTTCCTAAAAGATGGACAGAGCAAGTTGTTCCGGGACCTCACC
AACGATACCTCACGGCTGTTAGGAGGGTTCAACGTGGAGGAGTACTTCCCAGGATTGGCAAGGGTAGGAGTGTTT
AAGAGGGCAGTTTGTGCCCAAGCCGAGAGAGTGAGGAATAGATGGGCTGATCTGCTAGACAAGGTGATCGACGAT
CATGTGAGCAAGGATAAGTCAACGTTTGATCACGAGGATGGTGATTTTGTAGATATTCTGTTGACTTTTCAGCAC
GAGTATGATCTCACAAGAGAGCACATGAAAGCTATCCTGATAGATGTATTTGCCGGTGCAACAGACACATCAGCT
AACGCCCTCGAATTCACCTTGGCCGAGCTCATGAGGAAGCCACGTGTGATGGGGAAGCTACAAGCTGAGGTGAGG
AGTAACGTACCCCAGGGACAAGAAATTGTCAGTGAAATCAACGTGAACAATATGGCATACCTAAGAGCAGTCATA
AAGGAGTCGCTTCGACTGTATCCTATTGCGCCTCTCCTTGCTCCACACCTTGCCATGGATGACTGCAATATTGAT
GGATATATGGTTCCTGCTGGGACGCGTGTCATGGTCAATGCATGGGCCATTGCTAGGGACTCGAGCTTGTGGGAG
GACGCAGAAGAGTTCATACCTGAAAGATTTACAGATGAAGGCAGCGCTATGCATGTCAATTTCAAGGGGAATGAT
TTCCAGTTCTTGCCATTCGGGGCAGGACGAAGGATGTGCCCCGGTATGAACCTCGGAATCGCTAATGTTGAGCTT
ATGTTGGCAAACCTCATGTACCATTTTGACTGGGAACTTCCGCTTGGAGTTGAGAGAGAAGACATTGATATGACA
GCGGTGTTTGGGCTAACCATGCGCCGGAAGGAGAAACTATTGTTAATTCCAAAATCCTGGATGTAG


>TaCYP71F53_5D (TraesCS5D02G487900; GenBank ON108661)
```

ATGGAGGGTTGGTTAACCTTATGTTTCATAGCCGTATCGACGTTAGTGGCCGTTTGGTTTTCCGGTGGCAAGAGC
AAGCCCAAGAAGCATCTGCCTCCTGGGCCATGGACTCTCCCGGTCATCGGCAGCCTCCACCACGTCCTCAGTGTC
CTCCCACACCGCACCATCACGGAGCTGTGTCGCCGGCATGGGCCACTGATGCTCCTCAAGCTAGGTGAGGTTCCA
ACCGTGGTAGTCTCGAGCGCCGAGGCGGTGGAGCAGGTGATGAAGACCAATGACATCGCCTTCTCGTACCGGCGG
ACCACCGAGTTGCAGGACATCGTCGGCTTCGGCGGCAAGGGCATCATCTTCGCCCCCTACGACAACCGCTGGCGC
CAGATGCGCAAGGTCTGCATCATGGAGCTCCTCAATTCCAAGCAGGTGAAGCGCATGGAAGACATCAGGGCCGAG
GAGGTGGGCCGCCTCCTCCGCTCAATCATCACGGCCACAGCTGTGGGCGCTACCGTCAACATCAGCCAGAAGGCT
GCGGCGCTCAGCAATGCCGTGGTGACGCGGGCGGTGTTCGGCGGCAAGTTCGCACGGCAGGAGGAGTACCTTCGC
GAGATCAACCAACTCCTAGAGCTGTTGGGAGGATTCTGCCTTGTCGACCTCTTCCCGTCGTCGCGGCTGGTGCGG
TGGTTCAGCACTTGCGAGCGCCGCACAAAGAAGAGCTGCGACCTCATCCAGCACATCATCACCGGGGTGCTTGAC
GAGCGCAAGGTGGTGCGAGCTGCCGGTGACGGCGCCTGCAGCACCGACGATGAGGACCTACTGGACGTGCTGCTC
AGGCTGCAGGAAGAGGACTCGTTGGCATACCCTCTAACGACAGAGAATATAACTACCGTCTTGTTTGACATCTTT
GCAGCTGGCACGGATACTACAGGAACCGCTTTGGAGTGGGCTATGTCAGAACTCATATGTCATCCTGAAGCTATG
GCTAAGGCACAATTAGAGGTTCGAGAGGTACTGGGTCATGGCCGAGCTATCATTGGCAATAGCGATCTTGCAAAA
CTCCACTACTTGCGGATGGTCATCAAGGAGGTTCTTAGATTGCATCCACCTGGTGCTCTACTTCCCCGCAAGACT
AGAGAGGACTGCAAAATTATGGGTTATGACATGCTTAAAGATACAAATATATACATTAATGTCTTCGCAATTTCC
CGAGATCCTCGATATTGGAACAATCCTGAAGAGTTTAATCCAGGAAGGTTTGAGAACAATAACGTGGATTATAAT
GGGAATTCTTTTGAATTCACTCCTTTCGGAGGTGGGCGACGGCAA
TGCCCTGGGATAGCATTCGCCACGTCACTTTTGGAGATCACTTTAGCAAACTTTTTATATCACTTCAACTGGATG
CTTCCTGGCGAAGCCAGCTCAGCGTCACTGGATATGTCTGAGAAATTTGGGTTCACCATAGGTAGAACATCTAAT
CTGCACCTCAAGGCTATTCCATATGTACGCTCCACTATATAG

>TaOMT3 (TraesCS5D02G488800; GenBank ON108662)

ATGGGCTCCACTGCCGTGGAGAAGGTCGCTGTCGCCACTGGCGACGAGGAGGCGTGCATGTACGCGGTGAAGCTT
GCAGCGGCATCTATCCTTCCAATGACCCTCAAGAACGCCATCGAGCTGGGCATGCTCGAGATCCTCGTGGGTGCC
GGCGGGAAGATGTTGTCACCTTCAGAGGTGGCAGCGCAGCTTCCGTCGAAGGCCAACCCGGAGGCACCGGTTATG
GTGGACCGCATGCTGCGGCTGCTGGCATCGAACAACGTCGTGTCATGCGAGGTGGAGGAAGGTAAGGACGGCCTC
CTCGCCCGTCGATACGGCCCCGCGCCCGTGTGTAAGTGGCTCACACCCAACGAGGACGGCGCATCCATGGCTGGG
CTGCTCCTCATGACCCACGACAAGGTCACTATGGAGAGCTGGTATTATTTGAAGGACGTGGCCCTTGAAGGCGGC
CAACCATTCCACAGGGCGCACGGGATGACGGCGTACGAGTACAACAGCACAGACCCACGCGCTAACTGCTTGTTC
AACGAGGCCATGCTTAACCACTCCACCATCATCACCAAGAAGCTCCTCGAGTTCTACAGGGGCTTCGACAACGTC
GAGACCCTCGTGGATGTCGCCGGTGGCGTTGGTGCCACAGCCCACGCCATCACCTCAAAGTACCCGCACATCAAG
GGGGTAAACTTCGATCTCCCGCATGTCATATCCGAGGCGCCGCCCTACCCTGGCGTGCAGCACATCGCCGGTGAC
ATGTTCAAGAAGGTGCCCTCCGGCGATGCTATCCTCCTGAAGTGGATCCTCCACAACTGGACCGACGATTACTGT
ATGACTCTTCTGAGGAACTGCTACGATGCGTTGCCCATGAATGGCAAGGTGGTCATCGTGGAGGGCATCCTGCCG
GTGAAACCAGATGCAATGCCCAGCACGCAGACGATGTTCCAGGTCGACATGATGATGCTGCTGCACACCGCAGGC
GGCAAGGAGAGGGAACTGAGCGAATTTGAAGAGCTAGCGAAGGGCGCTGGGTTCAGCACAGTCAAGACCAGCTAC
ATCTACAGCACCGCATGGGTCATTGAGTTCGTCAAATAG

>TaOMT6 (TraesCS5D02G488300; GenBank ON108663)

ATGGCGCCCACCCAAGGCAAGCAGAGTTCTCAGGATTTGCTCGAGGCTCAAGTTGACCTTTGGCACCATTCATTG
GGATTTGTCAAGTCCATGGCACTCAAATGTGCAATGGAACTGCAAATCCCTAACACCATCCAACACCATGGTGGG
GCTATGACTCTTTCTGAGCTGGCCACAAAGACAGGGATCCATCCGTCTAAGCTTCCCCCCTTAGGGCGACTCATG
CGTGTACTCACCGTATCAGGCATCTTTGTTGTCCATGACTCAGCCTCGGGAGACAACAAGGAGGCTGTGTATGGA
CTTACCCCAAGCACGTGCCTCCTCGTCAGCGATGAAGTTAAATCAAACCTATTTCCCATTCTGTCTTTGTTCCTT
GATTCAACTGCCATTACACCCTTTTTCGGCATGCACTCATGGTTCCTAGATGAGCATTCCACGTCCCTGTTCAAA
AAGGCTCACGGCCTTAACGTTTGGGAGATGGCTGACCAGAACAATGCTTGCAACGAGCAAATCAATAACGCGATG
GTTTCTGATAGTAACTTTTTCATCAATATCCTCTTGAGGGAGTGTGGTGATGTATTTCTTGGCATAAACTCACTT
ATTGATGTTGCGGGAGGACACGGTGGAGCTGCTAGGGCAATTGCTATGTCGTTCCCCCAGATGAAATGCACTGTG
TTGGATCTCCCTCATGTGGTTGCTGGAGCTCCCAGTGATGCCCATGTGTCATTTATTTCTGGCGATATGTTTAAG
TACATTCCACCGGCGGATGCTCTTTTCCTGAAGTGCATTTTTCACGATTGGTGTGATGAAGACTGTGTCAAGCTA
CTGAAGAATTGCAAGGAAGCTATCCCTCCCAGAGATGCTGGTGGAAAGGTGATAATCGTTGATATGGTGGTTGGA
TCTGGGCCAGATGACATTGTAACAAGAGAGACACAGGTTTTCTATGATCTCTTTATCATGGGTGCTGAGGGGATT
GAGCGAGAGGAATTTGAGTGGAAGAAGATATTTATGGAAGCTGGGTTCAGCAATTACAAAATTCTATCAGTGCTG
GGAGTTAGATCTGTTATTGAGCTCTACCCTTGA

>TaOMT8 (TraesCS5D02G488900; GenBank ON108664)

ATGGGTTCTATCTCCGACGACGAGGCGGCTTGCATCTACGCGATGCAGCTGGCAGCGGCAGCTGTCTTGCCGATG
ACGCTCAAGAACGCCATCGAGCTGGGCATGATCGAGATCCTCGTTGGCGCTGGCGGGAAGATGCTTTCGCCGTCT
GAGGTGGCCGCCCAGCTGCCGTCGACGGCCAACGCGGAAGCACCGGCCATCGTCGACCGCATGCTGCGGCTGCTG
GCATCGCACAACGTTGTGTCGTGCGAGGTGGAGGAAGGTAAGGACGGCCGCCTCGCACGCCGGTACGGCACCGCG
CCGGTGTGCAAGTGGCTCAGCCCCGACGAGGACGGCGTGTCCTTGGCCCCGATGGTCCTCTTGAACGACAAGGTC
ATGCTGGAGAGCTGGTACCATTTGAAGGACACTGTCCTTAACGGTGGCCTGCCATTCGAAAAAGCGTACGGAATG
ACAGCGTTTGATTACCAAGGAACGGACCCGCGCTTTAATCGCGTCTTCAATGAGGCCATGAAGACCCACTCCATG
ATCATCACCAAGAAGCTCCTGGAGTTCTACAAGGGCTTCGATGGTGTGGGCACCCTCGTGGACGTCGGTGGCGGT
GTGGGCGCCACCATCCACACCATCCTTTGCAGGTACCCAAGCATCGAGGGGGTCAACTTCGACCTCCCTCACGTC
ATCTCCGAGGCACCATCCTTTGCAGGTGTGCACCACATAGGTGGTGACATGTTCAAGAAGGTTCCCTCTGGGGAC
GCAATCCTCATGAAGTGGATACTCCACGACTGGAACGATGAGCATTGCACAACGCTGCTGAGCAACTGCTACGAT
GCACTACCCGCGCATGGCAAGGTGGTCATAGTGGAATACATCCTGCCAGTGAAGCCCGACGAAACGGCCACAGCA
CAGAGGTCCTTCGAAGCCGACATGATTATGCTCACACACACACCCGGCGGCAAGGAGAGGTACCTGAGGGAGTAC
GAAGAGCTCGCCAGGAGCGCCGGGTTTGCCAGCGTCAAGGCCACCTACGTCTACAGCAACATATGGGTCATTGAG
TTGAACAAATAG

>TaCPS-D2 (truncated)* (TraesCS2D02G029600; GenBank ON108665)

ATGATTAGCAAATCCCCATCCTATCCGGAGGTAGATGTGGGTGAATGGAAAGCTGATGAGTACAGACAACGCACG
GATGAACCGAGCGAGATGCGACAGATGATCGATGCCATAAGAACGGCCCTGGCGTCATTGGGTGATGATGAGACT
TCAATGAGTGTTTCAGCCTACGACACAGCGTTGGTCGCCCTTGTCAAGAACCTTGACGGAGGCGATGGACCGCAA
TTTCCCTCATGCATCGACTGGATTGTTCAAAATCAACTACCAGATGGTTCGTGGGGTGATCCGGCTTTCTTCATG
GTCCAAGATCGGATGATCAGCACCCTGGCATGTGTCGTGGCAGTGAAGTCTTGGAACATTGACAGCGACAACTTG
TGTGACAGGGGTGTGTTATTTATCAAAGAAAATATGAGCAGGTTGGTAGAAGAGGAACAGGACTGGATGCCATGC
GGCTTTGAGATTAACTTCCCAGCACTCCTAGAGAAGGCCAAGGACCTGGACTTGGACATCCCTTATGATGACCCT
GTTTTGGAAGAGATATATGCCAAGAGGAATCTGAAGCTCTCTAAGATACCTCTGGATGTGCTGCACGCCATACCA
ACGACCCTACTCTTCAGCGTCGAGGGAATGGTAGATTTACCATTGGACTGGGAAACGCTACTCAGATTGCGGTGT
CCGGATGGCTCCTTCCATTCCTCACCTGCTGCCACAGCTGCTGCCCTCAGTCACACCGGTGACAAGGAATGCCAT
GCGTTCCTGGATAGGCTCATCCAAAAGTTCGAGGGGGGAGTGCCATGTAGCCACTCAATGGACACTTTTGAGCAA
TTATGGGTGGTCGATCGGCTGATGCGTCTGGGGATATCAAGGCACTTCACAAGTGAAATTCAGCAGTGCTTAGAG
TTTATTTACAGGCGCTGGACTCAAAAGGGACTGGCTCACAACATGCACTGCCCAATCCCGGATATTGATGACACA
GCCATGGGTTTCCGTCTTCTCCGTCAGCATGGCTACGACGTCACTCCATCTGTGTTTAAGCACTTTGAGAAGGAT
GGCAAGTTCGTCTGCTTCCCAATGGAGACTAACCACGCTTCTGTTACCCCAATGCACAACACTTACCGTGCATCT
CAGTTCATGTTTCCTGGTGACGACGACGTCCTGGCTAGGGCCGGCCGCTATTGCCGTGAATTCCTTCAAGAGAGA
CAATCCTCCAACAAGCTGTACGACAAGTGGATCATCACCAAGGACCTGCCGGGCGAGGTCGGGTACACGCTAAAC
TTCCCATGGAAAGCAAGTTTGCCGCGAATTGAAACAAGGATGTATCTGGATCAGTACGGTGGCAACAACGATGTT
TGGATTGCCAAGGTCCTTTACAGAATGAATCTTGTGAGCAACGACCTGTACCTTAAAATGGCTAAGGCTGATTTC
AGAGAATATCAAAGACTCTCCCGAATTGAATGGAATGGCCTCAGAAAGTGGTATTTCAGGAACCATCTGCAGAGG
TATGGTGCGACTCCAAAGAGCGCGCTGAAGGCTTACTTCTTAGCTTCAGCAAATATCTTCGAACCCGGCCGAGCA
GCAGAACGTCTAGCATGGGCTCGCATGGCGGTGCTTGCTGAGGCGGTTACAACTCACTTCCGACACATTGGGGGT
CCATGCTATTCAACAGAGAATCTTGAAGAGCTTATCGACCTTGTTTCGTTTGATGATGTTTCCGGCGGCCTTCGT
GAAGCGTGGAAGCAGTGGCTTATGGCATGGACTGCAAAGGAGAGCCATGGCTCCGTTGATGGAGATACAGCATTG
TTGTTCGTCCGCACCATCGAGATCTGCTCAGGAAGGATTGTTTCAGCCGAGCAGAAACCGAACCTTTGGGATTAT
TCTCAGCTCGAGCAGCTCACCTCTTCCATCTGCCACAAGCTTGCCACAATAGGTCTTTCTCAGAATGAGGCAAGT
ATGGAGAACACAGAGGACTTACACCGGCAAGTGGATTTGGAGATGCAAGAACTATCTGGGCGTGTTCATCAGGGT
TGCCATGGCATCAGTAGAGAGACTAGACAGACATTTCTCAACGTGGTGAAAAGCTTCTATTACTCGGCTCATTGC
TCACATGAAACAGTTGATAGCCACATCGCAAAGGTCATATTTCAGGATGTCATTTAG

>TaKSL-D1 (truncated)* (TraesCS2D02G030100; GenBank ON108666)

ATGGCAATGTTGGCAGAAGAAACCATAGGACCGCGCAGTGATGTGGAACGGGATGCTAGAATACGAAAGCATCTC
AAAAACCCTGAACTCTCGCCGTCTGCGTATGACACGGCATGGGTGGCTATGGTGCCATTGCCGGACTTCGATCCG
CAGGCTCCATGCTTCCCTCAGTGTGTTGAATGGATATTGCAAAATCAACACTCTAGTGGGTCTTGGGGAATCAAT
GAATTTGGCTTATTAGCCAACAAGGATATTATGTTATCCACATTGGCCTGTATCATTGCACTTCATAAGTGGAAC
GTTGGCTCCGACCACATAAGGAGAGGATTAGAATTTATTGGAAGGAATTTCTCCACTGTCATGGATGATCAAATT
GTTTCTCCAGTAGGCTTCAATCTCATTTTCCCTGGTATGCTTAACCATGCTTTCGGGATGGGTTTGATAATTCCA
GTCACAGAAGCTGATATCAATGGGATACTTCACCTCCGTGAGATGGAGCTGACAAGATTAAGTGAGGAGAAATCG
TGTGGGAAAGATGCATATTTGGCCTATGTTGCTGAAGAAGGGTTAGTAAACCTGCTGGACTACAATCAAGTGATG
AAGTTCCAGCGAAAGAATGGGTCGTTGTTCAACTCTCCTGCCGCAACTGCTGCTGCATTAGTGCACTACTATGAT
AATAAAGCTCTCCAGTACCTCGACTCCATTGTCAGTATATTTGGTGGTGCAGTACCAACAGCGTACCCACAGAAT
ATATATTATCAGCTCTCAATGGTGGATATGCTCGAAAAGATCGGAATATCTCGCCATTTTTCGAGTGACACAAAC
AGCATCCTGGACAAGGCATACATTTCCTGGTTACAGAGAGACGAGGAGATCATGCAAGATGTAGAAACATGTGCA

ATGGCGTTTCGCCTTTTACGGATGAATGGTTATGATGTGTCGTCAGATGACTTGTCCCATGTTGCTGAAGCCTCC
ACTTTCCATAGCTCACTTGAAGGATATTTAAATCATACAAAATCTTTATTGGAGTTATACAAGGCTTCAAAAGTA
TGTTTGTCAGAAAATGAATTGATCCTGGAGAACATAAGCAACTGGTCAGGCCACTTATTGGCAGAGAAATTGCGC
TGTGATGGGACACAAAGAATGCCAATTTTTGGAGAGGTAGAATATACTCTTAAATTTCCCTTTTATGCGACAGTA
GAACCTCTAGACCATAAGAGGAACATTGAACATTTTGATTCTAGGGTTACTCAGCAGCTAAAGAGAAAAAACATG
CCATGTCATGTCAATCAAGATCTTCTAGATTTTGCCGTTGAAGATTTCAGTTTTTCTCAATCTATATACCAGGAT
GAACTCTGCCACCTCGAGAGTTGGGAGAAAGAAAACAGGCTGGAACAGCTTAAATTTCTACGCAAGGGGAGTCTG
ATAAATTGTTATCTCTCTGCTGCTGCCACCCTATCCACTCATGAACTCTCTGATGCTCGCATTGCATGTGCGAAA
ACTATTGCGCTCGTACTTGTTACTGATGACTTCTTTGATGTTGGAGCATCGAAAGAAGAACAAGAAAACCTCATA
GCATTAGTAGAGAAGTGGGATCACCATGACGAAGTTGAGTTCTGCTCTGAGCAAGTAGAAATAGTATTTTCTGCT
TTTTATAGTACAGTTAAGCACATTGGAGAAATGGCTTCCGCAGTGCAAAAGCGCGATGTTACAAAACACCTGAAT
GAAACATGGCTACATTACTTGAGGTCTGCAGCGACTGAGGCGGAATGGCAACGGAATCAATATGTGCCAACAGTT
GAGGAATACATGATAGAAGCGGTTAACTCATTCGCAGAGGGGCCCATTATGCTAACATCACTATATTTTGTCCAA
CAAAAACTCGAGGAGTACATAATCAAAGACCCGGAGTACCATGAGTTGCTTAGAATAAAGGGCAACTGTGGCCGT
CTCCTGAATGATACTAGGGGCTTCGAGAGGGAGTCCAGTGAGGGAAAACTGAACATCATCTCACTGCTTGTTCTT
CAGAGTGGAGGTTCCATGTCCATAGAAGCTGCTCAAGAGGCGGTACAGGAGTCTATAGCCTCATGTCGGAGAGAC
CTGCTAAGGATGGTTGTTAGAGAAGACCGTGTAGTTCCTAGGGTATGCAAGGAGGTGTTCTGGAGGTTTTGCAGG
ACAGTTCACTTGTTCTACTGTCAGACTGACGGATTTTCCTCGCCCAAGGAAATGCTCCGCACGATGAACGCAATA
TTCCGAGAGCCACTCAAACTCCAACATACCAGTCCTTTGGATGCTCAGTCAGAACAATAA

>TcGGPPS (truncated)* (GenBank AF081514)

ATGGCTTCCTATCAAGAATGCAATAGTATGAGGAGTTGTTTTAAATTGACACCTTTTAAAAGTTTTCATGGAGTG
AATTTCAATGTTCCCTCACTGGGTGCTGCTAATTGTGAGATTATGGGTCACCTGAAACTTGGGTCATTGCCATAT
AAACAATGTTCGGTGTCATCTAAATCCACAAAAACAATGGCCCAGTTGGTTGATTTGGCTGAAACAGAGAAGGCG
GAGGGAAAGGATATTGAATTTGATTTCAACGAGTATATGAAGTCCAAGGCTGTGGCAGTGGATGCGGCACTGGAT
AAGGCAATCCCACTTGAATATCCTGAAAAAATACATGAATCAATGAGGTATTCACTTCTAGCAGGAGGTAAGCGC
GTCAGGCCTGCTCTGTGCATTGCAGCATGTGAGCTTGTAGGAGGGAGTCAGGACCTTGCCATGCCAACTGCCTGT
GCAATGGAGATGATTCATACCATGTCTCTGATTCATGATGACTTGCCGTGCATGGATAATGATGATTTCAGAAGA
GGGAAGCCAACAAATCACAAGGTCTTTGGAGAGGACACTGCTGTTCTTGCAGGGGACGCCCTGCTTTCATTTGCA
TTTGAGCATATTGCTGTGGCTACAAGCAAGACTGTGCCTAGTGATAGGACTTTAAGGGTGATATCTGAATTGGGT
AAGACAATAGGCTCTCAAGGGCTTGTAGGGGGGCAGGTGGTTGATATTACATCCGAGGGGGATGCTAATGTGGAC
CTGAAAACCCTGGAATGGATTCATATACACAAGACTGCTGTGCTCTTGGAATGTTCAGTTGTGAGTGGAGGGATC
CTTGGTGGTGCTACAGAGGACGAGATTGCGAGAATTCGGCGGTACGCCCGGTGTGTGGGGCTTCTGTTTCAGGTT
GTGGATGACATACTTGATGTCACTAAATCTTCTGAAGAATTGGGAAAGACTGCAGGAAAGGATTTGCTTACTGAT
AAGGCTACTTATCCCAAGTTGATGGGCCTGGAGAAAGCAAAAGAATTTGCCGCTGAATTGGCGACGAGAGCCAAG
GAAGAGCTGTCATCCTTTGATCAGATAAAGGCTGCACCTTTGTTGGGTCTTGCAGATTACATTGCATTCAGGCAA
AACTGA

>TaOSC(5D)* (TraesCS5D02G011800; GenBank ON108667)

ATGTGGAGGCTAAAGATCGCGGAGGGCAGCGGCAACCCACTGCTGCGCACCACCAACGGCCACGTCGGCCGGCAG
GTATGGGAGTTCGATCCTGCCGCGGATGACCCCGACGAGGTCGCCGCCGTTGATGCCGCCCGCCGCGACTTCACC
AGCCGCCGGCACCAGATGAAGAACAGCTCCGACCTCCTCATGCGCATGCAGCTCTCTAAGTCAAACAATCTCAAG
ATGAACCTTCCTCCCATCAAGCTGGAACAACATGGGTATCACACAGAAGAAGACGATGTCTTGATATCTTTGAGG
AGGGCAATCAACCGATGTTCCACTCTCCAGGCACATGATGGCCATTGGCCGGGGGATTACGCTGGCACTTTATTT
CTCTTGCCCAGCTTGATTGTAGCACTGCATGTGACTGGAGCACTAACTACTGTGTTGACATCTGAACATCAGAAA
GAGATTCGGCGGTATCTCTACAATCATCAGAATGAAGACGGGGGTTGGGGACTGCATATCGAGGGAACAAGCACA
ATGTTCTGTACTGTCATGAACTATGTTGCTTTGAGATTGCTTGGGGAGGGGCTGGATTCATGCGGAGCCATGCTA
CAAGCTCGGAGTTGGATCTTGGATCATGGAGGAGCAACTTTAACACCATCGTGGGGAAAATTCTTTCTTTCGGTC
CTTGGGGTATATGACTGGTCCGGGAACAACCCATTGCCACCAGAACTATGGATGATGCCTTATTTCTTGCCTATT
CATCCAGGGCGCATGTGGTGTAATTGTCGGTTGGTATATTTGCCCATGTCATACCTATACGGGAAGAGGTTTGTG
GGTGCTACTACATCGATTGTACTAGACTTGAGGAAAGAGCTCTACAATGTCCCGTATAATGAGATTGACTGGGAC
AAGGCTCGCAATGGGTGTGCAAAGGAAGATCTCTATTATCCCCATTCACCACTACAGAATATTGTATGGGCCACT
CTCAAGAAAATTGGTGAACCGGTTCTTATGCACTGGCCTGGCAGCAATTTGCGAAAGAAAGCTCTGGACATTGTC
ATGAAACATATAAAGTATGAGGATGAAACAACTCAATATATATGCATTGCTCCTCTCAATAAGATGTTGAATATG
ATTTGTTGCTGGGTAGAAGATCCAAAGTCAGAGGCATTCAAACTCCATATTGAAAGAGTATATGATTTCTTATGG
GTTGCAGAAGACGGCATGAAGATGAAGTCTTACAATGGCAGCCAGCTATGGGATACAGCTTTGACAGTTCAGGCT
ATATTTGCTACCGGCCTCACAAAAGAGTTTGGCCATACAATTAAAGTCGCTCATGACTATATCAAACATTCCCAG
ATTCGTGCGGACTGCCCTGGAGACCAAAGCAAGTGGTATCGCCATCTATCAAAAGGTGGATGGACACATTCAACT
GCTGACCAAGGGTGGCCTGTATCAGATTGTACTGCAGAAGCATTTAAGGTTTTGTTGTTGTTAACTAAGGTTCCT
GAACTGGTTGATGAGCCTATAGAAACAAGCAGACTTGATGATGCAGTCAACATTCTACTGTCTTTGATGAATGAC

GATGGCAGTTTTGGTGCATATGAGCTAACAAGATCTTATGAATGGCTTGAGTTGTTGAATCCTTCTGAGAGCTTT
GGCGGCATAATGATCGAGTATCCGTATGTTGAGTGTACATCATCAGTAATTCAGGGTCTGGTGTTATTTAGAGAA
ATGTACCCCAAACACATTCGCTTGGAAGAGATAGACAGTTGTATCCAAAAGGCTGCAGACTACATTGAGAGCATC
CAATGGAGTGATGGATCATGGTATGGTTGTTGGGCTATCTGTTTCACATATGGTACTTGGTTTGGGGTGAGAGGA
TTGGTTGCTGCAGGGAGGACGTATGTAAATAGCCTAGCAATCAGAAAAGCGTGTGAATTTCTTTTGTCCAAGGAG
CTTCTCCCATCTGGAGGGTGGGGAGAAAGTTACCTTTCCAGTCAAGACAAGGTTTACACCAATCTTGAAGGAAAC
AGGGCTCATGCAGTTAACACTAGTTGGGCCATGTTGGCCCTAATTGATGCCGGGCAGGGTCAAAGAGATCCTGCA
TGTCTACATAGAGCAGCCAAGATTCTGATGAACTTCCAATCAGAGGATGGAGAGTTCCCTCAGCAAGACATTATT
GGAGCTACCAACCACAACCTTATGCTCACTTATGCCCAGTTCAGGAACATATTCCCTATCTGGGCTCTTGGAGAG
TACTACCAGAGAGTGCTTCCGGTAGCTTAA

>TaHSD(5D)* (TraesCS5D02G011900; GenBank ON108668)

ATGGATATGTGCCCGGTTGGCGGCTGCGACCGCCGGGAACCACGTCGTTTGTGCGCGGTGACCGGTGGGCGGGGA
TTCATGGCGAGTCACCTGGTGACGGCGCTGCTCGGCTCGGGAGATTGGTGTGTGCGGATCACCGACCTTGGCCCC
CAGGCTGCCCTGTCTCCTGCCGAGAGTGATGGACTCCTGGGCGCTGCCCTCCGCGATGGCCGCGCCGCCTACTTC
TCTGTCGACGTCTGCGAATTAGCCCAGCTTACAAAAGCTTTGGAAGGGGTAGATACTGTTTTCCACACTGCTGCG
GCGGATCATACCAACAACAACTTCCAACTTCATTACAAGGTTAATGTCGAGGGTACAAGGAATGTCATCGAGGCT
TGTAACACATGCAAGGTTAAAACACTCATATATACTAGTTCCAGTGGAGTTGTATTCGATGGAGTTCATGGCCTC
TTTGGCGTAGACGAATCTACACCGTATCCAGATAAGTTTCCCGACGCATACACAGAGACAAAGGCAGAAGCAGAA
AAGATGGTGATAAAGTCCAACGGAAGAAATGAGCTTCTCACTTGCTGTATACGTCCTGGCAGCATTTTTGGTCCT
GGAGACACGATAGTGCCAATTTTAGTATCTTATGGAGGAATGATGATCATTGTTGGTGATGGCAAGAATTGTGAT
GATTTTGTATATGTTGAGAATGTAGCGCATGGTCATGTATGTGCTGAGAAAACTCTTTCTACCATAGACGGTGCA
AAGAGAAGTGGAGGCAAAGCCTATTTTATAACAAATATGGAGCCTGTAAATATGTGGGACTTTGTTTATATGATT
TTGGAAGAACTTGGATACAAAAGCCGATTCAGATTAAGAATACCTTCATATTTTCTCAAGCCAATAACATATCTG
GTAGATTGGAGCTACCATAATATATTCTCTCACTATGGAATGCGTCAACCTGGCATGCTAACTTCCGCAAGCATT
AAGTATGCGACGCTGAATAGAACATTCAACTGCAACAATGCTGCTCAACAACTTGGTTACAAACCAATAGTGTCA
CTCAAGGAGGGAGTAAAGATGACTACTGATTTCTACAGGCGATTAAGAGCATGA

>TaCYP51H35 (TraesCS5D02G012300; GenBank ON108669)

ATGGACTTAGCAAGTCTCACCGCAGTGTGGTGGGCTGTGGCTCTTCTTTTCATCACCGTGTTAGTCACCAAGATT
TCAAGAGCAAGAGTCACCACCGTTGATCTACATCGTACAACAGGTCAACTTCCTCCCCTGGTGAATGGAGTTGCT
CTCCTAAAACTATTACCTACCCTTTTGAACAAGGGTCTACCGGCAATGGTGAATGATCTATATGTCAAATATGGC
AGTGTATTCATGGTAAGTTCATTTGGAGTGAAGGTAACACTCTTGATCGGGCCAGAGGTGACGGCTCATTTCTTC
CAAGGTCTGGAGTCGGAGATTAGCCACGGTAATCTGTTTGAGTTCACTGTGCCCATGTTTGGTGAAGCAGTGGGT
TATGGCAGAGATACCGCCACCCGAACTGAACAAATGCGCTTCCATATCGAAGCACTGAGACCATCAAGGTTGAGG
AGCCATGTTTATCCCATGCTTCAAGAAGTGGAGGGTTACTTTGCAAAATGGGGAGAGGAAGGCATTGTTGATCTA
AAGCTTGAGTTCGAGAAGTTACTCATGTTGATCTCAAGCAGATGTTTGCTCGGAAAAGAGGTTCGGGAGAACATG
TTTGATGAAGTCTACACACTTTTTCACGAGATTGAAGATAATGGTGTGACCTTGATTAGCTTCTTGTTCCCATAT
CTCCCAACTCCAGCAAATCGGAAGCGAGATAAAGCGCGCATCAGGCTAACACAAATCCTTTCTGATGTCGTCGAT
TCCCGTAAGAACTCCGGCAGAGTTGAGGATGATACATTGCAGAAATTGATCGACTCTAAGTACAAAGATGGTCGC
CCTACAACGGTAGAAGAGGTAGTCGGGCTAATCATTGGCCTGTTGTTTGCTGGAAAACACACCAGCTCTCACACT
AGTACTTGGACTGCAGCTTGTTTACTCAGCCATCCAACTTTCTTGAGAGCTGCCATTGAGGAGCAACAACAAATC
AGTAGCAAATACAAGGACATGGGGCTAGACTACAATGCATTTATAGAGATGGATACACTACATAGTTGCATCAAG
GAGGCGCTAAGGAAGCACCCTCCAACACCAATGCTGGTCCGCAGGGCACATAAGCAATTCATGGTGAAGACGAAA
GAGGGCAAAGAATATGACATCCCGCAAGACCACATCGTAGCAACTCCTACTATAGTGACTAATAACATCTCTTAT
ATCTATAAGGACCCTCAGGTATATGATCCATGCCGGTTTGGCCCCGAAAGAAGAGAGGACAAAGTTGGTGGCAAG
TTCTCTTACACGTCATTTAGTGGTGGAAGACACATTTGCACCGGGGAGGCCTATGCTTACATGCAACTTAAAGTG
ATATGGAGCCATTTGCTGAGGAACTTTGAGCTCGAATTGATCTCCCCTTTCCCCAAGACAGACTGGGGCAAGTTC
TTGCCAGAGCCACAAGGAAAACTACTCGTAAAATATAAGAGGAATGGCATTTTGTAG

>TaCYP51H37 (TraesCS5D02G012000; GenBank ON108670)

ATGGAGATGGCAAGTAGCGCCAAGTGGTTTGCTGTGGCTCTTGTTTTCATCACTGTGATCCTACTCAGGGTCATA
AGAGGAAGGAGGATGGCCGCTGCTCCAGCCAGTGAGAAACCACCTCCACCTGTGGTGAATGGTTTTGCGTTACTA
GGACTTCTACCTAGGCTTCTTACAATGGACCTTCGAACTAAGATAAATTGCCTGCACGATAAGTATGGCAGTGTG
TTCACAGTTAGTCCTTTTGGACTAACTAATGTAACCTTTCTGATCGGCCCGGAGGTCCAATCCCATTTCTTTCAA
GGGTTGGAGTCAGAGATTAACCATGGCAATCTTCTTGAGTTCCTTGTGCCGATGTTTGGACAAGAGATAGGTCAA
GGTGTCGATGCAGCCACTCGGACTGAGCAGTCCCGCTTTTATCTCGATGCATTAAAGCAATCCAAGTTGAGGAGA
CATCTTGATCCGATGCTTCAAGAGGTCGAGAGCTACTTCGGCAAATGGGGCCTGGAAGGAGTAGTTGATTTAAAA

CATGAGTTTGAAGAGTTGCTCATGTTGATATCAAGCCGGTGTCTACTAGGAAAAGAGGTTCGGGAGAAGATGTTT
GATGAGTTCTATAAACTTTTTCGTGATGTCGAAAATGGAGTGAACATGATCAGTGTCTTCTTCCCATATATTCCA
ATTCCTGCAAACCGTAGGCGTGACAAAGCACGTCTTAAGCTCATCGAATTACTTTCTGGAACTGTGAGGTCACGT
AAGAGCTCCCCCTCAGTGGAGGAAGATGTGCTACAAAGATTGATAGATTCCAAGTATAAAGACGGCCGCTCCACA
ACGGAAACAGAGGTAACCGGGATGATCATTGCATTGATCTTTGGTGGAAAGCACACAAGTTCCCTCGCTAGTACC
TGGACCGGAGCTTGCCTACTCACTCATCCAAAGTTCTTAGCGGCTGCATCCCAGGAGCAAAAGGAAATCATGATG
AAATACAAGGACAAAATAGAATATGATGCCTTGTTAGAGATGAATACCCTTCATAATTGTATCAAAGAGGCACTT
AGGCTGAACCCACCAACAACAATGTTGGTTCGCAAGGCACTTAAACATTTCACAGTGCGGACAAGACAAGGCCAA
GAGTATGGCATTCCCAAGGGGCATACTCTTGCAAGTCCCATAATACAAAACAATTACTTGCCTTACATTTATAAG
GACCCCGAGGTATATGATCCAGATCGGTTTGGTCCCGTAAGGCAGGAGGACATAGTTGGCGGCAAGTTTTCTTAT
ACATCCTTTGGTGGTGGAAGGCATTTTTGCAGTGGGGATGCCTATGCTTACATGCAAGTTAAAGTTATATGGAGC
CACTTGCTCAACAACTTTGATCTCAAATTATTATCTCCTTATCCCAAGACTGACTGGAGTAAGTTAATCCCAGAG
CCTCAAGGGAATATGATGGTCAGCTACAAGAGACGCCAGCTGCTAGGCTAG

>TaCYP51H13_5A (TraesCS5A02G004600; GenBank ON108671)

ATGGACTTGACAAGTCTCACTACGATGTGGTGGGCCATAGCTCTTCTTTCCATTACTGTATTGGCCACCAAGATC
ACAAGAGCAAGAATCAAAAACATTGATCCACAGCGTACAACAGGCCAACTTCCACCCATGGTGAATGGACTTGCT
CTCCTAGGATTATTACCTACCCTTCTGAAGAAGGGTCTACCACCTATGTTCAATTATCTATATGTTAACTATGGC
AGTGTCTTCACTGTAAGTTGCTTTGGAGTGATCAAGGTAACACTCTTGATCGGGCCAGAGGCGACGACTCATTTC
TTCCAAGGTTTGGAGTCAGAGATTAGCCATGGTAATCTGCTTGAGTTCACAGTACCCATGTTTGGCAAGGCAGTT
GGTTATGGTAGAGATACCGCCACCCGAATGGAACAAATGCGCTTCCACAGTGAAGCACTGAGGGCATCCAGGTTA
AGGAGCCATGTTTCTCCAATGCTTCAAGAAGTGGAGGATTTCTTTGCAAAGTGGGGAGAGGAAGGCGTAGTTGAT
CTAAAGCTTGAGTTCGAGCAGCTACTTATGTTGATATCAGGCCGATGTTTGCTTGGAAAAGAGGTCCGGGAGAAT
ATGTTTGATGAAGTCTACACACTTTTTCGCGAGATCGAAAAGGGTGTGACCTTGATCAGCTTCTTGTTCCCATAT
CTCCCAACTCCAGCAAACCGGCAGCGTGATAGGGCACGTATAAGGCTAACAGAGATCCTCTCTAATGTTGTCGAG
TCCCGTAAGAGCTCCGGGCGAGTGGAGGAGGACACGCTGCAGAAACTGATTGACTCCAAGTACAAAGATGGTCGC
CCTACAACAGTAGAAGAGGTAGCCGGGTTGATCATTGGCTTGCTGTTTGCTGGAAAACACACCAGCTCTCATACT
AGTACTTGGACTGGAGCTTGTCTTCTCAGCCATCCAGCCTTCTTAAGAGCTGCCATTGAGGAGCAACATCAAGTC
GCCAAGAAATACAAGGACGGACCAGAATACAATGCCTTCTTAGAGATGGATACGCTGCATAATTGCATCAAGGAG
GCGCTGCGGAAGAACCCTGCAACACCACTGCTGGTTCGCAGGGTCCATAAGCGCTTCACGGTGCAGACGAAAGAA
GGCCAACAATATGACATCCCGCAGGACTACATCGTAGCAACTCCTACTATAGTAAACAATTATATCCCTTACATT
TATACGGACCCTCAGGTGTATGATCCAAATCGGTTTGGCCCAGAAAGAAGAGAGGACAAAGTGGGTGGCAAGTTC
TCTTACACATCATTTGGTGGTGGAAGGCACGTTTGCACTGGAGAGGCTTATGCTTACATGCAACTCAAAGTGATA
TGGAGCCATTTGCTGAGGAACTTTGATCTCGAATTGGTCTCTCCCTTCCCTAACACAGACTGGAGCAAGTTCTTG
CCAGATCCAAAGGAAAAATTACTCGTGAGATATACGAGAAATGGAATTTAA

>TaCYP51H13P (TraesCS5D02G012100/200; GenBank ON108672)

ATGGACTTGACAAGTCTCACTGCCATGTGGTGGGCCATAGCTCTTCTTTTCATTACTGTATTGGCCACCAAGATC
ACAAGAGCAAGAATCACCAACATTGATCCACAGCGTACAACAGGCCAACTTCCACCCATGGTGAATGGACTTGCT
CTCCTAGGATTATTACCTACCCTTCTGAAGAAGGGTCTACCACCTATGGTCAATTATCTATATGTTAACTATGGC
AGTGTCTTCACTGTAAGTTGCTTTGGAGTGATCAAGGTAACACTCTTGATCGGGCCAGAGGCGACGACTCATTTC
TTCCAAGGTTTGGAGTCAGAGATTAGCCATGGTAATCTGCTTGAGTTCACAGTGCCCATGTTTGGCAAGGCAGTT
GGTTATGGTAGAGATACCGCCACCCGAATGGAACAAATGCGCTTCCACAGTGAAGCACTGAGGGCATCCAGGTTA
AGGAGCCATGTTTCTCCAATGCTTCAAGAAGTGGAGGATTTCTTTGCAAAGTGGGGAGAGGAAGGCGTAGTTGAT
CTAAAGCTTGAGTTCTAGCAGCTACTTATGTTGATATCAGGCTGATGTTTGCTTGGAAAAGAGGTCCGGGAGAAT
ATGTTTGATGAAGTCTACACACTTTTTCGCGAGATCGAAAAGGGTGTGACCTTGATCAGCTTCTTGTTCCCATAT
CTCCCAACTCCAGCAAACTGGCAGCGTGATAGGGCGCGTATAAGGCTAACAGAGATCCTCTCTAATGTTGTCGAA
TCCCGTAAGAGCTCCGGGCGAGTGGAGGAGGACACGCTGCAGAAACTGATTGACTCCAAGTACAAAGATGGTCGC
CCTACAACAGTAGAAGAGGTAGTCGGGTTGATCATTGGCTTGCTGTTTGCTGGAAAACACACCAGCTCTCATACT
AGTACTTGGACTGGAGCTTGTCTTCTCAGCCATCCAGCCTTCTTAAGAGCTGCCATTGAGGAGCAACATCAAGTC
GCCAAGAAATACAAGGACGGACTAGAATACAATGCCTTCTTAGAGATGGATACGCTGCATAATTGCATCAAGGAG
GCGCTGCGGAAGAACCCTGCGACACCACTGCTGGTTCGCAGGGTCCATAAGCGCTTCACGGTGCAGACGAAAGAA
GGCCAACAATATGACATCCAGCAGGACTACATCGTAGCAACTCCTACTATAGTAAACAACAATATCCCTTACATT
TATACGGACCCTCAGGTGTATGATCCAAATCGGTTTGGCCCCGAAAGAAGAGAGGACAAAGTGGGTGGCAAGTTC
TCTTACACATCATTTGGTGGTGGAAGGCACGTTTGCACCGGAGAGGCTTATGCTTACATGCAACTCAAAGTGATA
TGGAGCCATTTGCTGAGGAACTTTGATCTCGAATTGGTCTCTCCCTTCCCTAACACAGACTGGAGCAAGTTCTTG
CCAGATCCACAGGGAAAATTACTCGTGAGATATACGAGAAATGGAATTTAA

>AtCYP51H35 (AET5Gv20012700; GenBank ON108673)

ATGGACTTAGCAAGTCTCACCGCAGTGTGGTGGGCTGTGGCTCTTCTTTTCATCACCGTGTTAGTCACCAAGATT
TCAAGAGCAAGAGTCACCACCGTTGATCTACATCGTACAACAGGTCAACTTCCTCCCCTGGTGAATGGAGTTGCT
CTCCTAAAACTATTACCTACCCTTTTGAACAAGGGTCTACCGGCAATGGTGAATGATCTATATGTCAAATATGGC
AGTGTATTCATGGTAAGTTCATTTGGAGTGAAGGTAACACTCTTGATCGGGCCAGAGGTGACGGCTCATTTCTTC
CAAGGTCTGGAGTCGGAGATTAGCCACGGTAATCTGTTTGAGTTCACTGTGCCCATGTTTGGTGAAGCAGTGGGT
TATGGCAGAGATACCGCCACCCGAACTGAACAAATGCGCTTCCATATCGAAGCACTGAGACCATCAAGGTTGAGG
AGCCATGTTTATCCCATGCTTCAAGAAGTGGAGGGTTACTTTGCAAAATGGGGAGAGGAAGGCATTGTTGATCTA
AAGCTTGAGTTCGAGAAGTTACTCATGTTGATCTCAAGCAGATGTTTGCTCGGAAAAGAGGTTCGGGAGAACATG
TTTGATGAAGTCTACACACTTTTTCACGAGATTGAAGATAATGGTGTGACCTTGATTAGCTTCTTGTTCCCATAT
CTCCCAACTCCAGCAAACCGGAAGCGAGATAAAGCACGCATCAGGCTAACACAAATCCTTTCTGATGTCGTCGAT
TCCCGTAAGAACTCCGGCAGAGTTGAGGATGATACATTGCAGAAATTGATCGACTCTAAGTACAAAGATGGTCGC
CCTACAACGGTAGAAGAGGTAGTCGGGCTAATCATTGGCCTGTTGTTTGCTGGAAAACACACCAGCTCTCACACT
AGTACTTGGACTGCAGCTTGTTTACTCAGCCATCCAACTTTCTTGAGAGCTGCCATTGAGGAGCAACAACAAATC
AGTAGCAAATACAAGGACATGGGGCTAGACTACAATGCATTTATAGAGATGGATACACTACATAGATGCATCAAG
GAGGCGCTAAGGAAGCATCCTCCAACACCAATGCTGGTCCGCAGGGCACATAAGCAATTCATGGTGAAGACGAAA
GAGGGCAAAGAATATGACATCCCGCAAGACCACATCGTAGCAACTCCTACTATAGTGACTAATAACATCTCTTAT
ATCTATAAGGACCCTCAGGTATATGATCCATGCCGGTTTGGCCCCGAAAGAAGAGAGGACAAAGTTGGTGGCAAG
TTCTCTTACACGTCATTTAGTGGTGGAAGACACATTTGCACCGGGGAGGCCTATGCTTACATGCAACTTAAAGTG
ATATGGAGCCATTTGCTGAGGAACTTTGAGCTCGAATTGATCTCCCCTTTCCCCAAGACAGACTGGAGCAAGTTC
TTGCCAGAGCCACAAGGAAAACTACTCGTAAAATATAAGAGGAATGGCATTTTGTAG

>AsOSC1* (AS01G014480; GenBank ON108674)

ATGTGGAGGCTGAAGATCGCGGAGGGCGGCGGCAACCCGCTGCTGCGCACCACCAACGGCCACGTTGGGCGGCAG
GTGTGGGAGTTCGATCCTGCCGCCTCCGACGATGACCCCGACGAGATCGCGGCCGTAGACGCCGCGCGCCGCGAG
TTCACCGGCCGCCGCCATCAGATTAAGAACAGCTCCGACCTCCTCATGCGCATGCAGTTCTCCAAGACAAACGCT
CTCAAGATTAACCTTCCTCATGTCAAGCTGGCAGAGCATGAATATGCAACAGAAGAGGATGTCCTCATATCTTTG
AAGAGGGCAATTAGCCGCTATTCCACTCTCCAGGCACATGATGGGCACTGGCCGGGGGATTATGCAGGCACTTTA
TTTCTCTTGCCCAGCTTGATTGTAGCATTGCACGTGACTGGCGCACTGACTACAGTCTTGACATCTGAACATCAG
AAAGAGATCCGGCGATATCTCTACAATCATCAGAATGAAGATGGAGGCTGGGGACTGCACATTGAGGGAACAAGC
ACAATGTTCTGTTCTGTCATGAACTATGTTGCTTTGAGATTGCTTGGGGAAGGGCTGGATTCAGGTGGAGCCATG
CTGCAAGCTCGTAGTTGGATCTTGGACCATGGAGGAGCAACTTTAACACCATCTTGGGGCAAATTCTTTCTATCG
GTCCTCGGGGTATATGACTGGTCTGGAAACAACCCATTGCCACCAGAAATATGGATGCTTCCCTATTTCTTGCCT
ATTCATCCAGGACGTATGTGGTGTAATTGCCGACTAGTATATTTGCCAATGTCGTACTTATATGGAAGAGGTTT
GTGGGTTCTATGACATCAATTGTCCTAGACTTGAGGAAAGAGCTGTACAATGTCCCATACGATGAGATTGACTGG
GACAAGGCTCGCAATGGATGTGCCAAGGAAGATCTCTATTATCCCCATTCACCGCTGCAGGATATCGTGTGGGCT
ACTCTCAAGAAAATTGGTGAACCAGTTCTCATGCACTGGCCTGGCAGCAAATTGCGGGAGAAAGCTCTGGACATT
GTGATGAAACATATTGAGTACGAGGATGAAACAACACAATATATATGCATTGCTCCTATGAATAAGATGTTGAAT
ATGATTTGCCGTTGGGTAGAAGATCCGAACTCAGTGGCATTCAAACTCCATATTGAAAGAGTATATGATTTCTTA
TGGGTCGCTGAAGACGGCATGAAGATGAAGTCTTATAATGGCAGCCAGCTGTGGGATACAGCTTTGACTGTTCAA
GCTATATTTGCTGCCGGACTCACAGAAGAGTTTGGCCAGACAGTTAAACTTGCGCATGACTATATCAAGCGTTCC
CAGATTCGTATGGACTGTCCTGGAGATCAAAGTAAATGGTATCGTCATATATCTAAAGGTGGATGGACACATTCA
ACAGCTGACGAAGGGTGGCCTGTATCAGACTGTACTGCGGAAGCACTGAAGGTTTTATTGCTGTTAACTAAGTTT
CCTCCTGAATTGGTTGGTGAATCTATAGAAGCAAGCAGGCTTGATGATACAGTCAACATTTTGCTGTCTTTGATG
AACGACGATGGTAGTTTTGGTGCATATGAGCTAACAAGATCTTATGAATGGCTTGAGTTGCTCAATCCTTCTGAG
AGCTTTGGGGGCATAATGATCGAGTATCCATATGTTGAGTGTACGTCATCAGTAATTCAAGGTCTTGTGTTATTC
AGAGAAACCTACCGTAAACATTATCGCAGGGAAGAGATAGACCATTGTATCCGAAAGGCTGCAGACTACATTGAG
AGCATCCAACGAAGTGATGGATCATGGTATGGCTGTTGGGCAATTTGTTTCACCTATGGTACATGGTTCGGGGTG
AGAGGATTGGTTGCTGCCGGTAGGACGTACAAAAACAGCCAAGCAATCAGAAAAGCGTGTGAATTTCTGCTATCT
AAGGAGCTTCTTCCGTCAGGAGGATGGGGAGAAAGCTACCTCTCTAGTCAAGACAAGGTTTACACCAATCTTGAA
GGCAGCAAGGCTCATGCAGTTAACACCAGTTGGGCCATGTTGGCCCTGATTGATGCTGGGCAGGGTAAAAGGGAC
CCTGCATGTCTACATAGAGCAGCCAAGGTTCTGATCAATTTCCAATCCGTGGATGGAGAGTTCCCTCAGCAAGAC
ATTATCGGAGCTACCAACCACAACCTCATGCTCACTTACGCCCAGTTCAGGAACATCTTCCCTATCTGGGCTCTT
GGGGAGTATTATCGCAGAGTGTTGATAGCACAAAGGCTCTAA

>CYP51H73* (AS01_002578_0375348; GenBank ON108675)

ATGGACATGACAAGTGGCGCCCTGTGGTTCACCTTGGCTCTTGTTTTCATCACTCTCCTACTGAGGCTTATACAA
CGAAGGATTGCCGTTGCTCCAACCAGTAGAAACCCACTTCCACCAGTGGTGAATTGTTTTGCTTTGCTGCGAATT
TTACCTAGCCTTTTTGGAAAGGATCTTCCAACGAAGATAAATTTTCTATATAAGAAGTATGGCAGTGTGTTCACA
ATTAGTCTATTTGGACGTCATGTAACCATTCTGATCGGACCGGAGGTATCAGCTCATTTCTTTCAAGGGTTGGAT
TCAGATATTAGCCATGGCAACTTTCTTGAGTTTACTGTACCCATGTTTGGGCAAGAGGTTGCTTATGGCGTTGAT

ACGGCAACTCGGAATGAGCAGTCTCGTTTCTATCTTGAAGCATTAAGGCAATCAAAGTTGAGGGGACATTTCCAT
CCCATGCTTCAAGAGGTGGAGGAATACTTTGGCAAATGGGGCGAGGAAGGCATTGTTGATTTAAAACATGAATTT
GAAGAGATACTCATGTTGATCTCAAGCCGTTGTCTACTAGGAAAAGAGGTCCGGGAGAATATGTTTGAGGATTTC
TATGCACTGTTCCGTGATATCGAAAATGGAGTGAACTTGGCCAGTGTCCTTTTCCCATACATCCCAATTCCAGCA
AACCGCAGGCGTGACAAGGCACGTATGAAGCTAATAGAAATATTCTCTCAAATTATGAGATCACGTGAGAGCTCT
AGCAGGGTTGAGGAGGACGTGCTACAGAAATTGATGGATTCTAAGTATAAAGACGGGCGCTCCACAACCAAAACA
GAGGTAACAGGACTGATAATTGGGTTGATCTTTGCTGGAAAGCACACAACCTCTCAAGCTATAACCTGGACTGGG
GCTGACCTACTTAGCCATGAAAAATTCTTAGCAGCTGCATCCGAGGAGCAAAAGGAAATCATGATGAAATACAAG
GGCAAAATAGAATATGATGCCTTGCTAGAGATGAGTACCCTTCATAGGTGTATCAAAGAGGCACTTCGGATGCAC
CCACCAGGACCAATGTTGATTCGCAAGGCACATAAAAATTTCACGGTCCGAACAAAAGAAGGACAAGAGTATGAT
ATTCCGAAAGGGCATAGCATTGCAAGTCCTATAGTGCAAAACAATAGGATGCCTTACATTTATAAGGATCCTCAT
TTGTATGACCCAGATAGGTTTGGTCCGGCAAGACGAGAGGACGTAGTTGGCGGCAAGTTCTCTTACACATCTTTT
GGCGCTGGAAGGCATTCTTGCGTTGGGGAGGCCTATTCTTATGTGCAAATTAAAGTCATATGGAGCCATCTACTG
AACAACTTTGATCTCAAATTATTGTCCAGTTATCCTAAAACTAATTGGAATAAGCTAATCCCAGAGCCTCAAGGA
AGTATGATGGTCAGCTACAAGAGACGTCGGCTACCGGGCTAG

>BdOSC2 (Bradi3g22802; GenBank ON108676)

ATGTGGAAGCTAAAGATCGCAGAGGGGTGCGGCAACCCGCTGCTGCGCACCACCAACGATCACACTGGCAGGCAG
GTATGGGAGTTCGACCCTGACGACTCCGTCGACGAGTCTCCAGCCGTGGAAGCCGCCCGCCGCGAGTTCTCCAGC
CGCCGGAATCAGACCAAGAACAGTTCTGACCTCGTCATGCGCATGCAGTTCTCCAAGTCTAACCGTCTCAAGATG
AACCTTCCTGCCATCAAGCTGGATGAGCGTGCATTCGCCACGGAAGAGGATGTCTTGATATCTTTGAAGAGGGCA
ATCGGCCGCTATTCCGCACTCCAGGCACATGATGGGCACTGGCCGGGAGATTACGCAGGAACTCTGTTTCTCTTG
CCTAGCTTGATTGTAGCATTGCATGTGACTGAATCACTCAATACTGTGTTGTCATCTGAACATCAGAAAGAGATG
CGGAGATATCTCTACAATCATCAGAATGAAGATGGGGGCTGGGGACTGCACATTGAGGGAACAAGCACAATGTTC
TGTTCTGTCATGAACTACGTCGCCTTGAGATTGCTTGGGGAAGGGCTGGATTCGTGTGGAGCCATGCTGCAAGCT
AGGAGTTGGATCTTGGACCATGGGGGGCAACTTTAACACCATCTTGGGGGAAATTCTTCCTTTCGGTCCTTGGG
GTATATGAATGGTCTGGCAACAACCCATTGCCACCAGAAATATGGATGATGCCTTATTTCTTGCCTATTCATCCA
GGGCGCATGTGGTGTAATTGCCGCCTGGTATATTTGCCCATGTCATACCTATATGGAAAGAGGTTCGTGGGAGCT
ATTACATCATCAGTTGTACTAGACTTGAGGAAAGAACTTTACATTGTGCCATACGATGAGATTGACTGGGACAAG
GCTCGCAATGGCTGTGCAAAGGAAGATCTCTATTATCCTCATTCACCGCTGCAGGATGTGGTATGGGCTACTCTG
AAGAAAGTTGGCGAGCCACTTCTTATGCACTGGCCTGGCAGCAAATTGCGGCAGAGAGCTCTGGACGTTGTCATG
GAACATATCCGTTACGAGGATGAAACAACTCAATATATCTGCATTGCTCCTCTGAACAAGATGTTGAATATGATT
TGTCGCTGGGTAGAAGATCCAAACTCAGAGGCATTCAAGCTCCATATTGAAAGAGTCTACGATTTCTTATGGGTC
GCTGAAGACGGCATGAAAATGAAGTCATACAATGGCAGCCAGTTGTGGGATACGGCTTTAACAGTCCAGGCTATT
TTTGCTACCGGCCTCACAGAAGAGTTTGGCCCCACAATTAAACTAGCCCATGACTACATCAAACGTTCCCAGATT
CGTGTTGACTGTCCTGGAGACCAGAGCAAGTGGTATCGCCATATATCCAAAGGTGGCTGGACGCACTCAACTGCT
GACCAAGGGTGGCCTGTATCAGATTGTACTGCAGAAGCATTGAAGGTTCTGTTGCTGTTAACTAAGGTTCCTCCT
GAATTGGCTGGTGAGCCTATGGAAGCCAGCAGGCTTGATGATAGTGTCAACCTTCTGTTATCTCTGATGAACGAG
GATGGCAGTTTTGGTGCATATGAGCTGACAAGATCTTATGAGTGGCTTGAGTTGCTCAATCCTTCTGAGAGCTTT
GGGGGAATAATGATCGAGTATCCGTATGTTGAGTGTACATCATCAGTAATTCAGGGGCTGGTGTTATTCAGAGAA
ACATACCCTAAACACTGTCGCAGGGAAGAGATAGACAACTGTATCCGGAAGGCTGCAGATTACACAGAGAGCATC
CAACGTGCCGATGGATCATGGTACGGCTGTTGGGCTATCTGTTTCACCTATGGTACATGGTTTGGGGTGAGAGGA
TTGATTGCTGCCGGTAGGACTTACAAGAACAGCCAATCAATCAGAAAGGCGTGCGAATTTTTGCTGTCCAAGGAG
CTCCCATCAGGAGGATGGGGGGAAAGCTACCTCTCCAGTCAAGATAAGGTTTACACCAATCTTGAAGGCAACAAG
GCTCATGCAGTTAACACCAGTTGGGCCATGTTGGCCCTGATTGATGCTGGACAGGGTAAAAGAGATCCTGCATGT
CTGCATAGAGCAGCCAAGGTTCTGATCAACTTCCAAATGGAGGATGGAGAGTTCCCTCAGCAAGACATCATCGGA
GCTACCAACCACAACCTCATGCTCACTTACGCCCAGTTCAGGAACATCTTTCCGATCTGGGCTCTTGGAGAGTAC
CATCGCAGAGTTCTGCAAGCACAAAAGGCATAA

>BdCYP51H14 (Bradi3g22820; GenBank ON108677)

ATGTTCATGACAAGTAGCGCCATGTGGTTCACCGTGTCTCTTGTTTTCATCACCATCGTGCTCATCAGGATCATC
AGAGGGAAGGCAGCTGGTGGTGTTGATCCAACTTCTGGGAAACAACACCCGCCTGTGGTGAATGGCTTTGCTTTA
CTAGGACTTTTACCTAGGCTTTTGAACAAGGATCTTCCAACTAAGATAAATTGTCTATATAGTAAGTATGGTAGC
GTGTTCACGGTAAGTCTGTTTGGACGGAACGTAACCTTCTTGATCGGGCCTGAGGTCTCAGCTCATTTCTTTCAA
GGATTGGACTCAGAGATTAGCCATGGCAATCTTCTTGAGTTCACTGTCCCCATGTTTGGCCAAGAGGTCGCTCAT
GGTGTCGATACAGCCACTCGGAATGAGCAGGCCCGGTTCTATCTTGACGCATTAAAGCAATCAAAGTTGAGAAGC
CATTTTGATCCCATGCTTCAAGAGGTAGAGGAATACTTTAGCAAATGGGGGCAGGAAGGCATAGTTGATCTGAAG
CACGAATTTGAGCAGTTACTTATGTTGATCTCAAGCCGATGTCTACTCGGCAAAGAGGTCCGAGAGAAGATGTTT
GACGAGTTCTATACCCTGTTTCGTGACATTGAAAACGGTGTGAACTTGGTCAGTGTCTTCTTCCCATATATCCCA
ATCCCAGCAAACCGGAGACGCGACAGAGCACGCGTGAAGCTCATTGAGCTGCTCTCTGAAATCGTGAGGTCACGT

AAGAGCTCAGGCAGAGTGGAGGAGGACGCCCTGCAGAAACTGATAGATTCCAAGTACAAAGACGGCCGCTCCACA
AGCGAAACAGAAGTCACCGGGATGATAATTGGACTGATCTTTGGGGGAAAACACACAAGCTCTCATGCCACTACC
TGGACCGGAGCCTGCCTACTCAGCCATGAGAAGTTCTTAGCAGCCTCATCCGAAGAGCAAAAGCAAATCATGAAG
AAATACAAGGGGAGATTAGAATATGAAGCTCTGTTAGAGATGAATACACTCCATAGTTGCATCAAGGAGGCGCTT
CGGATGCACCCGCCATCGCCGATGCTGGTTCGCAAGGCAAATAAGCATTTCACTGTGAAGACAAGAGAGGGCCAG
GAGTATGATATTCCAAAAGGGAATACTATTGCAAGCCCTATAGTACAAAACAATATCATGCCATACATTTATAAG
GACCCTCACTTGTATGATCCAGATAGGTTTGGACCCGCGAGGCAGGAGGACGTAGCTGGTGGGAAATTCTCTTAC
ACGTCTTTTGGAGGTGGAAGGCATGTCTGCGTTGGCGAGGGCTATGCTTACATGCAGATTAAAATCATATGGAGC
CATTTGCTCAAGAACTTTGAGCTCGAGTTGCTGTCTCCTTTTCCCAACACCGATTGGAATAAGTTAATCCCAGAG
CCTCAAGGGAATATGATGGTTAGCTATAAGAGACATCGGCTGTTGGGCTAG

>BdCYP51H15 (Bradi3g22840; GenBank ON108678)

ATGGACTTGGCAAGCACAGCCGCCGTGGGATGGGCGCTCGCTCTTCTTCTCATCACTGCTTTAGCTACCAAGATA
GCAAGAGCAAGAATCACCATTGTCGATCCACCGCGTACGACGACGACGACGACTCAACCTCCACCCGTGGTGAAT
GGCATCGCTCTCCTAAGACTGTTACCCACCCTTTCCAAGAAGGGTCTGCCGGCCATGGTGAACGATCTGTATCTG
AAGTTCGGCAGCGTGTTCATGGTAAGCGTGTTTGGGATCAAGGTAACGATCTTGATCGGGCCAGAGGTGACCCCT
CACTTCTTCCAAGGTCTGGAGTCGGAGATTAGCCACGGTAATCTGCTTGAGTTCACCGTGCCCATGTTTGGCGAA
GCGGTTGGTTACGGCAGAGATGCCGCCACCCGAATTGAACAGACCCGCTTCCACATCGAGGCGCTGAGGCCATCC
AGGTTGAAGAACCACGTCTATCCCATGATTCAAGAGGTCGAGGACTATTTCGCAAAATGGGGAAACGAGGGCATA
GTGGACCTAAAGCTTGAGTTCGAGCGGTTACTCATGTTGATCTCAAGCCGAGTTTTACTCGGAAAAGAAGTTAGA
GAAACCATGTTTGATGAGGTCTACACACTGTTTCGTGAAATCGAAAACGGTGTGACCGTGATCAGTTTCTTATTT
CCATATCTCCCGATTCCAGCAAACCACCGGCGAGATAGAGCGCGCACCAGGCTCACAGAAATACTCTCCGATGTC
GTCAAGTCGCGTAAGAACTCAGGCAGGGTTGAGGAGGACACACTCCAAAAATTGATAGACTCCAAGTATAAAGAT
GGCCGCTCCACAACCGTACCAGAGGTTGTCGGGTTGATCATCGGTCTCCTATTTGCTGGAAAACATACCAGCTCT
CACACCAGCACCTGGACTGGAGCCTGTTTGCTTAGCCACCCAATGTTCTTAAGAGCTGCCATTGAGGAGCAAAAA
CAAATCGGTAGGAAATATAAGGATGGGCTCGGCTACGATGCTATGTTAGAGATGGATACCCTGCATAGTTGCATC
AAGGAGGCGCTAAGGATGCACCCTCCAACGCCAATGCTGGTTCGCAAGGCACATAAGCACTTTACGGTGCAGACG
AAACAAGGCAAACAATATGACATCCCAGAAGGGCACACCGTAGCGAGTCCTATTGTAGTCAACAATAATATCCCC
TATATTTATAAAGACCCAGAGGTTTATGATCCAGACCGGTTTGGTCCCAAAAGACAAGAGGACAAGGTAGGTGGC
AAGTTCTCTTATACGTCCTTCAGTGGTGGAAGGCACACTTGCACTGGTGAGGCCTATGCTTACATGCAAATTAAG
GTGATATGGAGTCATTTGCTAAGTAACTTTGAACTCAAATTGATCTCACCTTTCCCCAAGACAGATTGGAGCAAA
TTCTTACCAGAGCCAGAAGGAACATTGTTTGTAAGATATAAGAGAAATGGCTGCCGTGCTAGCACTTAG

>BdCYP51H16 (Bradi3g22850; GenBank ON108679)

ATGGAATTTACAAGTGGCGACGTGTGGTTCGCCGTAGCTGTTCTCTTAATTGCTACGGTCGTCACCAAGATTGCA
ATTGCAAGAGCTACCGTTGATCCAACGTGCACAAGACCACTTCCACCTGTGGTGAAGGGCGTTGCTCTCCTTGGA
CTTCTACACGCCCTTGTTACCAAGGATCTACCAACTGTGATACATGATCTGTATGAAAAATTTGGCAGTGTGTTC
ACAGTAAGCTTGCTTGGACAAAAGGTGACCTTCTTGGTCGGGCCAGAGGTGTCGGCCCATTTCTTCAAAGGGTTG
GAGTCAGAAATTAACATCGGCAATCTGCTGAATTTCACCGTGCCCATATTTGGTCAAGAGGTGGGTTACGGCGTG
GATCTCGCAACTCGGAATGAGCAGGCCCGATTCTGCCTTGATGCACTGAAGCCGTCCAAGTTGAGGAGCCATGTT
GACCCCATGCTTCAAGAAGTAGAGGAGTACTTTGCAAAGTGGGGACAACATGGGATAGTTGATTTAAAACATGAG
TTTGAGGTGTTACTCATGTTGATCTCAAGCCGGGTTCTACTCGGTAAAGAGGTCCGGGAGAAGATGTTCGATGAG
TTCTGCACATTATTTGGTCAGATCGAAAACGGGGTGAACCTTGTCAGTGTCTTCTTCCCATACATCCCAATTCCA
TCAAACCAGAGACGCGACAGAGCACGCGTCAAGCTCACACAGATACTATCTGAAGTTGTGAGTTCGCGCAAGAGC
TCCGGCCGAGTTGAAGAGGATACGCTGCAGAGACTCATCGATTCCAGGTATAAAGACGGCCGATCCACAACGGAA
GCAGAGATCACCGGGATGATAATTGGCATGCTCTTCGCTGGGAAACACACGAGCTCTCACACTACTATCTGGACT
GGAGCATGCCTCCTTAACAGTCCCAAATTCCTGGCTGCTGCTGTCGAGGAGCAAAAGGAACTCGTTGTGAAATAC
AAAGACCAGATCGACTACAATACCTTGGCAGAGATGGATGCCCTCCATTGCTGCATCAAAGAGGCACTCCGGATG
CACCCTCCGTCGCCCGTGCTGGTCCGCAAGGCACAGAAGCAGTTCACCGTGAAGACGAAAGAAGGTAACGAGTAT
GACATCCCAAGGGGGCATACCATTGCCAGCCCTACGATAGTTAACAACAATATGCATTACATTTATAAGGAGCCC
CAAGTGTATGATCCGGATCGTTTTGGCCCTGGAAGAGAGGAGGACATAGTTGGTGGCAAGTTCTCTTACACGTCA
TTTGGCAGCGGAAGACATGCTTGCATTGGCGAGTCATATGCTTACATGCAAATTAAATTGATATGGAGCCATTTG
CTGAGGAATTTTGAGCTTGAACTGGTTTCTCCTTTCCCGAGGACAGACTGGAACAAGATTGTGCCAGGACCTCAA
GGGAAGGTCATGGTAAGTTATAAGAGATCGAGGATGTCAGCCTAG

>BdACT* (Bradi3g22830; GenBank ON108680)

ATGTCTGTTTCCGTATCTAAATCTTCTCCAGTAGTAGTCAGACCAAGTTCAGAGCCTGCAACTTCTACTGCAGGT
AATTTGCTTAAGCTCAGCAGTTATGATAAGAACCATATGTGTATCCCTGTTACTATGTTCTTGGTCTTTGATAAC
CCAATTAATGAACCTGCTGAAACCATTAAATCTGCGTTGTCTCAAGCATTGGTACCATATTTACCATTTTCTGGG
AGACTTGCAGCTGAAAATAATGCAGTGCATATAACTTTCGGTGGAGATGACCAAGGGGTATCTTTTGTTGCAGCA
GCAGCATCATGTGGCCTCAAAGAGGTTGACTTATCAGATAGGTCCAGCCCCTTAACATCCACATTGTTAGATGAA

CTGGCAGTCTATTATCCAGCCGCCGCTTGTGGATTTGGTGATCCTCTCCTTCTTATGCAAGTTACAGAATTTACA
TGTGGCGGATTTGTAGTTGGTGTTACATGGAATCATGTAATAGCTGATGCTGCCGGAATGGGTCAATTTCTTAGG
GCTGTGGGAGAAAGAGCAAGGGGCTTGACTTTGGCTAGCTCAGTAGTACCCGTAAGGTGGGATGCTGCACTAAAG
GCAGCACCACCGGCAGCAGCAGGCTTTGTCGAATTGATGATGGGTCTAAAACCAATGGCTGATGTCGCTATTTTG
GATGTTACGGTTCCTTCTAGGTTAATTGGAAGTGTGAAAGATGCTTTTAGACGTCGTGGTAAATCCCCTTGTACA
ACTTTTGAAGCAGTGGCAGCAGTACTCTGGCGTTGTAGGACGAGGGCTATTCTTTCTGGTAGTTCATCCGACAGT
TCAGAGATCAATCCAGTAGTCGCATTATACTTTACAGTTAATGCAAGGAACTATGCAGGTGCTCGCGAAGGTTAT
TATGGAAATTGTGTTACAGGAAGATTTGTTGTTGCAACAGGTGGCACTGTCGCAAATGGGGATTTAGTTGATGTA
GTTGAAATGATTCGTCGAGCAAAAGAACAAATTCCTGAACAATTTAAAGAGGGTGCTGGTATAGGTAATGGTAAT
GATGGTGTCAGACAACAACAAGTGAACGTGGATGGACTTGTGGGATATAACTTATTCATTGTCACTTGTTGGAGA
AATCTAGGTGTGGATGAAGCAGACTTTGGTGGTGGCAGACCAGCTAGAGTTACATCTCATACAAGACAAGAAGTT
ACTGCACCTGCTTGTGTCGCTTGTCCACCTTTTCGCGGTAATGATGGAGCTAATGTACTCTCTGGTTGTGTTCGC
AAAGAACATGCTGATGCCTTCTTAGCTGAGCTCGCTCTTTCTAGTAGAACCCCGGCCCTTTTGGACTCATGA

SI Materials and Methods. Sampling and preparation of wheat tissues for RT-PCR analysis

Bread wheat cv. ‘Chinese Spring’ plants grown in hydroponic cultures were used for collection of coleoptile, root and root tip (5 mm terminal sections) tissues. Sterilized seeds were transferred to sterile polyacrylamide beads (Scotts) equilibrated with Hoagland’s medium #2. Tissues were harvested after 5 days of incubation under controlled conditions (16 h/ 8 h light/dark photoperiod, 23ºC). Tissues of stem, inflorescence and leaf infected with mildew (*Blumeria graminis f. sp. tritici* isolate FAL92315) were harvested from 9-week-old plants grown in a greenhouse. Leaf and wounded leaf tissues were harvested from 12-day-old plants grown in a controlled environment (16 h/ 8 h light/dark photoperiod, 18ºC during daytime, 13ºC at night). Wounded leaf tissue was collected 3 h after wounding with forceps. For root tips infected with *Gaeumannomyces graminis* (Take-all), tissues were collected from sterile plants germinated on a fungus-containing substrate. 5 mm terminal root sections were collected 6 d after sowing. For preparation of substrate, fungus was grown in 200 ml PD medium at 22ºC/130 rpm for 4 d, mycelium washed 5 times with Hoaglands medium #2 and mixed with 20 ml polyacrylate beads (prepared by addition of 5 gr of cross-linked polyacrylate (Miracle-Gro) to 250 ml of Hoagland’s medium #2, equilibration of hydrated beads with 3 x 250 ml of medium and subsequent autoclaving).

SI Materials and Methods. Quantitative real-time PCR (qRT-PCR) of wheat

RNA was extracted using TRI reagent (Sigma-Aldrich), according to manufacturer’s protocol. Following DNase treatment (RQ1, Promega), RNA was reverse-transcribed with M-MLV reverse-transcriptase (ThermoFisher Scientific) using a 1:1 mix of random hexamers and oligo(dT) primers. All oligonucleotides (SI appendix, Table S10) were designed using Primer3 software (1), with at least one homoeolog-specific oligo per each pair used. qRT-PCR was performed on a CFX96 Touch Real-Time PCR instrument (Bio-Rad) in the following conditions: initial step in the thermal cycler for 3 min at 95°C, followed by PCR amplification for 40 cycles of 10 s at 95°C and 30 s at 59°C, and finally dissociation analysis to confirm the specificity of PCR products with 0.5°C ramping from 55°C to 95°C. Each 10 µl reaction was comprised of 5 µl LightCycler 480 SYBR Green I Master mix (Roche Life Science), 2 µl cDNA template, 2 µl H_2O and 1 µl primer mix (0.5 µM each primer). Relative transcript levels were calculated according to the Pfaffl method (2), using the housekeeping gene β-tubulin (TUBB) as reference (3).

SI Materials and Methods. Quantitative real-time PCR (qRT-PCR) of *B. distachyon*

B. distachyon accession Bd3-1 seeds were soaked, peeled, and placed between three filter paper layers soaked in 5 ml water. The seeds were stratified for 5 days at 5°C in the dark and one day at 22°C (16h/8h - light/dark photoperiod) in a controlled environment growth cabinet. For Fusarium root rot (FRR) material, ten stratified seeds were placed on 9 cm^2 filter square paper on 50 ml 0.8% water agar. All plates were placed in a plant propagator with water-soaked paper towels, angled 20° from the upright position, and stored for 3 days at 22°C (16 h/ 8 h - light/dark photoperiod, variable humidity). *Fusarium graminearum* isolate PH1 was maintained on potato dextrose agar (PDA) at 22°C 16 h/ 8 h - light/dark photoperiod in a controlled environment growth cabinet. One 9 cm diameter Petri-dish of seven-day old *F. graminearum* mycelia was blended to a slurry with 1 ml water and applied to three points on each root (root tip, mid root, and near seed) using a 10 ml syringe. The inoculum slurry was removed once infection was visible at 1 dpi and the roots were rinsed with sterile distilled water. Immediately after and then every two days, ten roots per plate (one biological replicate pool) were cut and flash frozen in liquid nitrogen. For Fusarium Head Blight (FHB) material, seeds were sown in 50% peat/sand and 50% John Innes mix 2 (two seeds per 8 cm^2 pot). Plants were then maintained for six weeks at 22°C (20 h/ 4 h - light/dark photoperiod, 70% humidity) in controlled environment growth cabinet until mid-anthesis. Before the dark period, pots and matting was watered until run-off, spikes were inoculated with 1 x 10^6 spores/cm^2 amended with 0.05% Tween-20, and all plants were enclosed in clear plastic bags to maintain high humidity for three days. Immediately after and then every two days, three spikes from different plants were pooled and flash frozen in liquid nitrogen. For conidial suspension inoculum, Mung Bean (MB) broth (4) with a 1 cm^2 *F. graminearum* PDA mycelial plug was incubated at 23-25°C, 200 rpm for seven days. The inoculum was filtered with cheesecloth and quantified using a haemocytometer.

RNA from FHB, FRR, and control samples was extracted using a QIAGEN RNAeasy plant mini kit as per standard protocol. RNA was then immediately cleaned using Turbo DNA-free kits (Invitrogen) as per standard protocol except for two rounds of Turbo DNAse treatment. Subsequently cDNA was prepared using SuperScript III Reverse Transcriptase (Thermo Fisher Scientific), as per standard protocol. All oligonucleotides (SI appendix, Table S10) were designed using Primer3 software (1). Reverse transcriptase qPCR was performed in a Framestar-480/384 well plate containing 5 µl of 2x SYBR Green JumpStart Taq ReadyMix (Sigma-Aldrich), 2 µl cDNA, 0.6 µl of 10 µM per primer, and 1.8 µl water per well. The thermocycling protocol 300 s 95°C, 45x(94°C 10 s, 58°C 10 s, 72°C 10 s, 75°C 2 s (single acquisition)), followed by dissociation analysis by ramping from 65°C to 97°C, was performed on a Roche LightCycler LC480. Cq values and primer efficiency were quantified using the LinRegPCR tool (5). Relative quantification was calculated according to the Pfaffl method (2), using the housekeeping gene GAPDH as reference.

SI Materials and Methods. Generation of DNA constructs

For cloning of *Brachypodium distachyon* and wheat genes, RNA was extracted from mature plant leaves of *B. distachyon* (accession Bd21) or leaves from 10-day-old *Triticum aestivum* plants (Chinese Spring), infected with powdery mildew (*Blumeria graminis f. sp. tritici*), using RNeasy plant mini kit (Qiagen). RNA was treated with RQ1 DNAse (Promega) and cDNA libraries prepared with Superscript IV or Superscript III reverse transcriptase kits (Thermo Fisher Scientific), using oligo(dT) primers, according to manufacturer's protocols. TaOMT3, TaOMT6, TaOMT8, TaCYP71C164_5D, TaCYP71F53_5D, BdOSC2, CYP51H13P/H13_5A/14/H15/H16/H35/H37 were amplified from cDNA using Phusion DNA polymerase (Thermo Fisher Scientific) or Q5 DNA polymerase (New England Biolabs). TaOSC_5D, TaHSD, AsOSC1, AsCYP51H73 were synthesized by General Biosystems, Durham, NC, USA. BdACT, BdMeTr, TaCHS1, chi-1D, TaCPS-D2, TaKSL-D1 and *Taxus canadensis* GGPPS were synthesized by Twist Bioscience, San Francisco, CA, USA. TaCPS-D2, TaKSL-D1 and TcGGPPS lack signal sequences, to allow for cytosolic localization in *N. benthamiama* expression (6). *A. tauschii* IAS coding sequence was derived from TaOSC_5D sequence with site directed mutagenesis (7) to obtain a single mutation (I581S). Synthesized and cDNA-amplified genes from triterpene and diterpene BGCs were cloned into a pCAMBIA-based (8) plant expression vector with Goldenbraid cloning (9), using BsaI and BsmbI (New England Biolabs) and T4 DNA ligase enzymes (New England Biolabs). Gene expression in final vectors is driven by *Solanum lycopersicum* ubiquitin 10 promoter and terminator (10). Synthesized and cDNA-amplified genes from flavonoid cluster were cloned into a pDONR207 Gateway entry vector and subcloned into a pEAQ-HT-DEST1 plasmid (11) using BP and LR clonase enzyme mixes (Thermo Fisher Scientific), respectively.

SI Materials and Methods. GC-MS metabolite extraction and analyses

Metabolite extraction from agroinfiltrated *N. benthamiana* leaves for GC-MS analyses

Diterpenes: for analysis of TaCPS-D2 and TaKSL-D1 transient expression, 5 mg of *N. benthamiana* leaf samples were extracted in 850 µl ethyl acetate for 1 h in room temperature, with agitation. Following removal of plant tissue by centrifugation, 750 µl from each extract was evaporated and reconstituted in 75 µl ethyl acetate. Triterpenes: for analysis of wheat BGC 3(5D) genes expression, 5 mg samples were extracted in 500 µl ethyl acetate with 5 µg/ml 5α-cholestan-3β-ol. For analysis of *B. distachyon* brachynacin cluster genes expression, 5 mg samples were extracted in 500 µl methanol with 5 µg/ml 5α-cholestan-3β-ol. For analysis of oat and *A. tauschii* OSC and CYP51 genes, 5 mg samples were extracted in 500 µl ethyl acetate. For analysis of combined expression of wheat BGC 3(5D) genes and BdACT, 5 mg samples were extracted in 300 µl ethyl acetate. For analysis of combined expression of *B. distachyon* brachynacin cluster genes and TaHSD, 5 mg samples were extracted in 500 µl ethyl acetate with 5 µg/ml 5α-cholestan-3β-ol. All triterpene extractions from *N. benthamiana* leaves were done in room temperature for 1 hour, with agitation. For all triterpene extractions, following the removal of plant tissue by centrifugation, 200 µl were evaporated and reconstituted in 70 µl TMS+pyridine (Sigma-Aldrich). Samples were derivatized for 0.5 h in 70ºC.

Metabolite extraction from MeJA-treated wheat and *B. distachyon* leaves for GC-MS analyses

MeJa-treated wheat leaf sections were freeze-dried and ground. 25 mg from each sample were extracted in 800 µl ethyl acetate containing 5 µg/ml 5α-cholestan-3β-ol, with agitation for 2 h in 40ºC. Following removal of tissue by centrifugation and filtration with 0.22 µl filter mini columns (Norgen), 700 µl from each extract was evaporated and reconstituted in 70 µl TMS with pyridine (Sigma-Aldrich). Samples were derivatized for 0.5 h in 70ºC. MeJa-treated *B. distachyon* leaf sections were freeze-dried and ground. 25 mg from each ground sample were extracted in 1100 µl methanol containing 2.5 µg/ml 5α-cholestan-3β-ol, with agitation for 2 h in 40ºC. Following removal of tissue by centrifugation and filtration, 800 µl from each extract was evaporated and reconstituted in 70 µl TMS with pyridine (Sigma-Aldrich). Samples were derivatized for 0.5 h in 70ºC.

GC-MS analysis of diterpenes and triterpenes from *N. benthamiana* and grasses leaf extracts

GC-MS analysis was performed using an Agilent 7890B instrument with a Zebron ZB5-HT Inferno column (Phenomenex). For triterpenes analysis, a previously described method (12) was used: injections were performed in pulsed splitless mode (30 psi pulse pressure). Inlet temperature was set to 250°C. GC oven temperature was initially held at 170°C for 2 mins, subsequently ramped to 300°C at 20°C/min and held at 300°C for an additional 11.5 min (20 min total run time). The GC oven was coupled to an Agilent 5977B MS detector set to scan mode, from 60 to 800 mass units (solvent delay 8 min). For semi-quantification of brachynacin in *B. distachyon* leaves, Selected Ion Monitoring (SIM) mode was used, for detection of brachynacin (m/z 170.1, 340.2, 387.3, 400.3, 445.4, 475.4, 500.4 ions were monitored) and internal standard 5α-cholestan-3β-ol (m/z 215.1, 355.4, 445.5, 460.5 ions were monitored), with 100 ms dwell time for each ion. Diterpenes analysis was based on a previously described method (6): injections were performed in splitless mode. Inlet temperature was set to 280°C. GC oven temperature was initially held at 130°C for 2 mins, subsequently ramped up to 250°C at 8°C/min, followed by ramping up to 310°C at 10°C/min and held at 310°C for an additional 5 min (28 min total run time). The MS detector was set to scan mode, from 50 to 550 mass units (solvent delay 4 min).

SI Materials and Methods. LC-MS analyses

Metabolite extraction from agroinfiltrated *N. benthamiana* leaves for LC-MS analyses

For LC-MS analysis of recombinantly expressed wheat BGC 3(5D) genes (including combined expression with BdACT), 25 mg of each sample were extracted in 2 ml methanol in room temperature for 1 h, with agitation. Following removal of plant tissue by centrifugation, extracts were partitioned twice with 2 ml hexane and filtered with 0.22 µl filter mini columns (Norgen). Extracts were evaporated and resuspended in 100 µl methanol. For analysis of recombinantly expressed *B. distachyon* brachynacin cluster genes (including combined expression with TaHSD), 10 mg of each sample were extracted in 400 µl 80% methanol in room temperature for 1 h, with agitation. Following removal of plant tissue by centrifugation, extracts were partitioned with 500 µl hexane and filtered. Extracts were evaporated and resuspended in 100 µl 80% methanol. For analysis of wheat flavonoid cluster genes,

250 mg freeze-dried and ground samples were extracted with 4 mL methanol at room temperature for 1 h. Extracts were fully evaporated, resuspended in 200 µL methanol, and filtrated through a mini column (pore size 0.22 µm, Geneflow). Filtered samples were transferred to glass autosampler vials and 20 µL of each sample was analyzed by UHPLC-CAD-PDA-MS.

LC-MS analyses of triterpenes from *N. benthamiana* leaf extracts

Leaf extracts were analyzed by reverse phase HPLC on a Shimadzu LCMS-2020 single quadrupole mass spectrometer coupled with a Dionex Corona Veo RS charged aerosol detector (Thermo Scientific), using a Kinetex 2.6 µm XB-C18 100 Å, 50 x 2.1 mm LC Column (Phenomenex). MS data was collected using combined electrospray ionization (ESI) and atmospheric pressure chemical ionization (APCI) in positive mode. 10 µl samples were injected using 12 min, 14.5 min or 30 min mobile phase gradient methods using solvent A- water with 0.1% formic acid and solvent B- methanol with 0.1% formic acid, as follows. 12 min method: 50% B hold from 0 to 0.75 min, 50% to 90% B from 0.75 to 8 min, 90% B hold from 8 to 10 min, 90% to 50% B from 10 to 10.5 min, 50% B hold from 10.5 to 12 min. Flow rate, 0.4 ml/min. MS scan, m/z 250 – 1900. Column oven temperature, 40ºC. 14.5 min method: 70% to 95% B from 0 to 10 min, 95% B hold from 10 to 11 min, 95% to 70% B from 11 to 11.1 min, 70% B hold from 11.1 to 14.5 min. Flow rate, 0.5 ml/min. MS scan, m/z 200 – 1200. Column oven temperature, 30ºC. 30 min method: 15% B hold from 0 to 0.15 min, 15% to 60% B from 0.15 to 26 min, 60% to 100% B from 26 to 26.5 min, 100% B hold from 26.5 to 28.5 min, 100% to 15% B from 28.5 to 29 min, 15% B hold from 29 to 30 min. Flow rate, 0.3 ml/min. MS scan, m/z 100 – 1500. Column oven temperature, 30ºC.

LC-MS analyses of flavonoids from *N. benthamiana* leaf extracts

Leaf extracts were analyzed by reverse phase HPLC on a Shimadzu LCMS-2020 single quadrupole mass spectrometer coupled with a Dionex Corona Veo RS charged aerosol detector (Thermo Scientific) and a SPD-M20A HPLC Photodiode Array Detector (PDA; Shimadzu), using a Kinetex 2.6 µm XB-C18 100 Å, 50 x 2.1 mm LC Column (Phenomenex), kept at 30°C. Water containing 0.1% formic acid (FA) and acetonitrile containing 0.1% formic acid (FA) were used as mobile phases A and B, respectively, with a flow rate of 0.2 mL/min. A gradient elution program was applied as follows: 0-1.5 min linearly increased from 0% to 10% B, 1.5-26 min linearly increased from 10% to 60% B, 26-26.5 min linearly increased from 60% to 80% B, 26.5-28.5 min linearly increased from 80% to 100% B, 28.5-29 min linearly decreased from 100% to 10% B hold on for another 1 min for re-equilibration, giving a total run time 30 min. MS detection was performed in both positive and negative ESI range of *m/z* 50–1500 with the following settings: desolvation temperature was 250°C; drying gas flow, 15 L/min; detector voltage was 1.25 kV; and nebulizing gas flow, 1.5 L/min. PDA chromatograms were recorded in a 200–600 nm range using a deuterium (D2) and tungsten (W) light source.

High-resolution mass spectrometry analysis of the metabolites was carried out on a Q Exactive instrument (Thermo Scientific). Chromatography was performed using a Kinetex 2.6 µm XB-C18 100 Å, 50 mm x 2.1 mm (Phenomenex) column kept at 30°C. Water containing 0.1% formic acid (FA) and acetonitrile containing 0.1% formic acid (FA) were used as mobile phases A and B, respectively with a flow rate of 0.4 mL/min. A gradient elution program was applied as follows: 0-0.75 min linearly

increased from 0% to 10% B, 0.75-13 min linearly increased from 10% to 60% B, 13-13.25 min linearly increased from 60% to 80% B, 13.25-14.25 min linearly increased from 80% to 100% B, 14.25-14.5 min linearly decreased from 100% to 10% B hold on for another 2.5 min for re-equilibration, giving a total run time 17 min. MS detection was performed in both positive and negative ESI range of 100–1500 *m/z*. The analysis of the 3D field of the Photodiode-Array Detection (PDA) was recorded in a 200–600 nm range using a vanquish detector (Thermo Scientific).

SI Materials and Methods. Purification of compounds from large-scale *N. benthamiana* agroinfiltration

Purification of 19-hydroxy-isoarborinol

160 6-weeks old *N. benthamiana* plants were manually infiltrated with *Agrobacterium tumefaciens* strains containing pEAQ-HT-DEST1 vectors (11) expressing TaIAS and TaIAH. For infiltration, agrobacteria strains were mixed 1:1 in MMA buffer (10 mM $MgCl_2$, 10 mM MES/KOH pH5.6, 150 μM acetosyringone) to a final concentration of $O.D._{600}$ 0.2 per each strain. Infiltrated leaves were harvested 6 days post infiltration and lyophilized. 111 gr of powdered leaf material was extracted twice with 15 ml/gr ethanol, using a Buchi Speed extractor E916 (100ºC, 100 bar). The extract was saponified by addition of 35 ml water and 15 gr KOH per 150 ml of extract, and incubation for 2 h at 65ºC. Following addition of 50 ml water per 150 ml of extract, the saponification reaction was extracted three times with 100 ml hexane. Solvent was removed by rotary evaporation and residual was applied to a flash chromatography column (35mm diameter) filled with 100 ml of silica (LC60A35-70 µm). 19-hydroxy-isoarborinol was eluted from the column with 1:1 hexane:ethyl acetate. Fractions containing the product were pooled and evaporated. Decolorization with addition of a minimal amount of activated charcoal was followed by recrystallization in an ethanol-H_2O mix, to give a final yield of 20 mg purified compound.

Purification of ellarinacin

100 *N. benthamiana* plants were infiltrated by vacuum (13) with *A. tumefaciens* GV3101 strains containing vectors for expression of TaIAS, TaIAH, TaHID, TaHIO and oat tHMGR. For infiltration, agrobacteria strains were mixed in MMA buffer to a final concentration of $O.D._{600}$ 0.2 per each strain. Infiltrated leaves were harvested 8 days post infiltration and lyophilized. 129 gr of powdered leaf material was extracted with ethyl acetate using a Buchi Speed extractor E916, and chlorophylls were removed from the extracts by addition of ion-exchange resin, following the protocol detailed in[2]. Three successive rounds of fractionation were performed on an Isolera Prime flash chromatography system (Biotage), with hexane-ethyl acetate gradients specified in Table S11, to yield 10.3 mg of purified compound.

Purification of brachynacin

100 *N. benthamiana* plants were infiltrated by vacuum with *A. tumefaciens* GV3101 strains containing vectors for expression of BdIAS, BdIAH, BdHIH, BdTIH, BdACT and oat tHMGR. For infiltration, agrobacteria strains were mixed in MMA buffer to a final concentration of $O.D._{600}$ 0.15 per each strain.

Infiltrated leaves were harvested 9 days post infiltration and lyophilized. 69 gr of powdered leaf material was extracted with methanol using a Buchi Speed extractor E916, following the protocol detailed in[2]. Two successive rounds of fractionation were performed on an Isolera Prime flash chromatography system (Biotage), with hexane-ethyl acetate gradients specified in Table S11. Product-containing fractions from flash chromatography were pooled and evaporated, and resulting product was dissolved in 1 ml 95% MeOH for semi-preparative HPLC purification on an Agilent 1290 Infinity (II) system. A Luna 5 µm C18(2) 100 Å, 250 x 10 mm LC column (Phenomenex) was used, with column oven temperature set at 25ºC. A mobile phase gradient was run at 3.5 ml/min with buffers A- 0.1% formic acid in H_2O, and B- 0.1% formic acid in acetonitrile, as follows: 20-60% B from 0-25 min, 60-100% B from 25 to 25.5 min, 100% B from 25.5 to 28 min, 100-20% B from 28 to 28.5 min, 20% B from 28.5 to 30 min. 0.5 ml of sample was injected in five 100 µl injections. Eluent was monitored by an Evaporative Light Scattering Detector (ELSD). Collected fractions containing brachynacin product were pooled and evaporated, to yield <2 mg of purified compound.

SI Materials and Methods. Computational analyses

Regulatory network analysis

Target gene-transcription factor interactions and GO term enrichment tables were extracted from a GENIE3-generated wheat regulatory network (14), available at https://doi.org/10.5447/ipk/2018/7. Network visualization was done with Cytoscape v3.8 (15). Genbank accessions for benzoxazinoid pathway genes were retrieved from (16) and matched with IWGSC gene IDs by BlastN on EnsemblPlants (http://plants.ensembl.org). WGCNA and GENIE3-generated regulatory network (14) were generated using IWGSC RefSeq v1.0 gene models. Other analyses described in this manuscript are based on RefSeq v1.1 gene models.

Co-expression analysis

Co-expression within each cluster was assessed by calculation of the Pearson correlation coefficient (r-val) between the expression of a representative scaffold-forming gene from each cluster (*i.e.*, TPS in clusters 1(2A), 1(2D) and 2(2B), OSC in clusters 3(5A) and 3(5D), and chalcone synthase in cluster 4(5D)), and other genes in the cluster, within an RNA-seq dataset including 68 experiments from the wheat-expression.com website (14, 17).

Pairwise alignment with orthologous clusters in wheat ancestral species

Peptide sequences were extracted from EnsemblPlants (http://plants.ensembl.org): *Aegilops tauschii* (Aet_v4.0) (18), *Triticum turgidum subsp. dicoccoides* (WEWSeq_v1.0) (19), *Triticum aestivum* (IWGSC) (20). Gene models were manually corrected to obtain full coding sequences, and putative protein sequences were aligned using LALIGN (http://www.ebi.ac.uk).

Microsynteny analyses

To perform microsynteny analysis and generate figures, a python implementation of MCScan (21), https://github.com/tanghaibao/jcvi/wiki/MCscan-(Python-version), was used. FASTA and GFF3 files were retrieved from EnsemblPlants (http://plants.ensembl.org) for chromosomes 5A, 5B and 5D of *Triticum aestivum* (IWGSC), 5A and 5D of *Triticum turgidum subsp. diccocoides* (WEWSeq_v.1.0) and 5D of *Aegilops tauschii* (Aet_v4.0). MCScan ortholog finding and synteny assignment was run with a c-score of 0.99 and a single iteration. For wheat-rice analysis, wheat Triticum_aestivum_4.0 (22) and rice IRGSP-1.0 (23) assemblies were used.

Genomic positioning of wheat BGC homologs in other grasses

Protein sequences of all co-expressed genes from wheat BGCs 1(2D), 2(2B), 3(5D) and 4(5D) were used as BlastP queries against the following genome assemblies: *Zea mays* B73 RefGen_v4 (24), *Hordeum vulgare* cv. Morex r1 (25), *Brachypodium distachyon* Bd21 v3.1 (26), *Oryza sativa ssp. japonica* cv. Nipponbare v7.0 (27) and *Avena strigosa* S75 v2.0 (28). BlastP searches in all assemblies except *Avena strigosa* were performed in Phytozome13 (https://phytozome-next.jgi.doe.gov/) (29), using default parameters. Genomic locations of top BlastP hits in each species were visualized using Circos software v0.69-9 (30).

SI Materials and Methods. Plant treatment with elicitors and pathogens

Inoculation of detached wheat leaves with powdery mildew

For gene expression profiling by qRT-PCR, detached leaves from 10-day-old Chinese Spring wheat plants, grown in a growth cabinet (18˚C, 16 h day-length under fluorescent lights supplemented with near-UV lights and 12˚C for 8 h in the dark), were inoculated with *Blumeria graminis f. sp. tritici* (isolate FAL92315, maintained on the susceptible wheat cv. Cerco), or with *Blumeria graminis f. sp. hordei* (CH4.8 isolate, maintained on the susceptible barley cv. Golden Promise). Non-inoculated detached leaves kept in same conditions were used as controls. Leaf segments of ~4 cm length were placed in boxes containing water with 0.5% agar and 100 mg L^{-1} benzimidazole, and were inoculated by blowing fresh spores into settling towers placed over the plant material, according to the method of (31). Following inoculation, plant material was kept in growth cabinet at constant temperature of 15ºC and 16 h day-length, and samples collected 12 h and 24 h post-inoculation.

Treatment of detached wheat leaves with elicitors

2-3 cm leaf sections were cut from 1st leaf of 10-day old Chinese Spring seedlings grown in soil. Leaf sections were kept in H_2O in a Petri dish for 24 hours in a 22ºC lighted growth cabinet (16 h/ 8 h light/dark photoperiod), then transferred to Parafilm-sealed Petri dishes containing different solutions and kept in one cabinet under the same conditions as pre-treatment: 150 µM methyl jasmonate (Sigma-Aldrich), 500 µM salicylic acid, pH 6.0 (Sigma-Aldrich), 0.5 mg/ml chitin oligosaccharides (NaCoSy, YSK, Japan) or H_2O. All solutions also contained 0.02% Tween-20. Samples for qRT-PCR analysis

were collected after 2 h or 12 h. Four biological replicates of MeJa-treated leaves were collected after three days of treatment for GC-MS analyses.

Treatment of *B. distachyon* with methyl jasmonate

Sections were cut from aerial parts of *B. distachyon* Bd21 plants grown in soil for 2.5 weeks. Samples were kept in H_2O in a Petri dish for 24 h in a 22ºC lighted growth cabinet (16 h photoperiod), then transferred to Parafilm-sealed Petri dishes containing 150 μM methyl jasmonate and 0.02% Tween-20, or 0.02% Tween-20 in H_2O, and kept in one cabinet under the same conditions as pre-treatment. Four biological replicates of samples were collected for GC-MS analysis after three days.

SI Figures

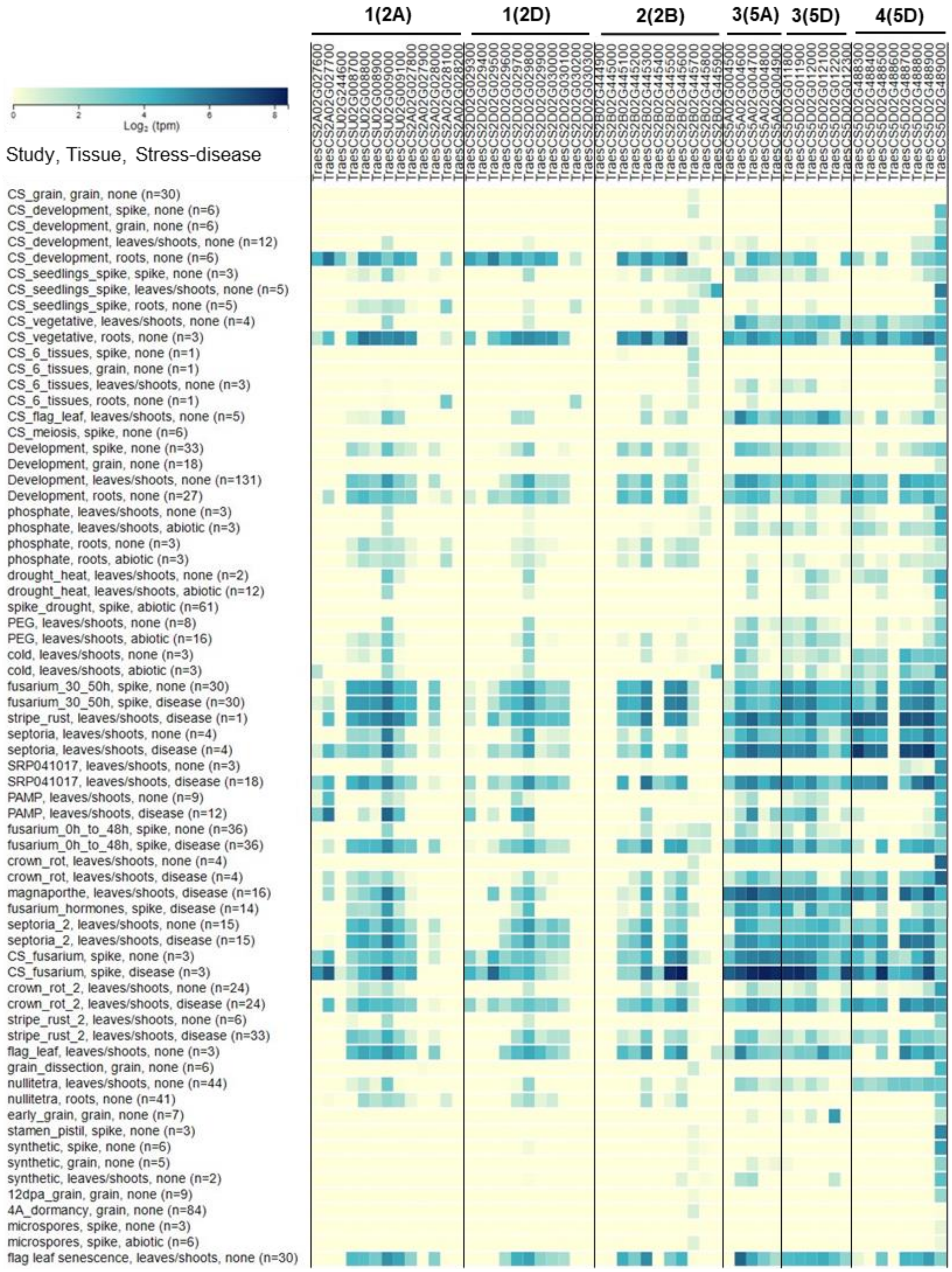

Fig. S1. Gene expression data of six wheat biosynthetic gene clusters. Log2 of normalized values (transcripts per million) are shown, derived from RNA-seq data at http://www.wheat-expression.com (14, 17).

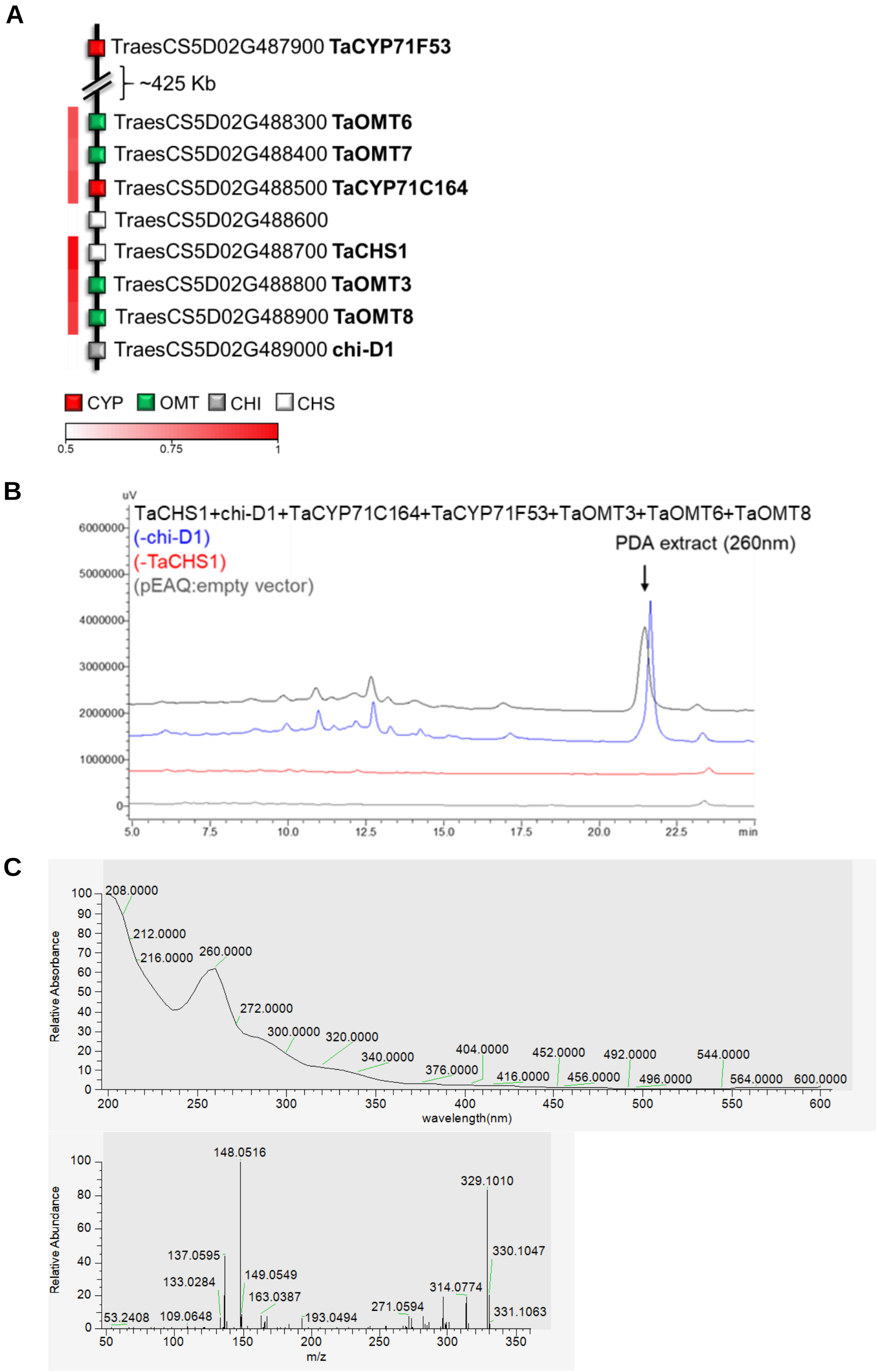

Fig. S2. Wheat cluster 4(5D). (A) Names and IWGSC IDs of wheat cluster 4(5D) genes. White to red color-coding denotes Pearson correlation (r) values for expression of each gene with TaCHS1. (B) LC-PDA analysis of cluster 4(5D) genes expressed in *N. benthamiana*. The putative end-product of the pathway, marked with an arrow, is formed by expression of the complete cluster or with the absence of chi-D1, but not formed when TaCHS1 is not expressed. (C) UV-VIS and ms^2 spectra of the [M+H=329.1] ion produced by cluster 4(5D).

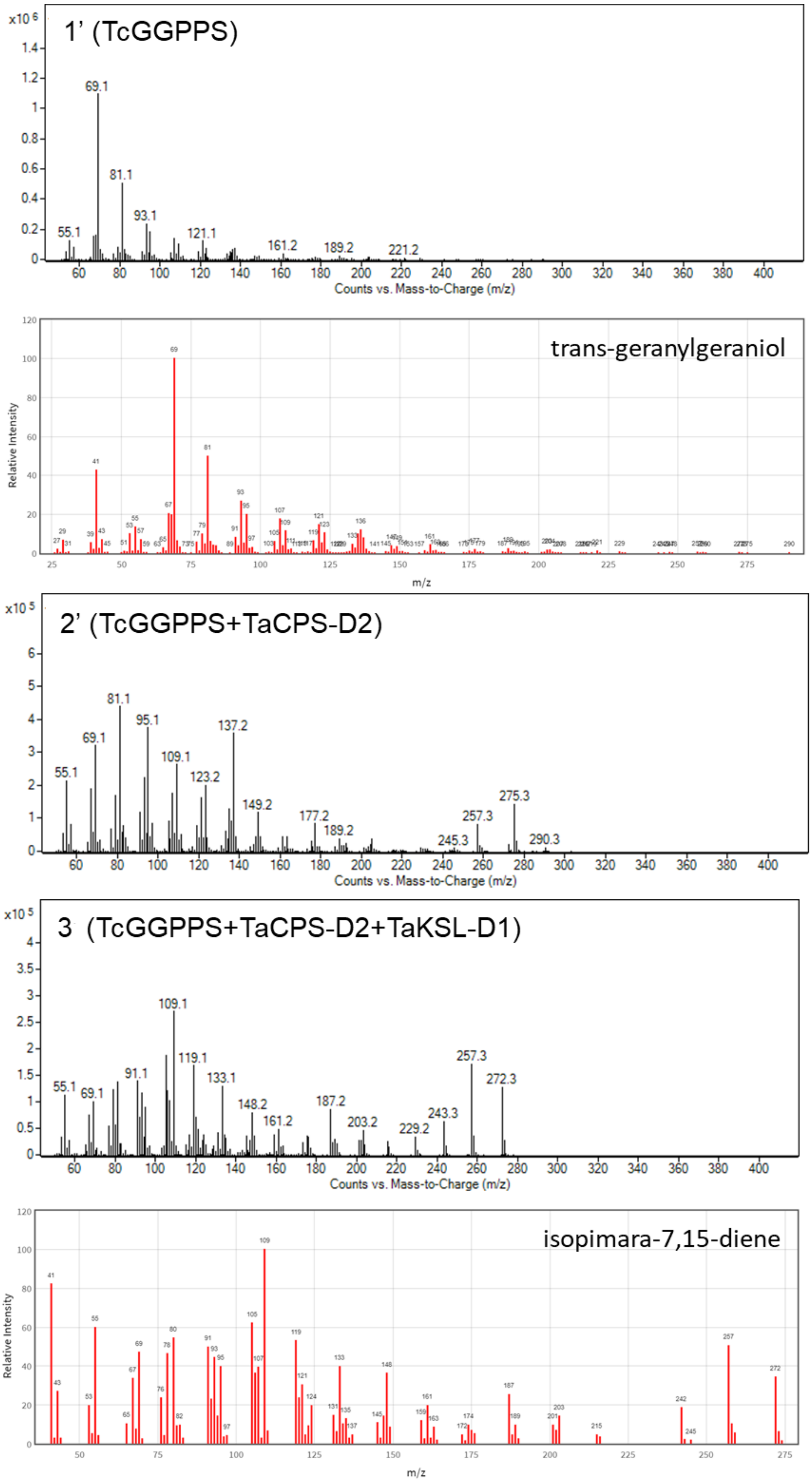

Fig. S3. Mass spectra of wheat TaKSL-D1 and TaCPS-D2 products in *N. benthamiana* expression. TaKSL-D1 and TaCPS-D2 were transiently expressed together with *Taxus canadensis* GGPP synthase and oat tHMGR. Mass spectra are shown at retention times of peaks putatively identified as geranylgeraniol (1'), copalol (2') and isopimara-7,15-diene (3), based on comparison to NIST Chemistry WebBook (1', 3) (https://webbook.nist.gov/chemistry) and literature (2') (32). tHMGR was included in all combinations of genes.

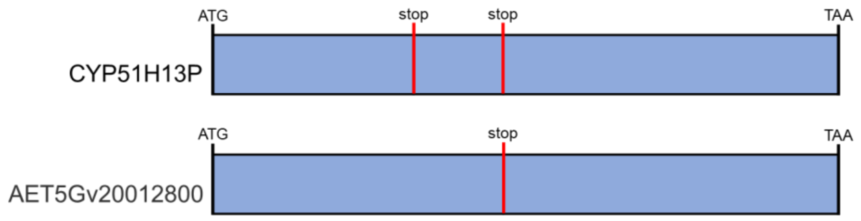

T. aestivum CYP51H13P coding sequence (manually corrected)

ATGGACTTGACAAGTCTCACTGCCATGTGGTGGGCCATAGCTCTTCTTTTCATTACTGTATTGGCCACCAAGATCACAAGAGCAAGAATCACCAACATTGATCCACAGCG
TACAACAGGCCAACTTCCACCCATGGTGAATGGACTTGCTCTCCTAGGATTATTACCTACCCTTCTGAAGAAGGGTCTACCACCTATGGTCAATTATCTATATGTTAACTAT
GGCAGTGTCTTCACTGTAAGTTGCTTTGGAGTGATCAAGGTAACACTCTTGATCGGGCCAGAGGCGACGACTCATTTCTTCCAAGGTTTGGAGTCAGAGATTAGCCAT
GGTAATCTGCTTGAGTTCACAGTGCCCATGTTTGGCAAGGCAGTTGGTTATGGTAGAGATACCGCCACCCGAATGGAACAAATGCGCTTCCACAGTGAAGCACTGAGG
GCATCCAGGTTAAGGAGCCATGTTTCTCCAATGCTTCAAGAAGTGGAGGATTTCTTTGCAAAGTGGGGAGAGGAAGGCGTAGTTGATCTAAAGCTTGAGTTCTAGCAG
CTACTTATGTTGATATCAGGCTGATGTTTGCTTGGAAAAGAGGTCCGGGAGAATATGTTTGATGAAGTCTACACACTTTTTCGCGAGATCGAAAAGGGTGTGACCTTGAT
CAGCTTCTTGTTCCCATATCTCCCAACTCCAGCAAACTGGCAGCGTGATAGGGCGCGTATAAGGCTAACAGAGATCCTCTCTAATGTTGTCGAATCCCGTAAGAGCTCC
GGGCGAGTGGAGGAGGACACGCTGCAGAAACTGATTGACTCCAAGTACAAAGATGGTCGCCCTACAACAGTAGAAGAGGTAGTCGGGTTGATCATTGGCTTGCTGTT
TGCTGGAAAACACACCAGCTCTCATACTAGTACTTGGACTGGAGCTTGTCTTCTCAGCCATCCAGCCTTCTTAAGAGCTGCCATTGAGGAGCAACATCAAGTCGCCAAG
AAATACAAGGACGGACTAGAATACAATGCCTTCTTAGAGATGGATACGCTGCATAATTGCATCAAGGAGGCGCTGCGGAAGAACCCTGCGACACCACTGCTGGTTCGC
AGGGTCCATAAGCGCTTCACGGTGCAGACGAAAGAAGGCCAACAATATGACATCCAGCAGGACTACATCGTAGCAACTCCTACTATAGTAAACAACAATATCCCTTACAT
TTATACGGACCCTCAGGTGTATGATCCAAATCGGTTTGGCCCCGAAAGAAGAGAGGACAAAGTGGGTGGCAAGTTCTCTTACACATCATTTGGTGGTGGAAGGCACGT
TTGCACCGGAGAGGCTTATGCTTACATGCAACTCAAAGTGATATGGAGCCATTTGCTGAGGAACTTTGATCTCGAATTGGTCTCTCCCTTCCCTAACACAGACTGGAGC
AAGTTCTTGCCAGATCCACAGGGAAAATTACTCGTGAGATATACGAGAAATGGAATTTAA

A. tauschii AET5Gv20012800 coding sequence (manually corrected)

ATGGACTTGACAAGTCTCACTGCCATGTGGTGGGCCATAGCTCTTCTTTTCATTACTGTATTGGCCACCAAGATCACAAGAGCAAGAATCACCAACATTGATCCACAGCG
TACAACAGGCCAACTTCCACCCATGGTGAATGGACTTGCTCTCCTAGGATTATTACCTACCCTTCTGAAGAAGGGTCTACCACCTATGGTCAATTATCTATATGTTAACTAT
GGCAGTGTCTTCACTGTAAGTTGCTTTGGAGTGATCAAGGTAACACTCTTGATCGGGCCAGAGGCGACGACTCATTTCTTCCAAGGTTTGGAGTCAGAGATTAGCCAT
GGTAATCTGCTTGAGTTCACAGTGCCCATGTTTGGCAAGGCAGTTGGTTATGGTAGAGATACCGCCACCCGAATGGAACAAATGCGCTTCCACAGTGAAGCACTGAGG
GCATCCAGGTTAAGGAGCCATGTTTCTCCAATGCTTCAAGAAGTGGAGGATTTCTTTGCAAAGTGGGGAGAGAAAGGCGTAGTTGATCTAAAGCTTGAGTTCGAGCAG
CTACTTATGTTGATATCAGGCTGATGTTTGCTTGGAAAAGAGGTCCGGGAGAATATGTTTGATGAAGTCTACACACTTTTTCGCGAGATCGAAAAGGGTGTGACCTTGAT
CAGCTTCTTGTTCCCATATCTCCCAACTCCAGCAAACTGGCAGCGTGATAGGGCGCGTATAAGGCTAACAGAGATCCTCTCTAATGTTGTCGAGTCCCGTAAGAGCTCC
GGGCGAGTGGAGGAGGACACGCTGCAGAAACTGATTGACTCCAAGTACAAAGATGGTCGCCCTACAACAGTAGAAGAGGTAGTCGGGTTGATCATTGGCTTGCTGTT
TGCTGGAAAACACACCAGCTCTCATACTAGTACTTGGACTGGAGCTCGTCTTCTCAGCCATCCAGCCTTCTTAAGAGCTGCCATTGAGGAGCAACATCAAGTCGCCAA
GAAATACAAGGACGGACTAGAATACAATGCCTTCTTAGAGATGGATACGCTGCATAATTGCATCAAGGAGGCGCTGCGGAAGAACCCTGCGACACCACTGCTGGTTCG
CAGGGTCCATAAGCGCTTCACGGTGCAGACGAAAGAAGGCCAACAATATGACATCCCGCAGGACTACATCGTAGCAACTCCTACTATAGTAAACAACAATATCCCTTACA
TTTATACGGACCCTCAGGTGTATGATCCAAATCGGTTTGGCCCCGAAAGAAGAGAGGACAAAGTGGGTGGCAAGTTCTCTTACACATCATTTGGTGGTGGAAGGCACG
TTTGCACCGGAGAGGCTTATGCTTACATGCAACTCAAAGTGATATGGAGCCATTTGCTGAGGAACTTTGATCTCGAATTGGTCTCTCCCTTCCCTAACACAGACTGGAG
CAAGTTCTTGCCAGATCCACAGGGAAAATTACTCGTGAGATATACGAGAAATGGAATTTAA

Fig. S4. Coding sequences of *T. aestivum TaCYP51H13P* and its *A. tauschii* ortholog, *AET5Gv20012800*. Early stop codons are marked in red. TaCYP51H13P is the product of two IWGSC annotated genes, *TraesCS5D01G012100* and *TraesCS5D01G012200*. Manual annotation of these genes showed that they represent a single transcript with two premature stop codons and were therefore designated as a single pseudogene. The manual annotation was verified by cloning and sequencing of the full transcript from a cDNA library.

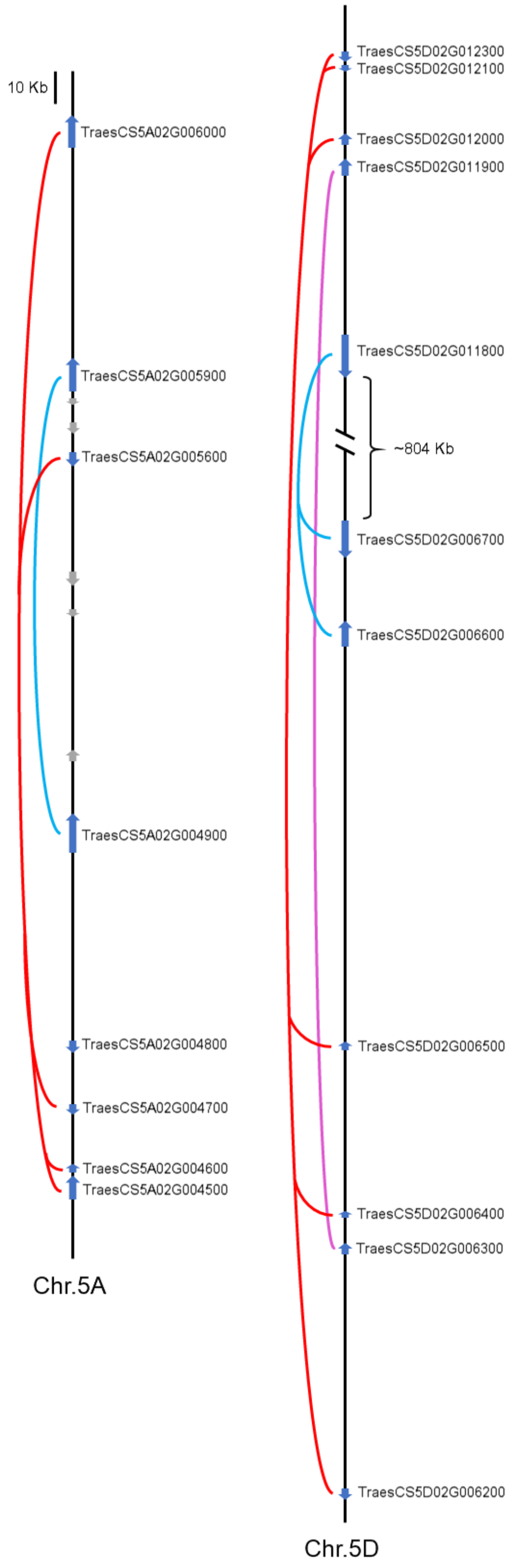

Fig. S5. Paralogs of the 3-5A and 3-5D cluster genes on wheat chromosomes 5A and 5D. Blue lines connect paralog OSC genes, red lines CYP51 genes, and pink line HSD genes.

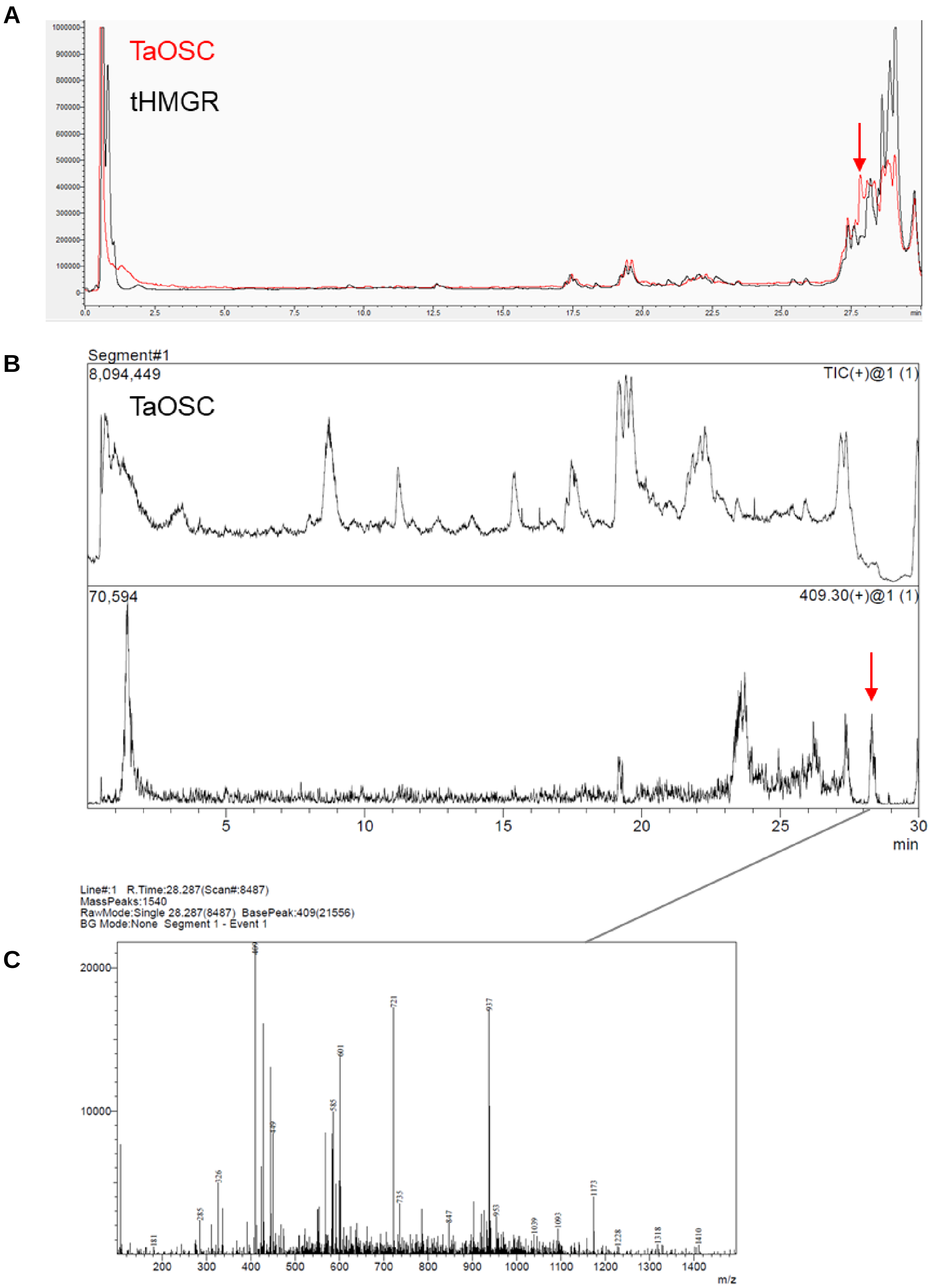

Fig. S6. LC-MS detection of isoarborinol molecular ion. (A) CAD chromatogram. (B) MS chromatogram- TIC and EIC of 409.3. (C) Mass spectra at peak retention time.

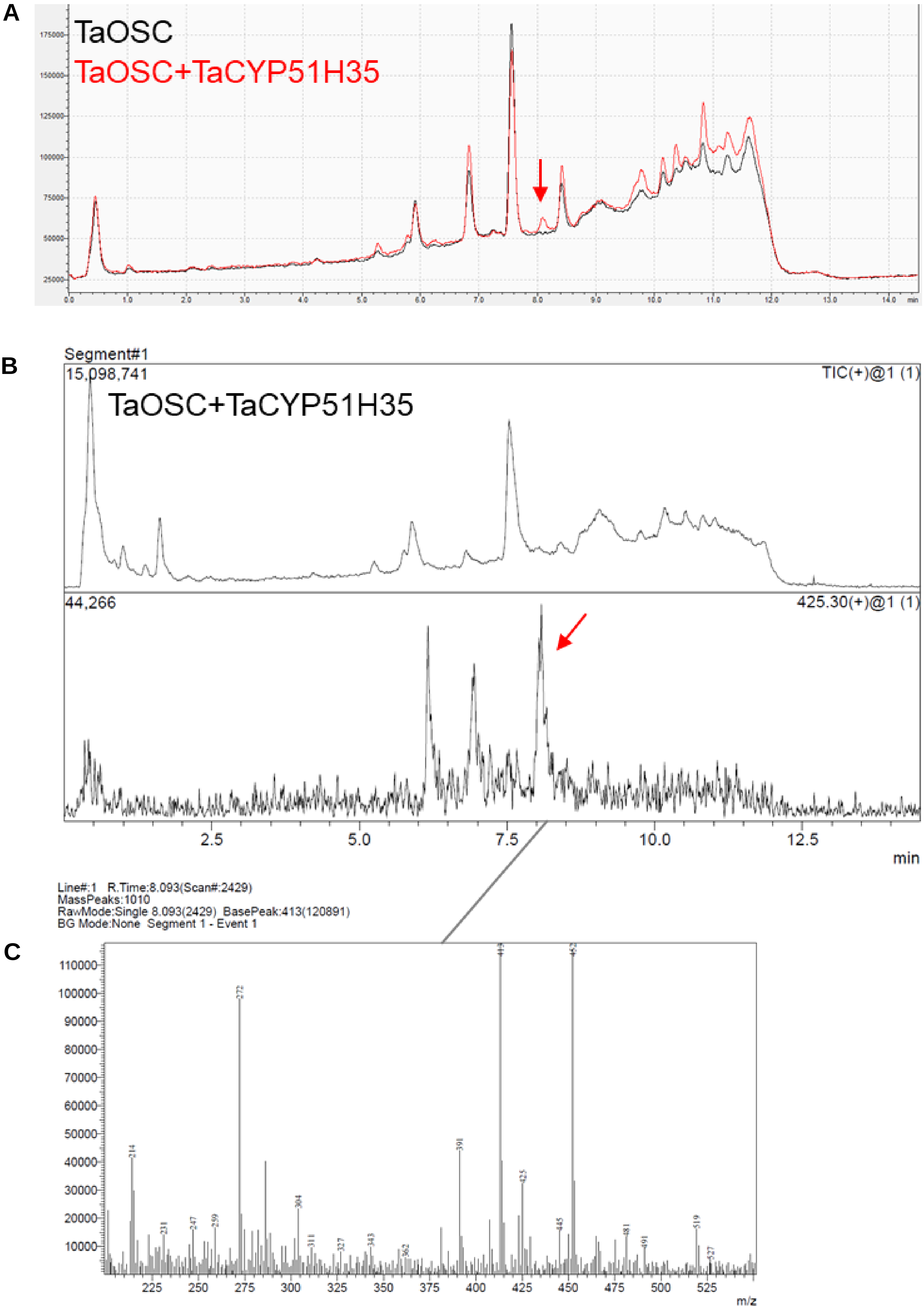

Fig. S7. LC-MS detection of 19-hydroxy-isoarborinol molecular ion. (A) CAD chromatogram. (B) MS chromatogram- TIC and EIC of 441.3. (C) Mass spectra at peak retention time.

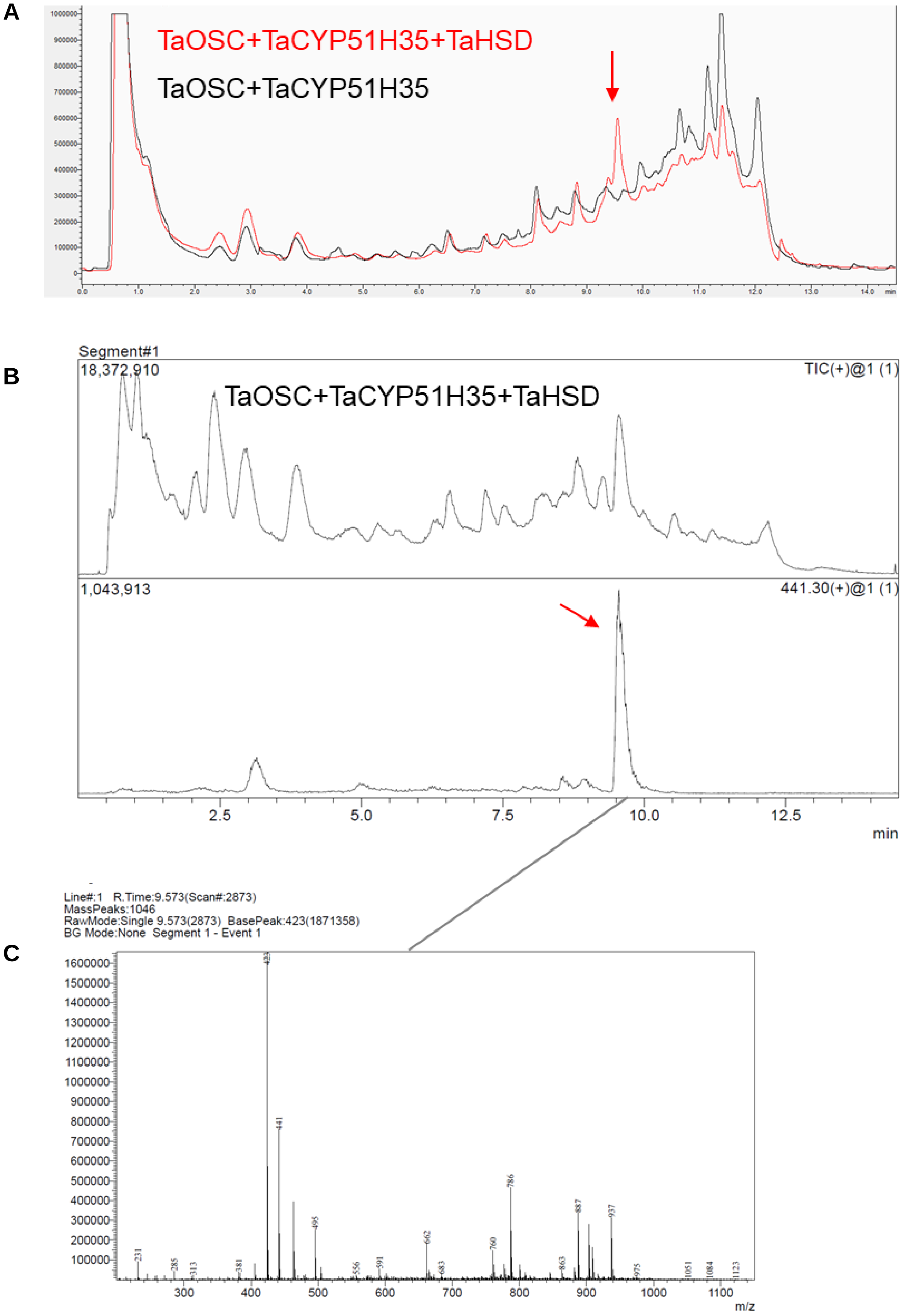

Fig. S8. LC-MS detection of 19-hydroxy-isoarborinone molecular ion. (A) CAD chromatogram. (B) MS chromatogram- TIC and EIC of 441.3. (C) Mass spectra at peak retention time.

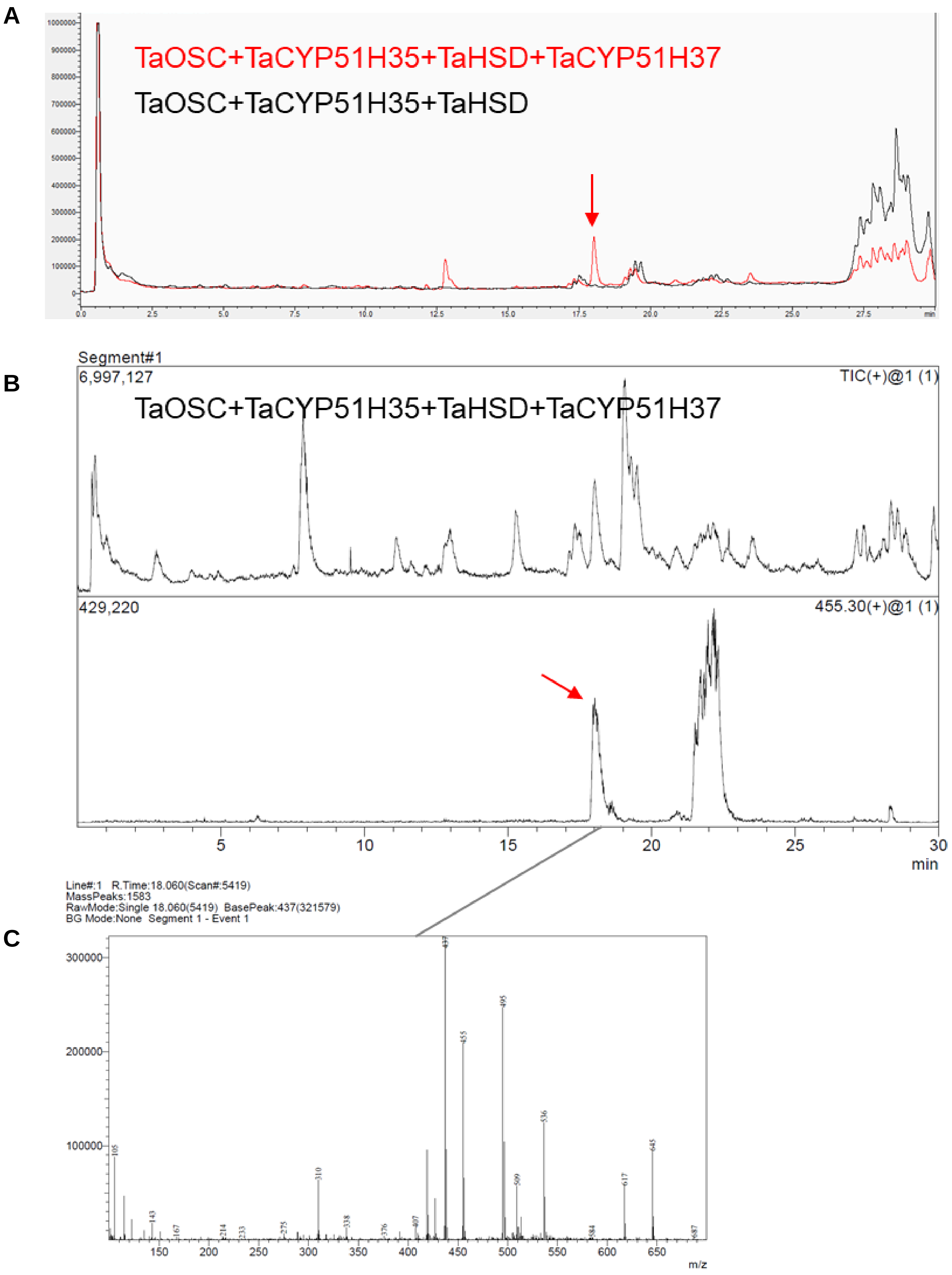

Fig. S9. LC-MS detection of ellarinacin molecular ion. (A) CAD chromatogram. (B) MS chromatogram- TIC and EIC of 455.3. (C) Mass spectra at peak retention time.

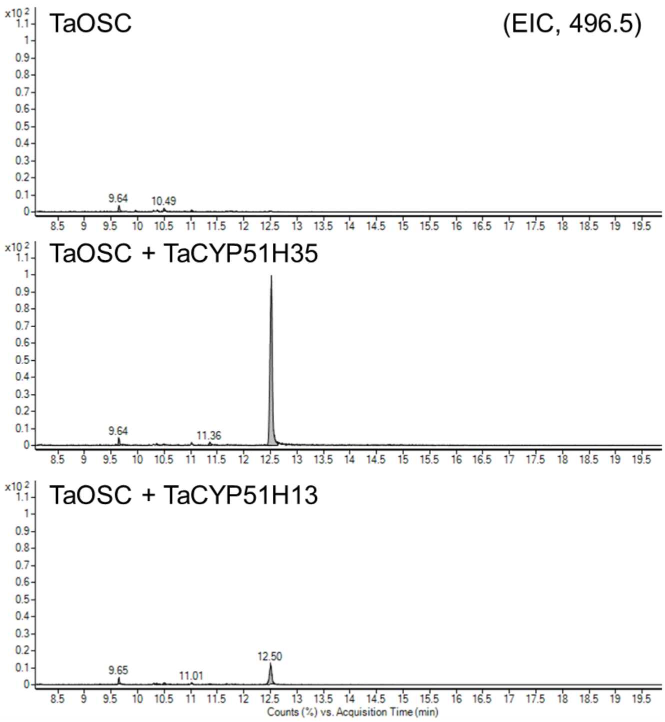

Fig. S10. GC-MS analysis of wheat TaCYP51H35 and TaCYP51H13 expression in *N. benthamiana.* EIC, extracted ion chromatogram of fragment ion representing 19-hydroxy-isoarborinol (496.5). Y-axes are linked. Oat tHMGR was included in all combinations of genes.

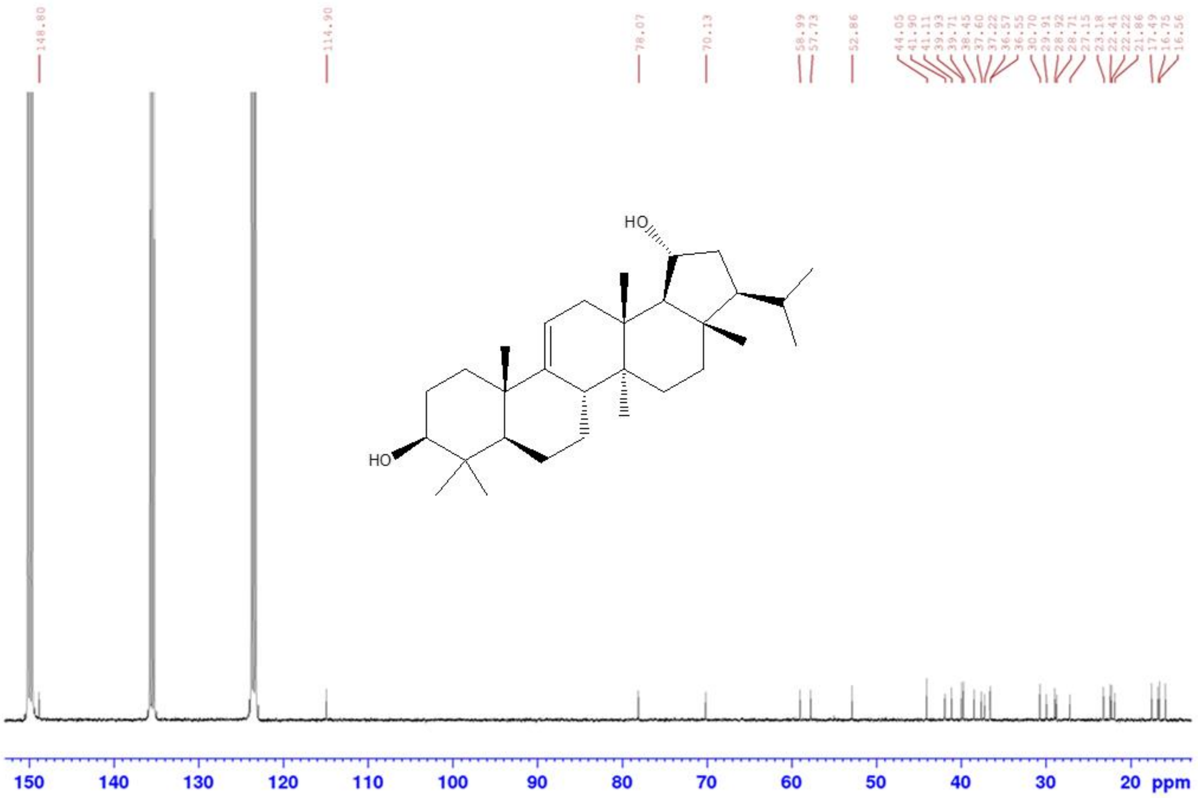

Fig. S11. ^{13}C **19-hydroxy-isoarborinol** (**Pyridine-d5)**. Referenced to the most downfield peak reported in the literature (33). 400 MHz instrument.

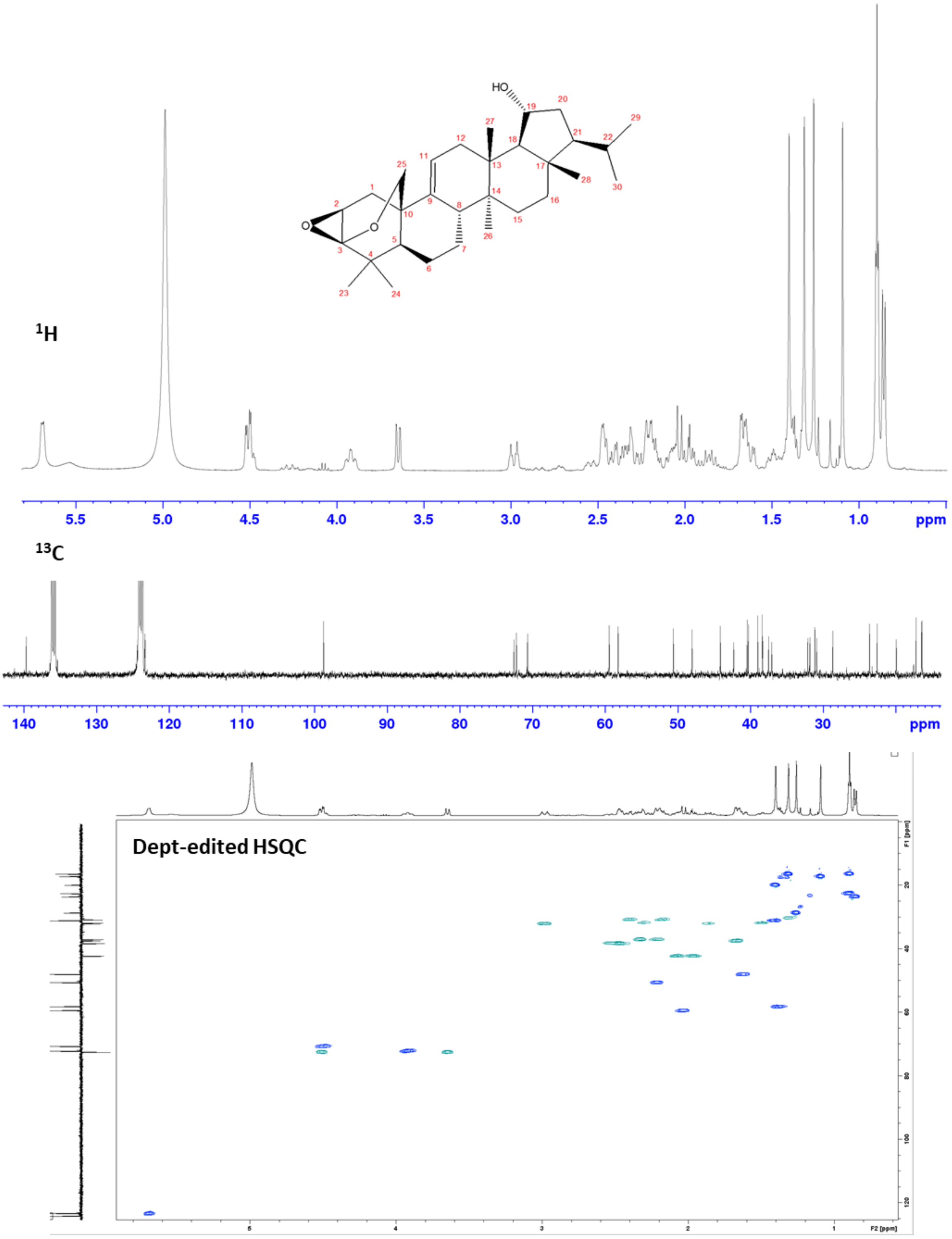

Fig. S12. ^{1}H, ^{13}C, and dept-edited HSQC spectra for ellarinacin, (pyridine-d5). Referenced to residual solvent peak (^{1}H δ: 8.74) (^{13}C δ: 150.3)]. 400 MHz instrument.

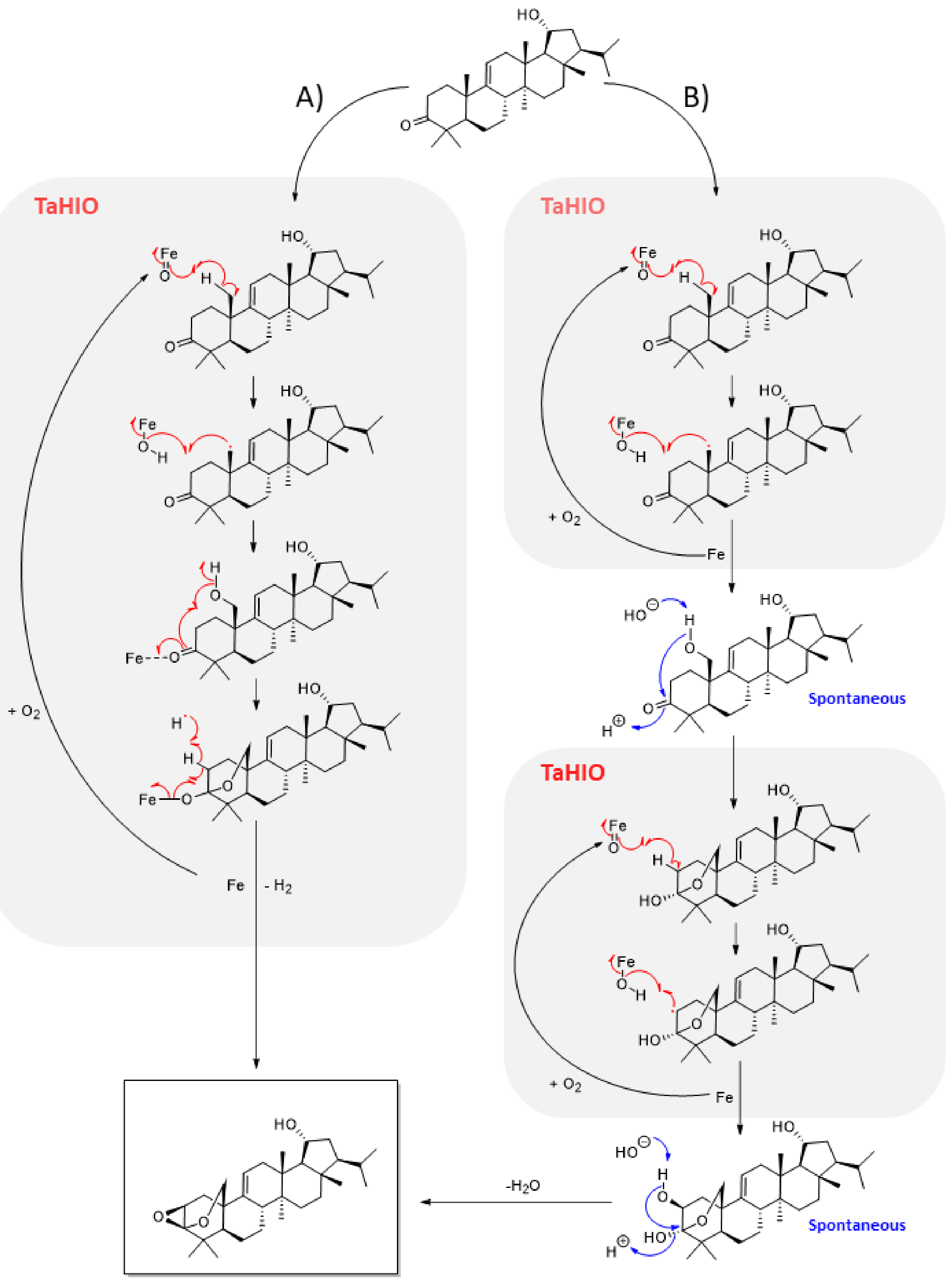

Fig. S13. Possible reaction mechanisms of TaHIO (TaCYP51H37). Reaction mechanism may involve one catalytic cycle (A) or alternatively involve two independent catalytic cycles (B).

		1.	2.	3.	4.	5.	6.	7.	8.	9.	10.	11.	12.
1.	AK067451|O.sativa|IAS	100	62.3	63.8	49.8	56.4	56.4	56.2	54.7	54.8	56.4	56.2	55.6
2.	AJ311789|A.strigosa|bAS	62.3	100	68	48.3	53.8	53.7	53.6	51.9	52.1	55.4	54.4	53.3
3.	AK070534|O.sativa|ABS	63.8	68	100	48.1	55.6	57.1	57.1	53.3	53.4	57.2	56.9	56.6
4.	LOC4344966|O.sativa|PTS	49.8	48.3	48.1	100	56.1	55.3	55.5	53.9	53.2	56.1	54.9	54.4
5.	Bradi3g22802|B.distachyon|IAS	56.4	53.8	55.6	56.1	100	90.4	88.3	63	62.6	71.5	69.8	69.6
6.	AS01G014480|A.strigosa|IAS	56.4	53.7	57.1	55.3	90.4	100	91.3	63.5	63	73.5	71.2	71.5
7.	TraesCS5D02G011800|T.aestivum|IAS	56.2	53.6	57.1	55.5	88.3	91.3	100	62.5	62.1	71.6	69.5	69.8
8.	AK066327|O.sativa|PS	54.7	51.9	53.3	53.9	63	63.5	62.5	100	95.7	71.5	69	67.6
9.	AYV65354|O.sativa|ORS	54.8	52.1	53.4	53.2	62.6	63	62.1	95.7	100	70.3	68.1	67.1
10.	AK121211|O.sativa|CAS	56.4	55.4	57.2	56.1	71.5	73.5	71.6	71.5	70.3	100	87.2	86.4
11.	AJ311790|A.strigosa|CAS	56.2	54.4	56.9	54.9	69.8	71.2	69.5	69	68.1	87.2	100	92.2
12.	TraesCS6D02G099400|T.aestivum|CAS*	55.6	53.3	56.6	54.4	69.6	71.5	69.8	67.6	67.1	86.4	92.2	100

Fig. S14. Percent identity matrix of amino acid sequences of characterised Poaceae cycloartenol- and triterpene- synthases. Sequence alignment and percent identity matrix was generated with Clustal Omega v2.1. IAS, isoarborinol synthase; bAS, beta-amyrin synthase; ABS, achilleol b synthase; PTS, poaceaetapetol synthase; PS, parkeol synthase; ORS, orysatinol synthase; CAS, cycloartenol synthase. Asterisk denotes a predicted *T. aestivum* CAS annotation, based on phylogeny and a constitutive expression pattern in wheat RNA-seq data.

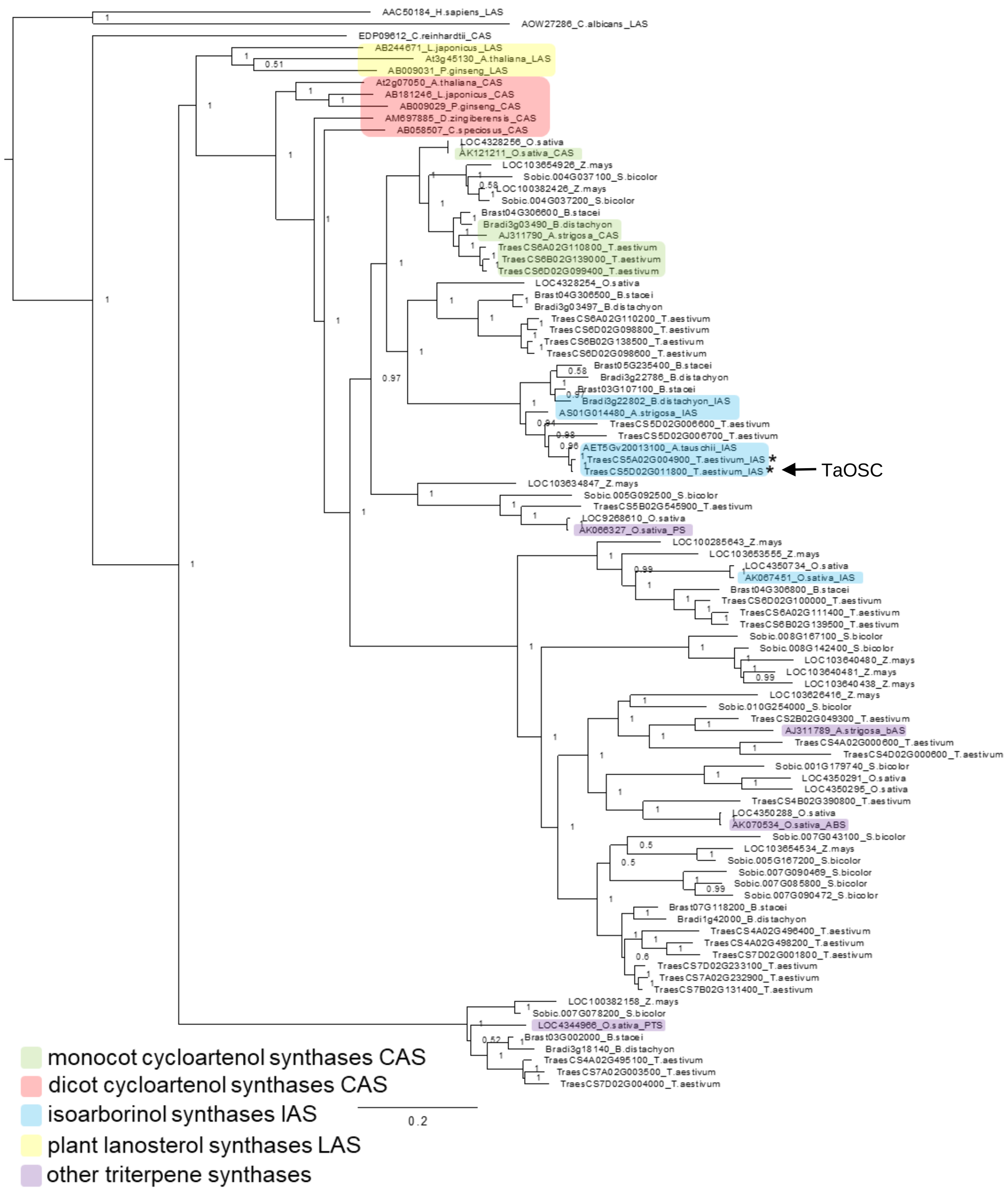

Fig. S15. phylogeny of predicted oxidosqualene cyclases from wheat, together with selected monocot and dicot OSCs. Bayesian tree comprised of protein sequences of characterized and non-characterized monocot OSCs and characterized dicot cycloartenol and lanosterol synthases. Cycloartenol synthase from *Chlamydomonas reinhardtii* and lanosterol synthases from *Homo sapiens* and *Candida albicans* are included as outgroups. Sequences were aligned with MUSCLE (with a maximum of 100 iterations) and a phylogenetic tree was generated using MrBayes (34), with a mixed amino acid probability model and default MCMC parameters except 0.7 temperature. TaOSC sequences from 3(5D) and 3(5A) clusters are asterisked. *T. aestivum* and *B. distachyon* CAS annotations are predicted, based on phylogeny and constitutive expression patterns in RNA-seq data. CAS, cycloartenol synthase; IAS, isoarborinol synthase; LAS, lanosterol synthase; bAS, beta-amyrin synthase; PS, parkeol synthase; ABS, achilleol B synthase; PTS, poaceatapetol synthase.

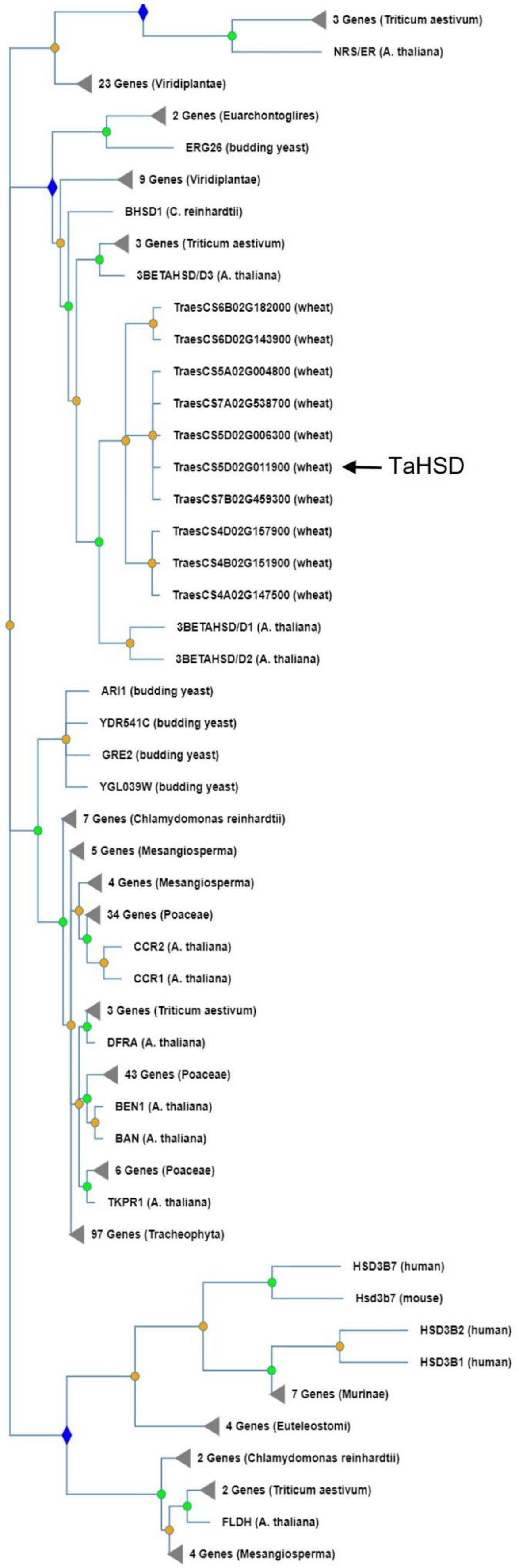

Fig. S16. Phylogenetic tree of selected members of the NAD dependent epimerase/dehydratase family (Panther family PTHR10366). The tree was constructed in PhyloGenes v2.0 (http://www.phylogenes.org/tree/PTHR10366). TaHSD is clustered with 3βHSD/D1 and 3βHSD/D2 that are take part in sterol biosynthesis in *Arabidopsis thaliana* (35).

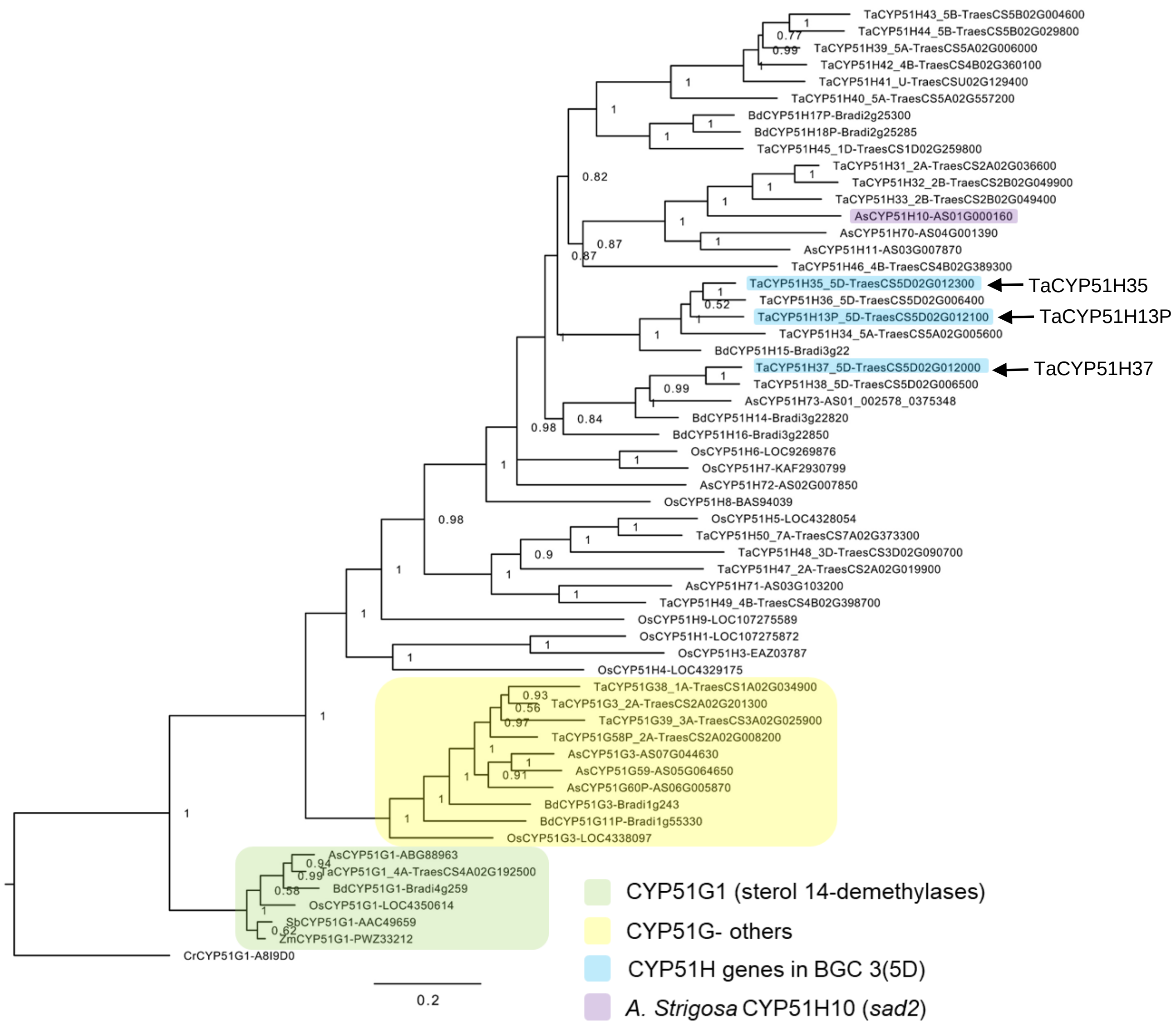

Fig. S17. Phylogeny of CYP51-family cytochrome P450 proteins identified in wheat, brachypodium, oat and rice. Peptide sequences of CYP51s were aligned with MUSCLE (with a maximum of 100 iterations) and a phylogenetic tree was generated using MrBayes (34), with a mixed amino acid probability model and default MCMC parameters. The tree includes one representative homoeolog of each assigned wheat CYP51. For a tree including all identified wheat homoeologs, see Fig. 18. Ta, *Triticum aestivum*; Bd, *Brachypodium distachyon*; Os, *Oryza sativa*; As, *Avena strigosa*; Zm, *Zea mays*; Sb, *Sorghum bicolor*; Cr, *Chlamydomonas reinhardtii*.

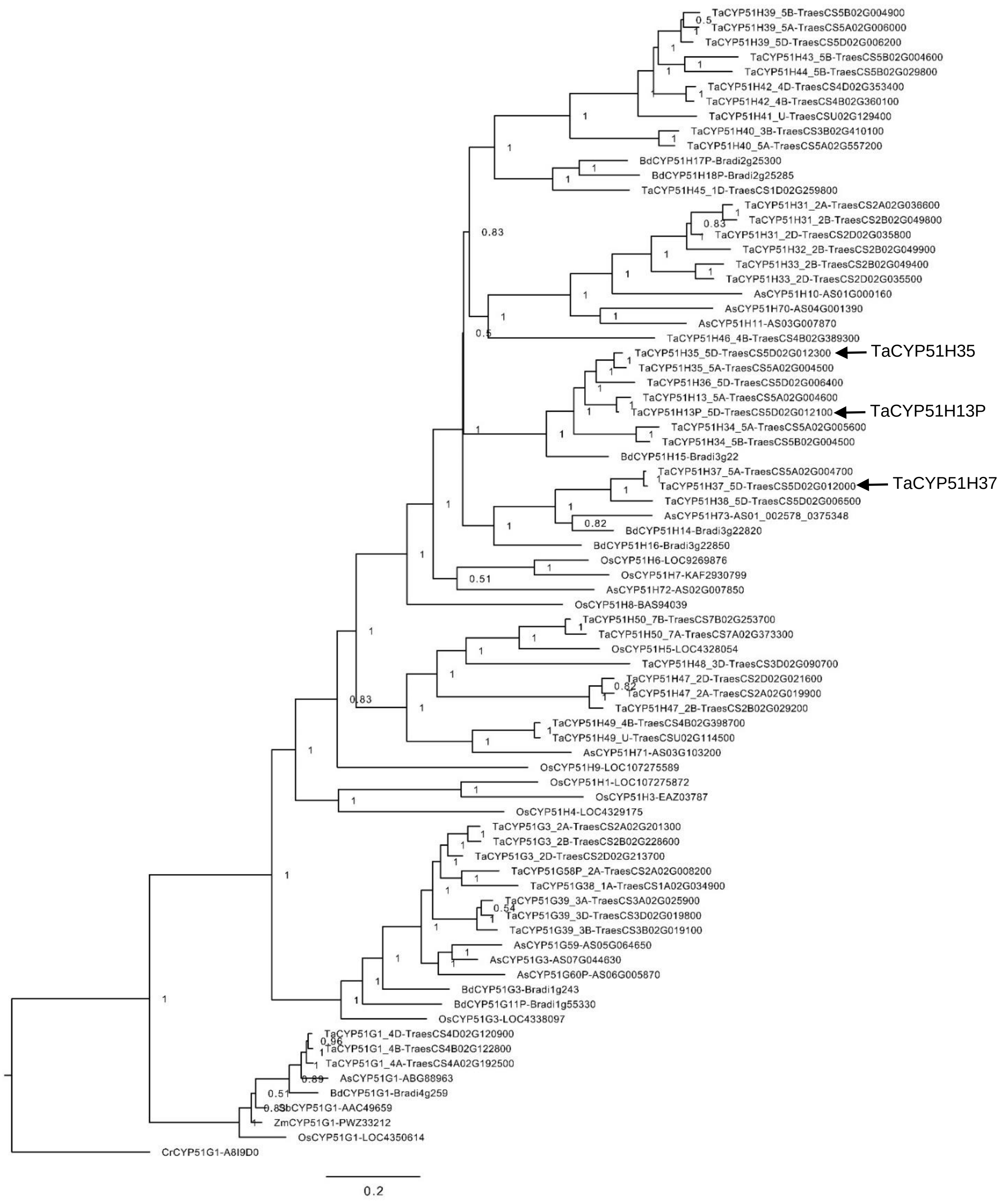

Fig. S18. Bayesian tree of CYP51 genes from wheat, brachypodium, oat and rice.

A

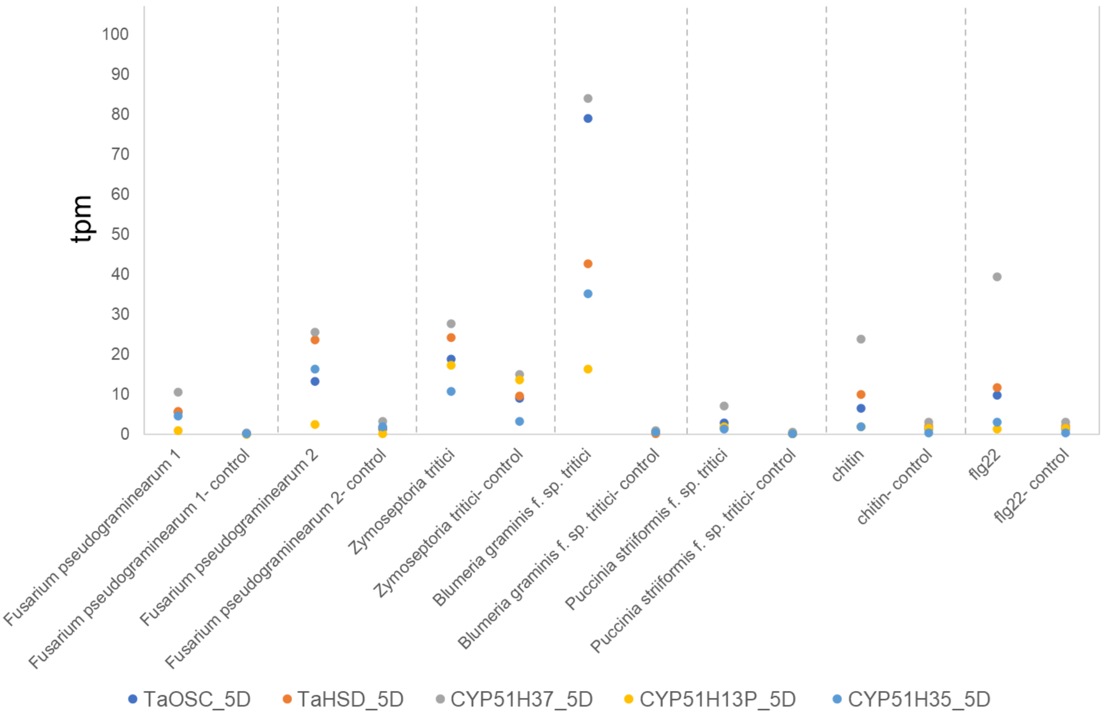

B

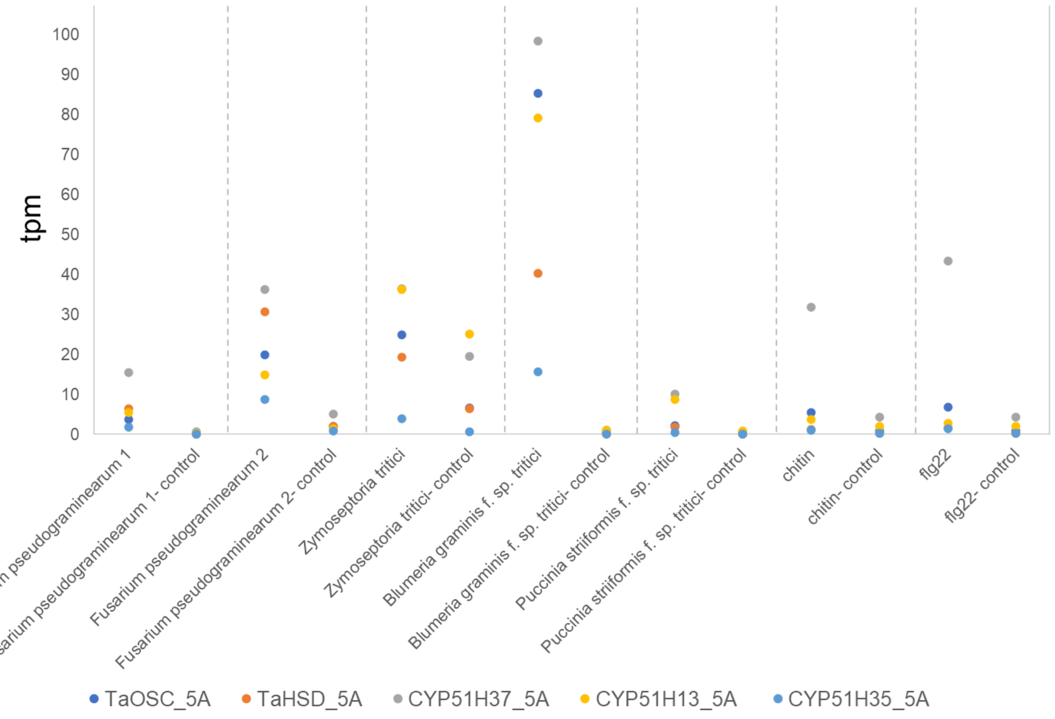

Fig. S19. Expression of wheat clustered genes under biotic stress. (A) Genes in Chr.5D cluster. (B) genes in Chr.5A cluster. Expression data extracted from http://www.wheat-expression.com includes studies applying treatment with *Fusarium pseudograminearum* (study 1: (36), study 2: (37)), *Zymoseptoria tritici* (38), *Blumeria graminis* (39), *Puccinia striiformis* (40), chitin (14), or flg22 (14). TaCYP51H13P_5D values are average of TraesCS5D01G012100 and TraesCS5D01G012200. tpm, transcripts per million.

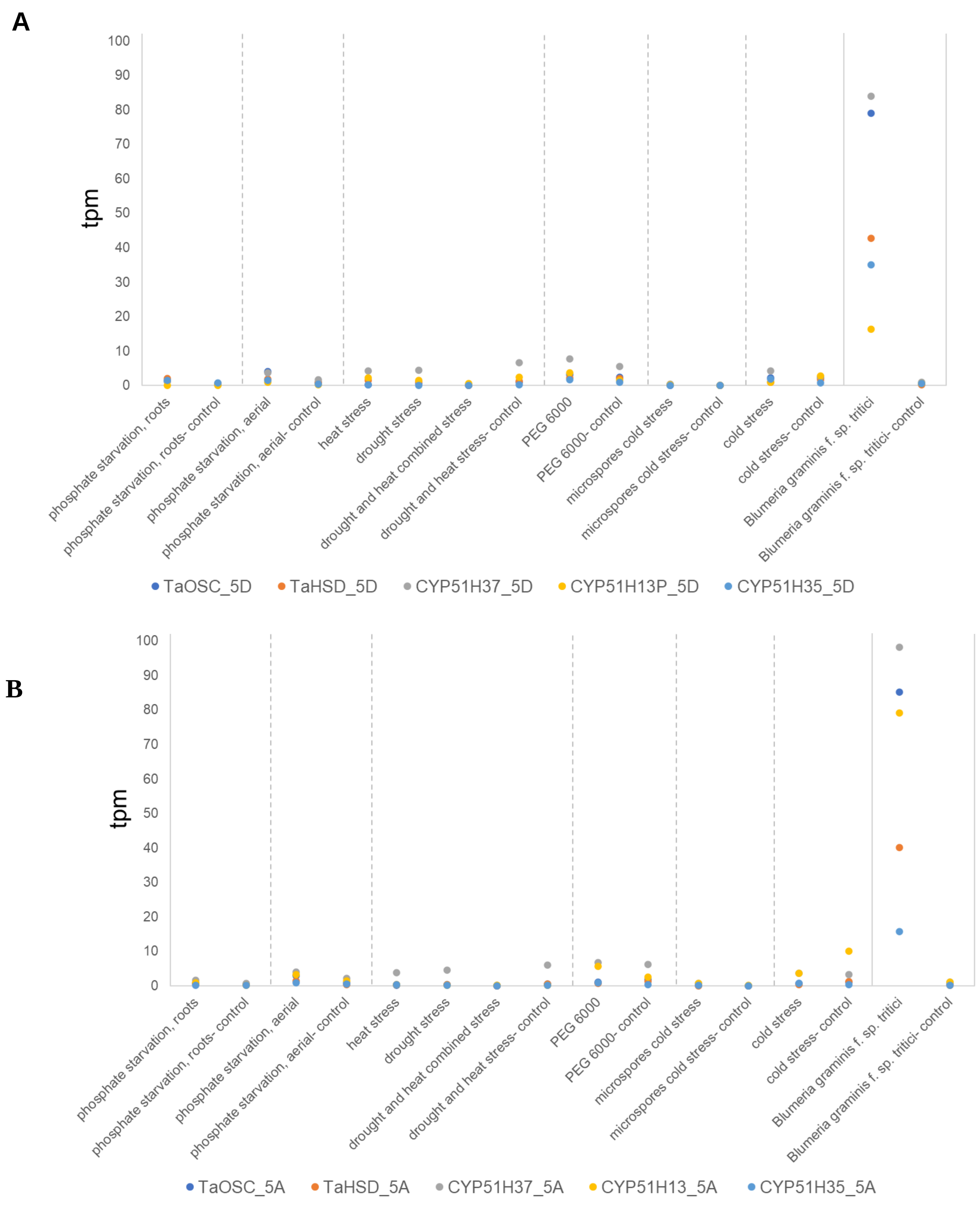

Fig. S20. Expression of wheat clustered genes under abiotic stress. (A) Genes in Chr.5D cluster. (B) Genes in Chr.5A cluster. Expression data extracted from http://www.wheat-expression.com includes studies applying phosphate starvation (41), heat and drought stress (42), PEG 6000, microspores cold stress (43) and cold stress (44). TaCYP51H13P_5D values are average of TraesCS5D01G012100 and TraesCS5D01G012200. Data of powdery mildew infection experiment (*Blumeria graminis f. sp. tritici*) (39) is shown for comparison. tpm, transcripts per million.

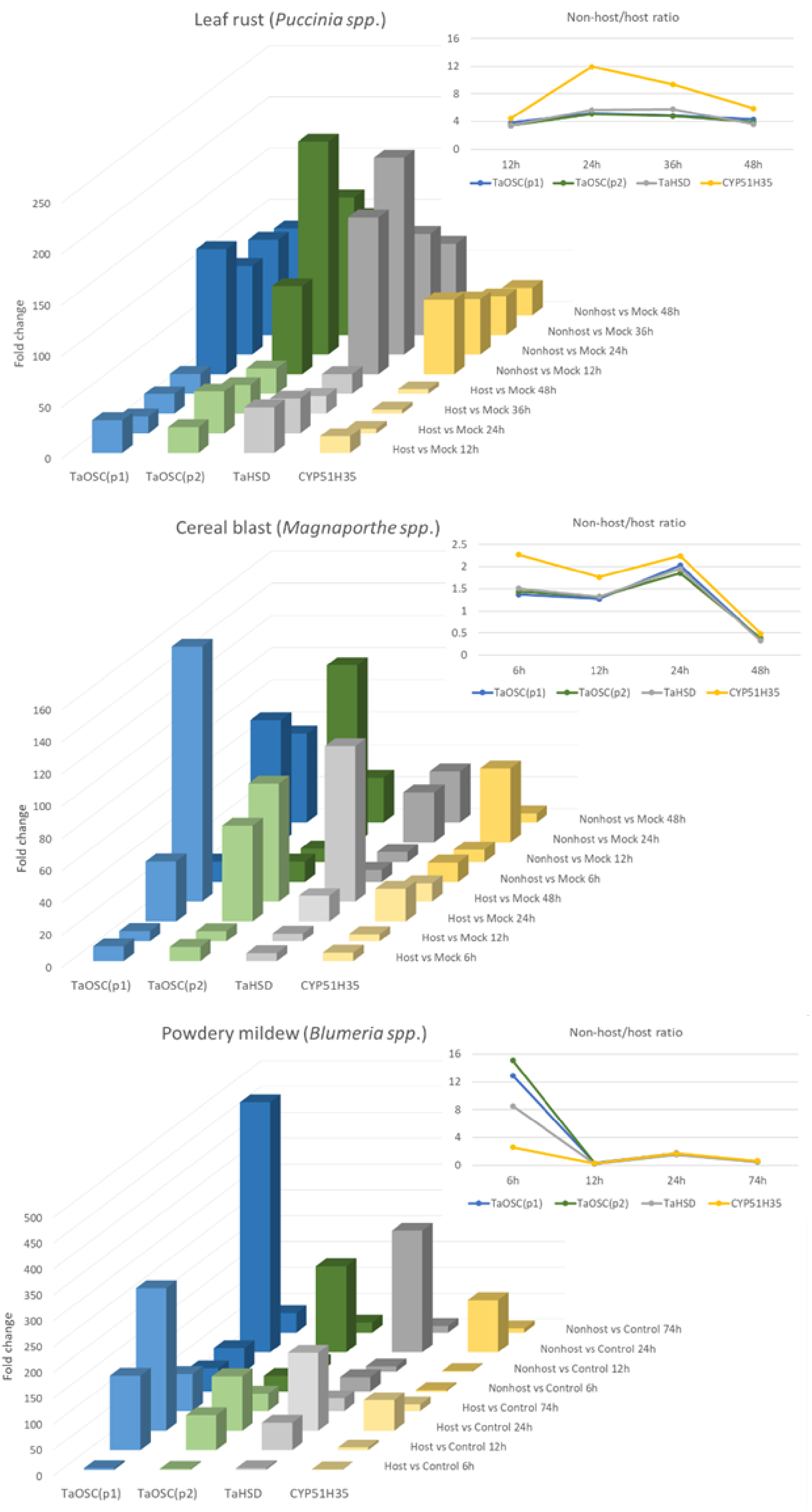

Fig. S21. Wheat clustered genes TaOSC, TaHSD and TaCYP51H35 are induced by infection of wheat plants with adapted and non-adapted isolates of leaf rust, cereal blast, or powdery mildew. Microarray probe signal intensities are plotted in 3D-columns showing fold change expression in adapted and non-adapted vs. control experiments. 2D plots show fold change ratio of non-adapted/adapted vs. control, per each time point. TaOSC is represented by two probes.

A

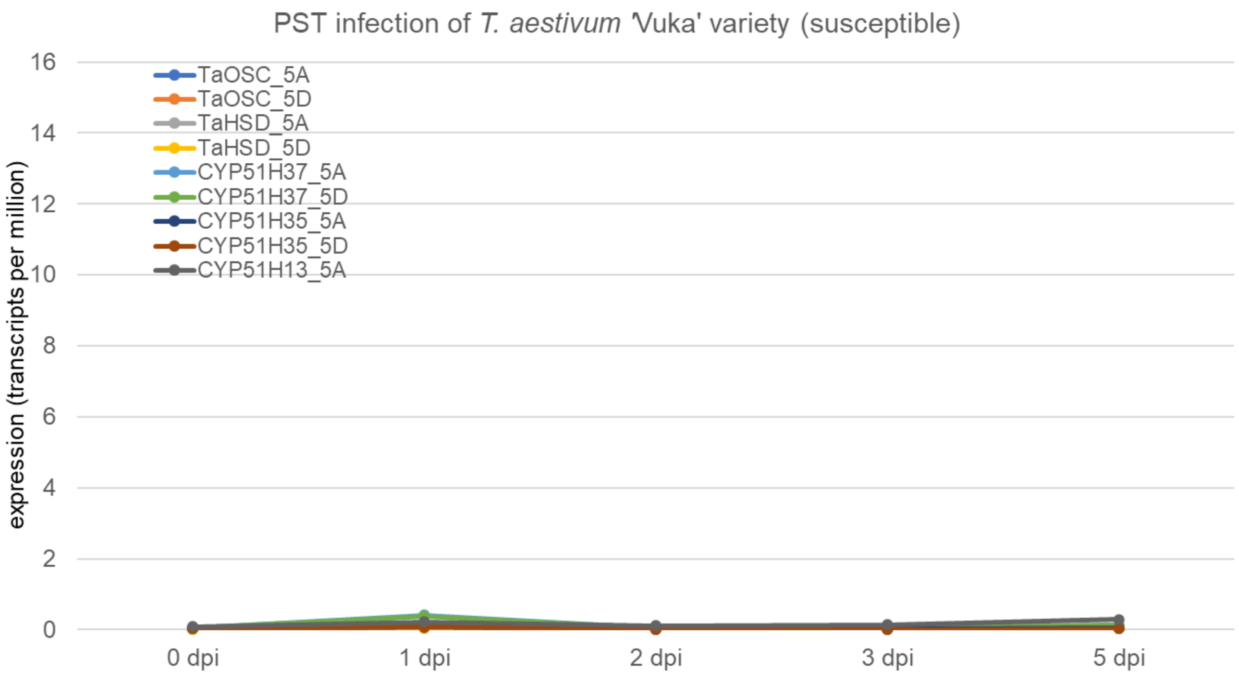

B

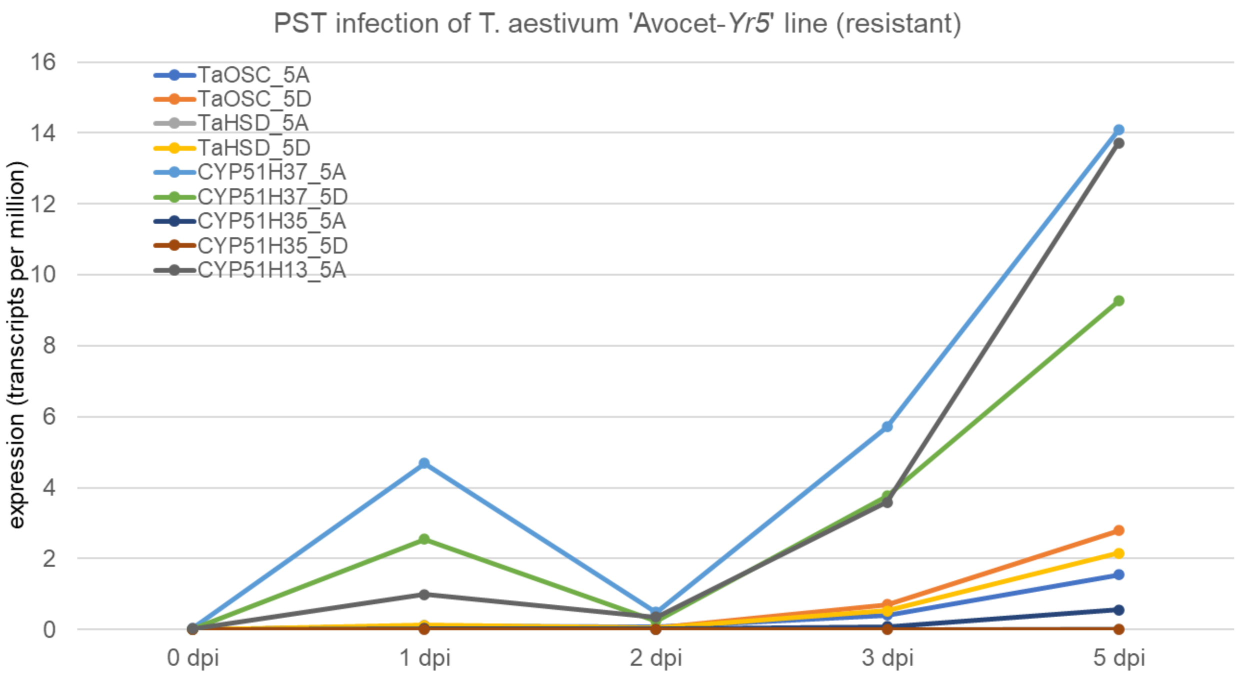

Fig. S22. Induction of wheat cluster genes following infection with yellow rust (*Puccinia striiformis f. sp. tritici*) is suppressed in a susceptible variety. Expression values were extracted from RNA-seq data of a susceptible wheat variety 'Vuka' (A), or a resistant 'Avocet' introgression line (B), infected with yellow rust, 0, 1, 2, 3, and 5 days post infection.

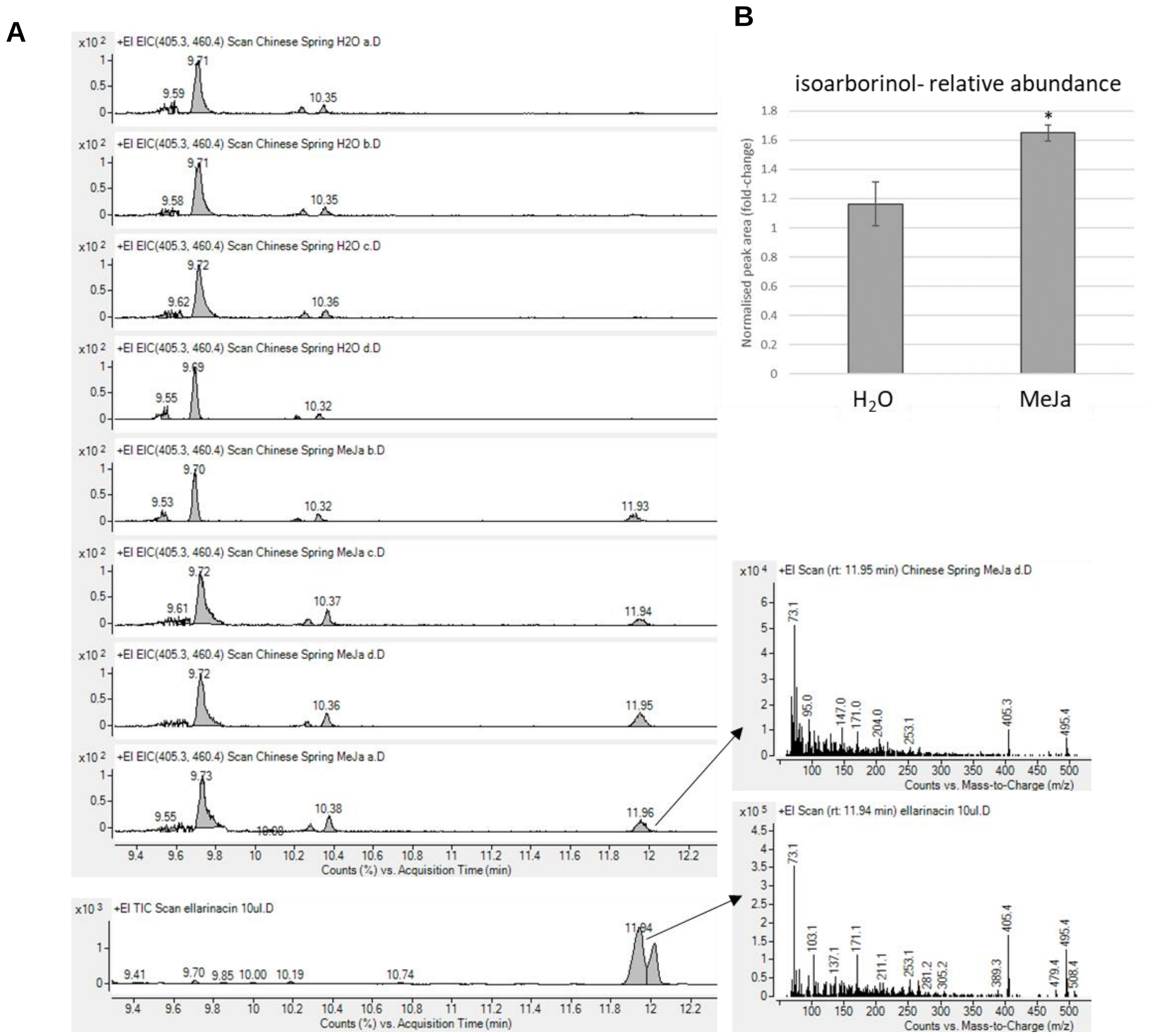

Fig. S23. GC-MS analysis of TMS-derivatized extracts from wheat leaves treated with methyl jasmonate (MeJa), or H_2O (control). (A) Extracted ion chromatograms for ions representing ellarinacin (405.3, Rt 11.94) and 5α-cholestan-3β-ol (460.4, Rt 9.70) are shown for extracts from four biological replicates, together with mass spectra of ellarinacin peak at Rt 11.94. Y-axes are linked to peak of internal standard- 5α-cholestan-3β-ol. Wheat leaf extracts were compared to ellarinacin purified from *N. benthamiana* for identification. (B) Relative abundance of isoarborinol in TMS-derivatized extracts of MeJa or H_2O-treated wheat leaves, based on GC-MS analysis of four biological replicates.

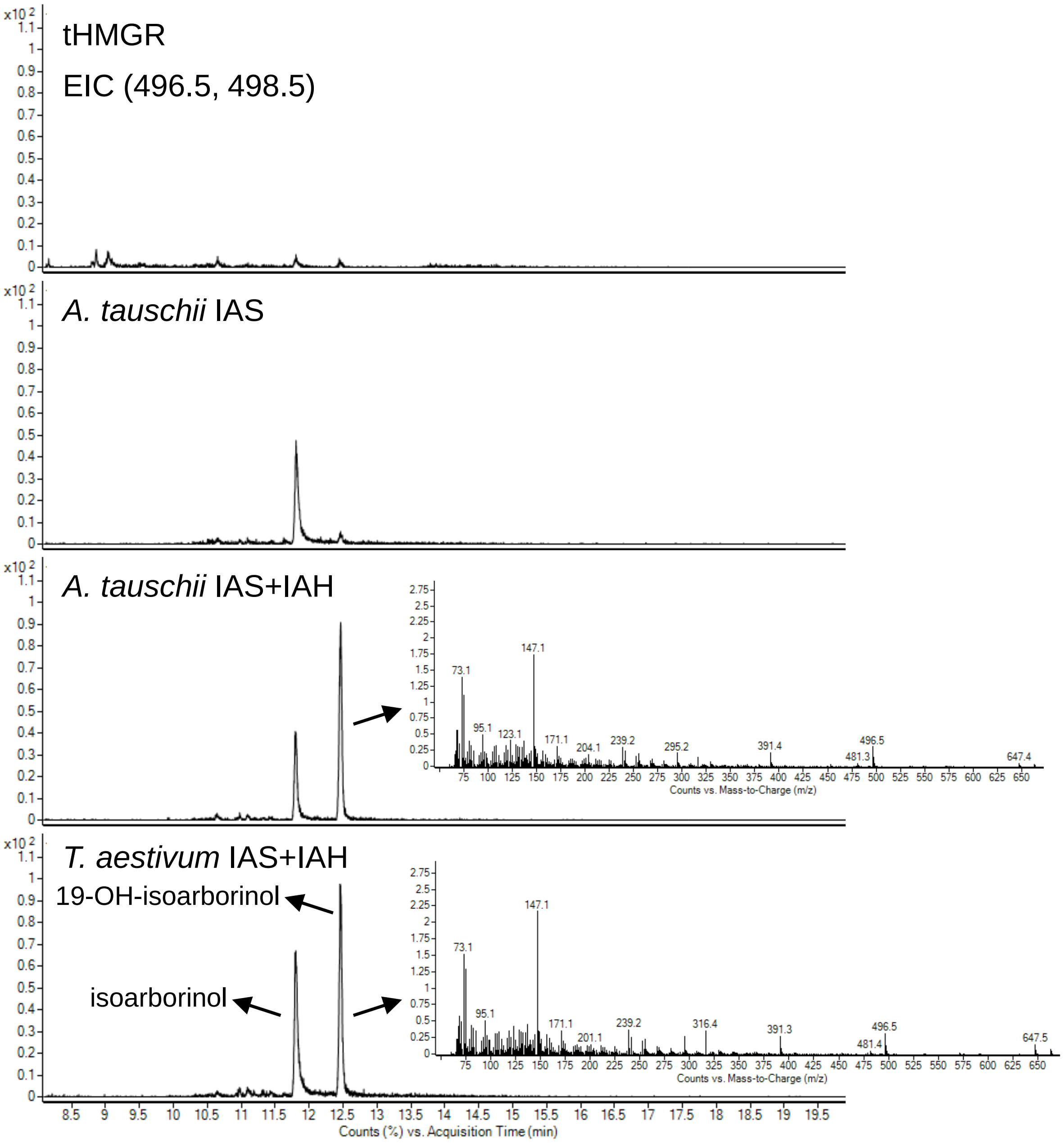

Fig. S24. GC-MS analysis of *Aegilops tauschii* IAS and IAH expression in *N. benthamiana.* EIC, extracted ion chromatogram of fragment ions representing isoarborinol (498.5) and 19-hydroxy-isoarborinol (496.5). Analysis of wheat IAS and IAH expression is provided for reference.

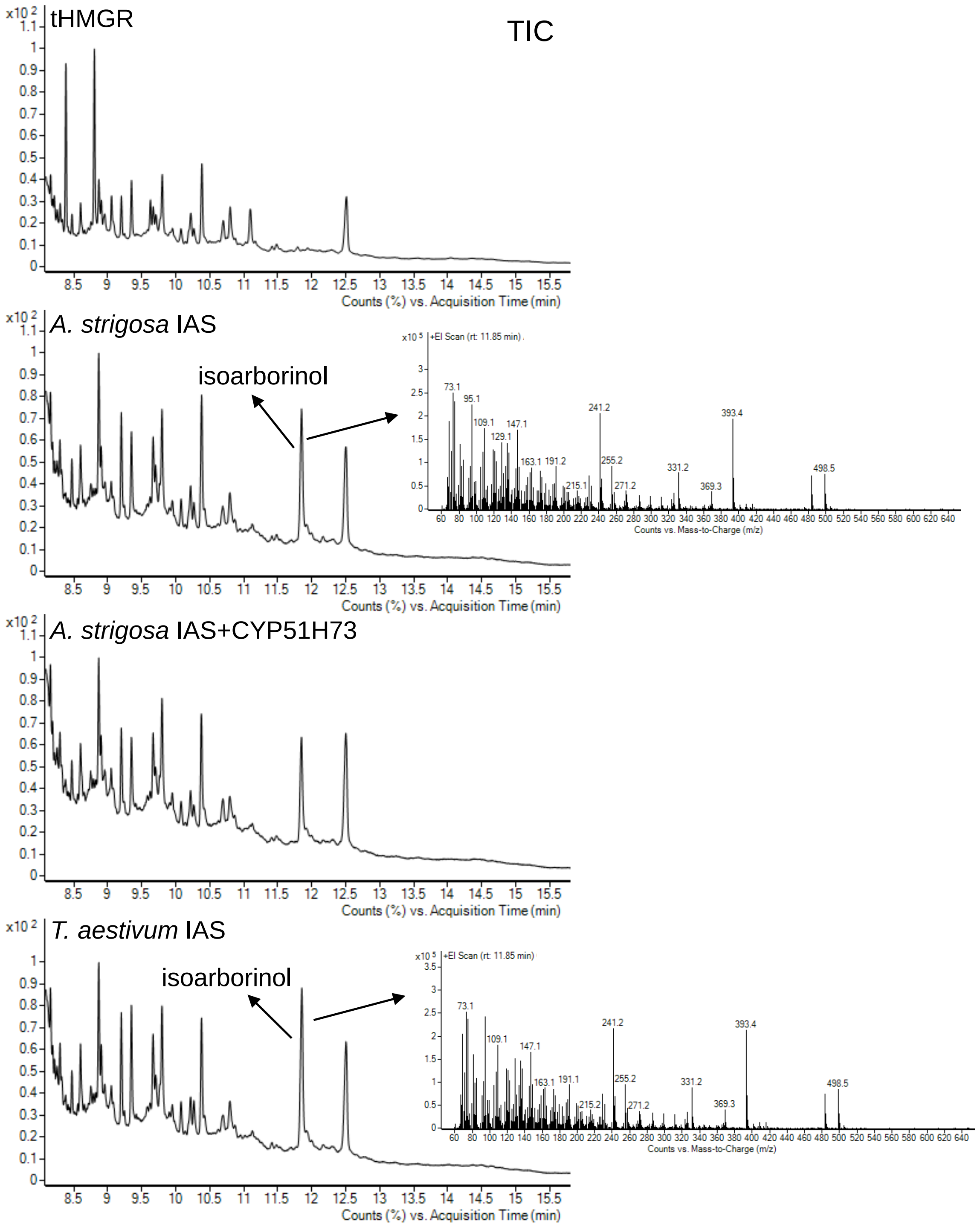

Fig. S25. GC-MS analysis of *Avena strigosa* IAS and CYP51H73 expression in *N. benthamiana*. TIC, total ion chromatogram. Analysis of wheat IAS expression is provided for reference.

A

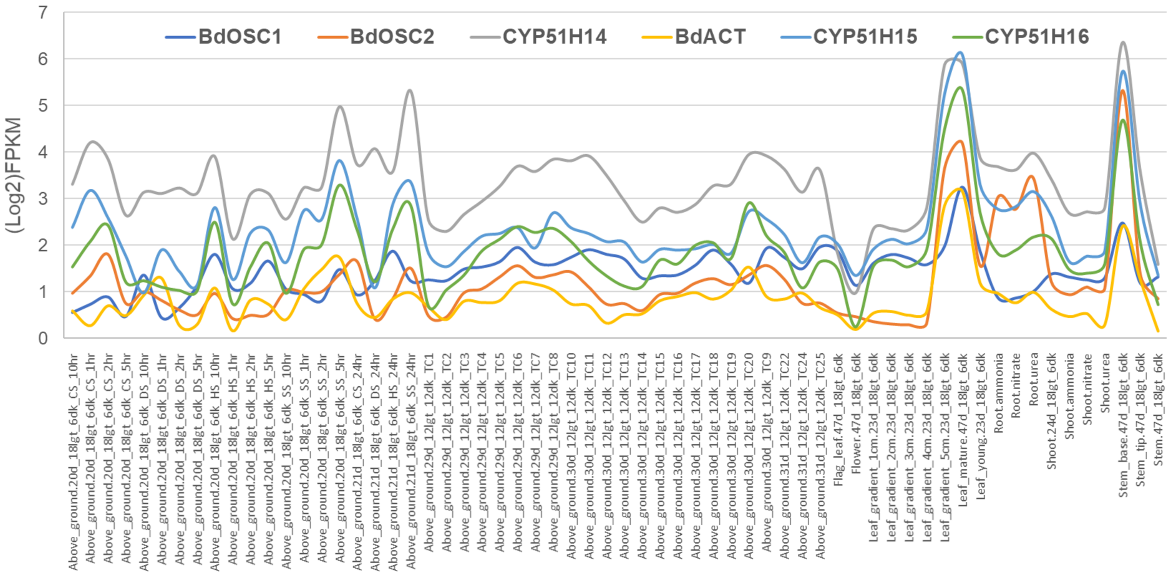

B

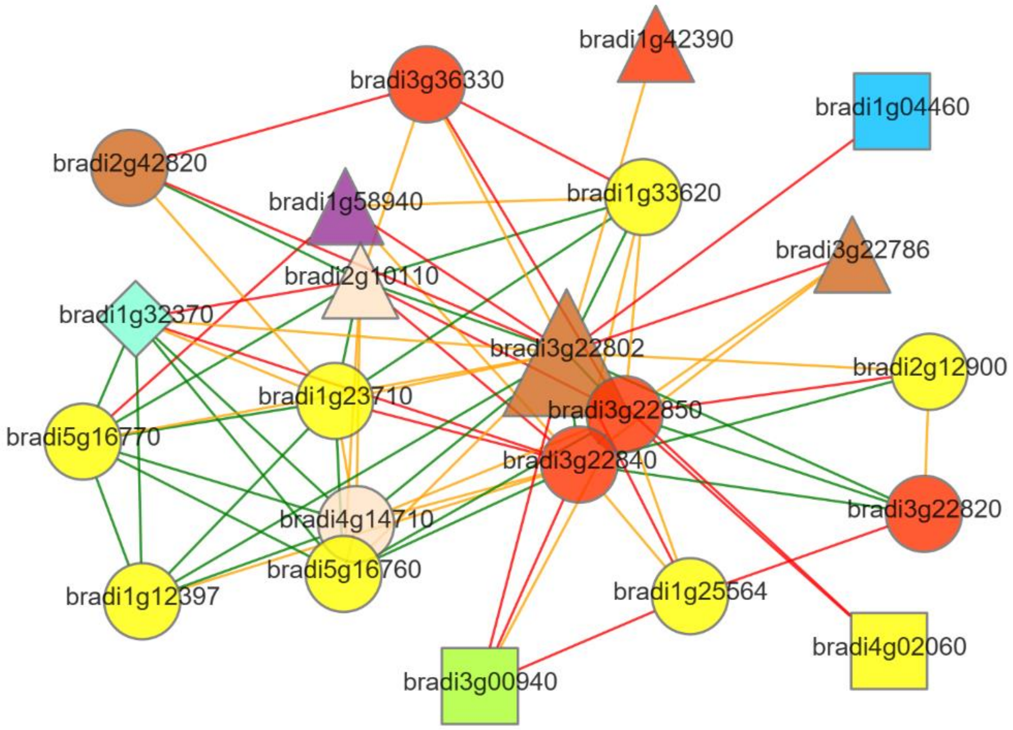

Fig. S26. Triterpene-related clustered genes on *B. distachyon* Chr.03 are co-expressed. (A) Gene expression data extracted from JGI Phytozome Gene Atlas (release 1.0), plotted as Log2 of FPKM values. (B) Co-expression network (1st level) neighbourhood of bradi3g22802 (BdOSC2) extracted from PlaNet (http://www.gene2function.de/), shows it is co-expressed with cluster genes: Bradi3g22786, Bradi3g22820, Bradi3g22840 and Bradi3g22850.

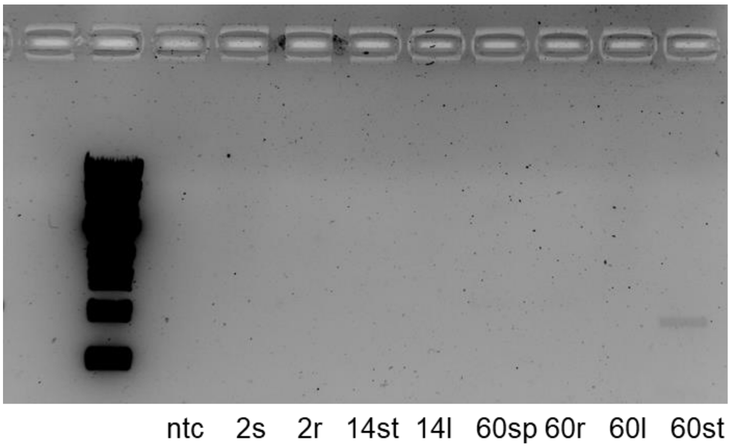

Fig. S27. Semiquantitative RT-PCR of Bradi3g22830. Amplification of Bradi3g22830 from *B. distachyon* mature stem base cDNA is observed only with prolonged camera exposure (8 sec.). ntc, no template control; 2s, seedling shoot (2 day old); 2r, seedling root; 14st, young plant stem base (14 day old); 14l, young plant leaf; 60sp, mature plant spike (60 day old); 60r, mature plant root; 60l, mature plant leaf; 60st, mature plant stem base.

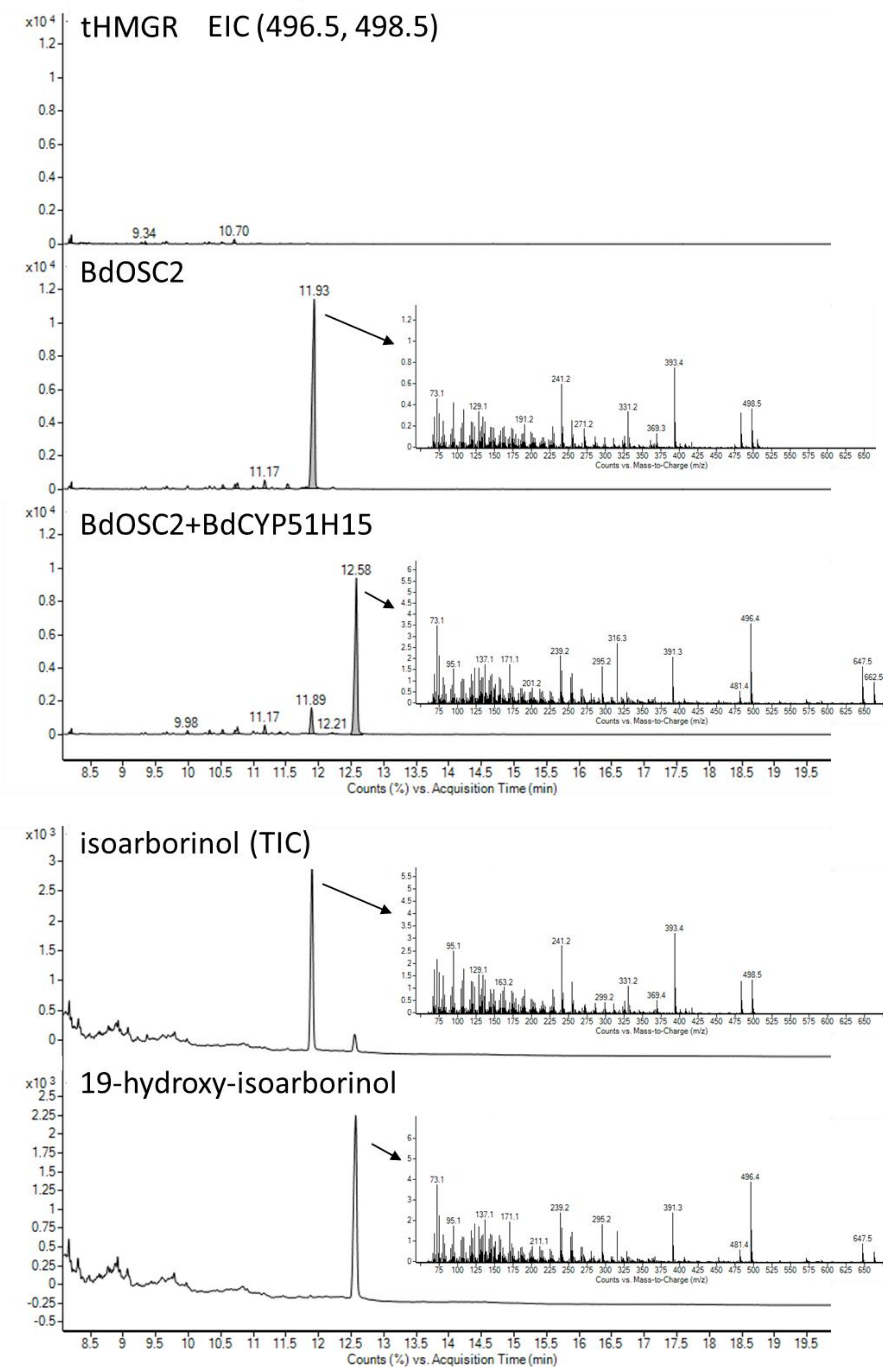

Fig. S28. GC-MS analysis of *B. distachyon* genes BdOSC2 and BdCYP51H15 expression in *N. benthamiana*. Extracted ion chromatograms for ions representing isoarborinol (498.5, Rt 11.93), and 19-hydroxy-isoarborinol brachynacin (496.5, Rt 12.58) and mass spectra at retention times of product peaks are shown. All gene expression combinations included oat tHMGR. Products were identified based on comparison to isoarborinol and 19-hydroxy-isoarborinol purified from *N. benthamiana*.

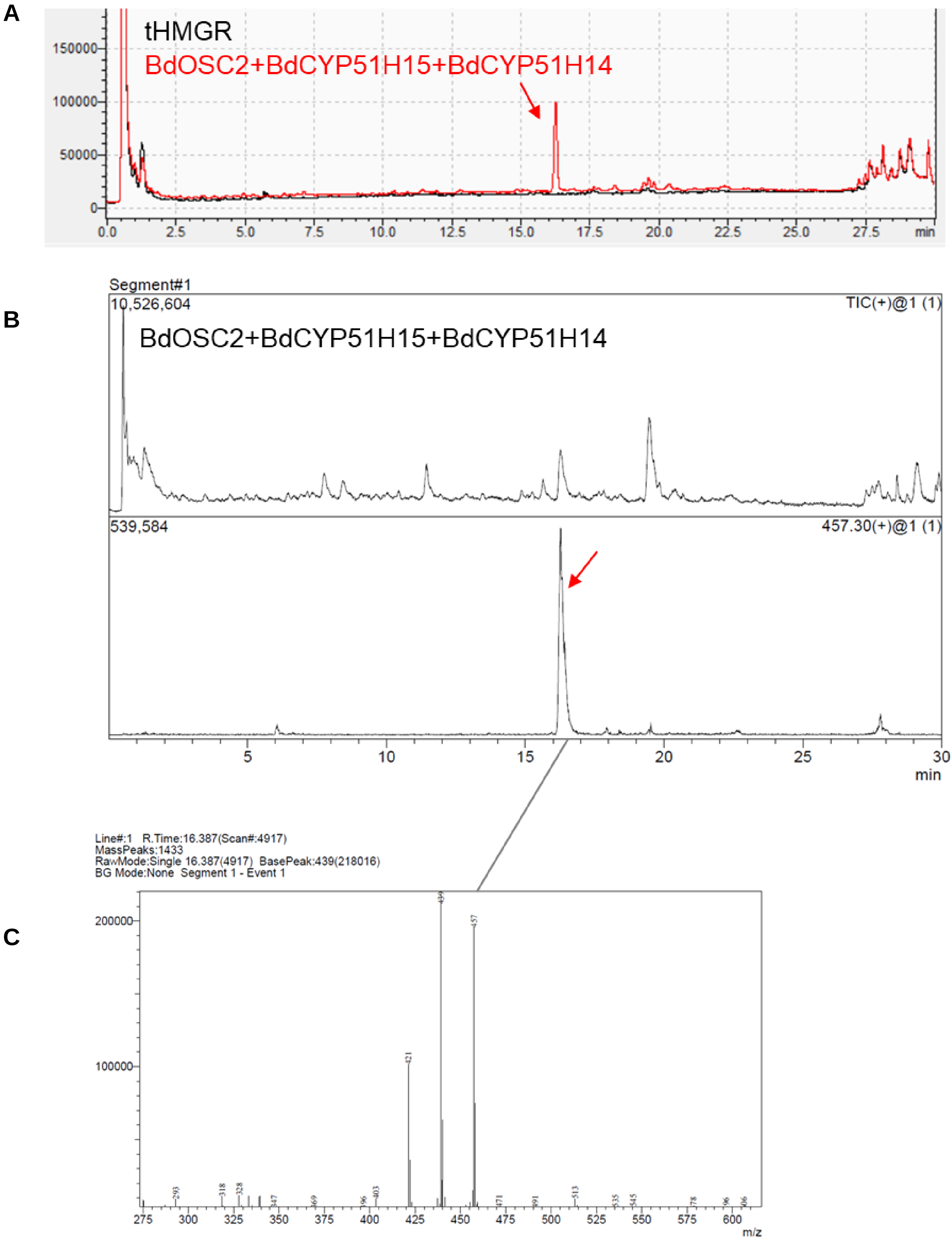

Fig. S29. LC-MS detection of 7,19,28-trihydroxy-isoarborinol molecular ion. (A) CAD chromatogram. (B) MS chromatogram- TIC and EIC of 457.3. (C) mass spectra at peak retention time.

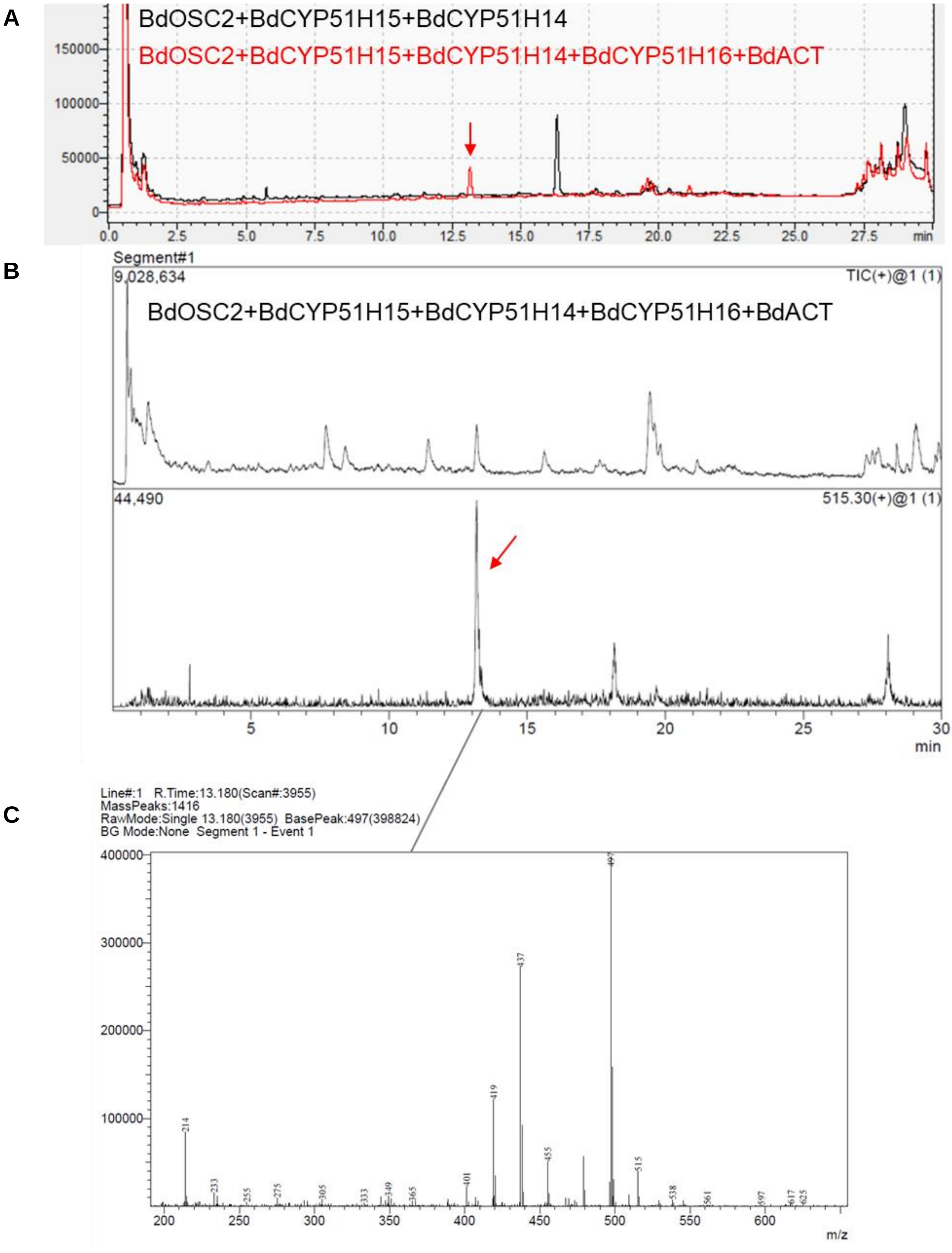

Fig. S30. LC-MS detection of brachynacin molecular ion. (A) CAD chromatogram, (B) MS chromatogram- TIC and EIC of 515.3. (C) mass spectra at peak retention time.

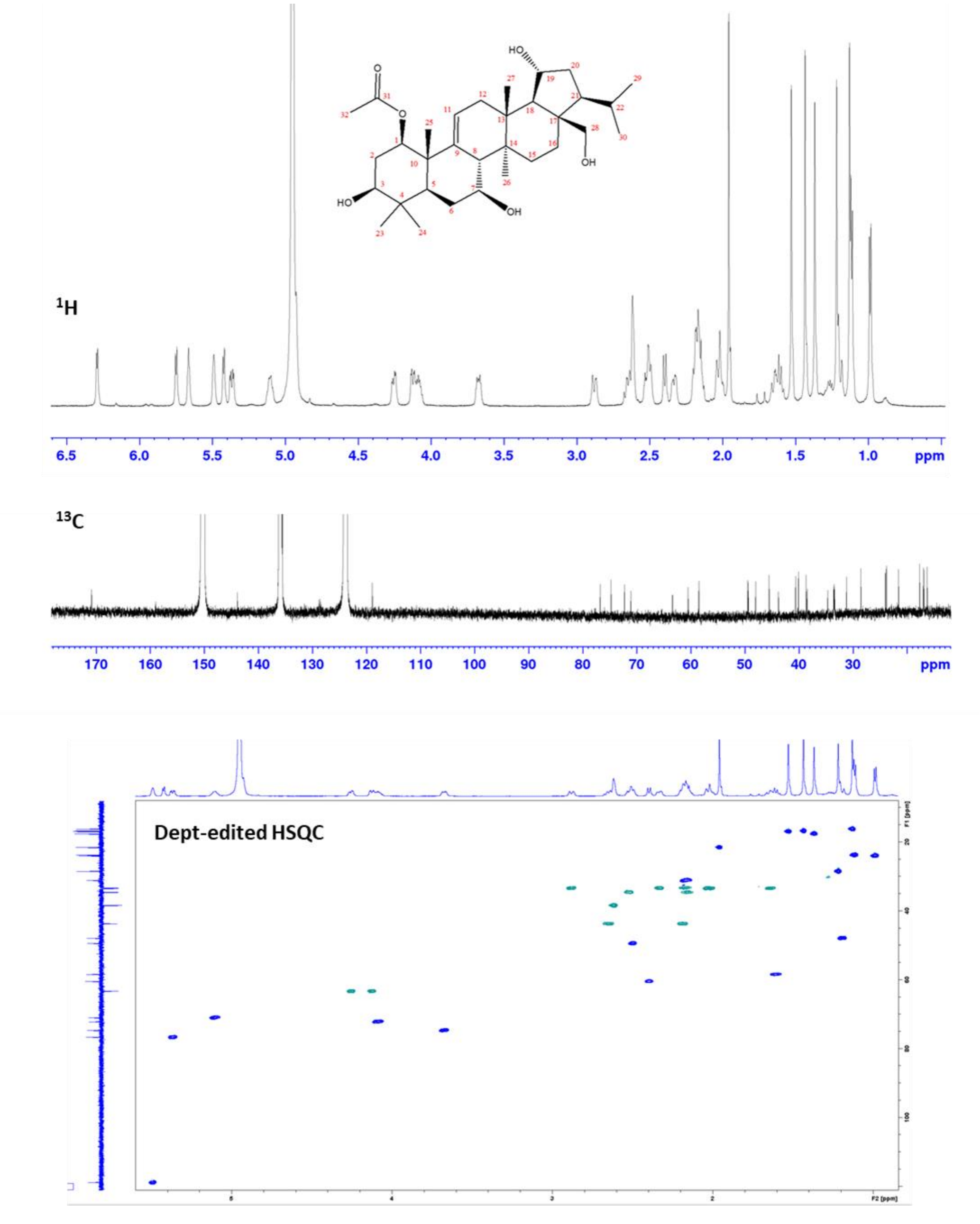

Fig. S31. ^{1}H, ^{13}C, and dept-edited HSQC spectra for brachynacin (**Pyridine-d5**). Referenced to residual solvent peak (^{1}H δ: 8.74) (^{13}C δ: 150.3)]. 600 MHz instrument.

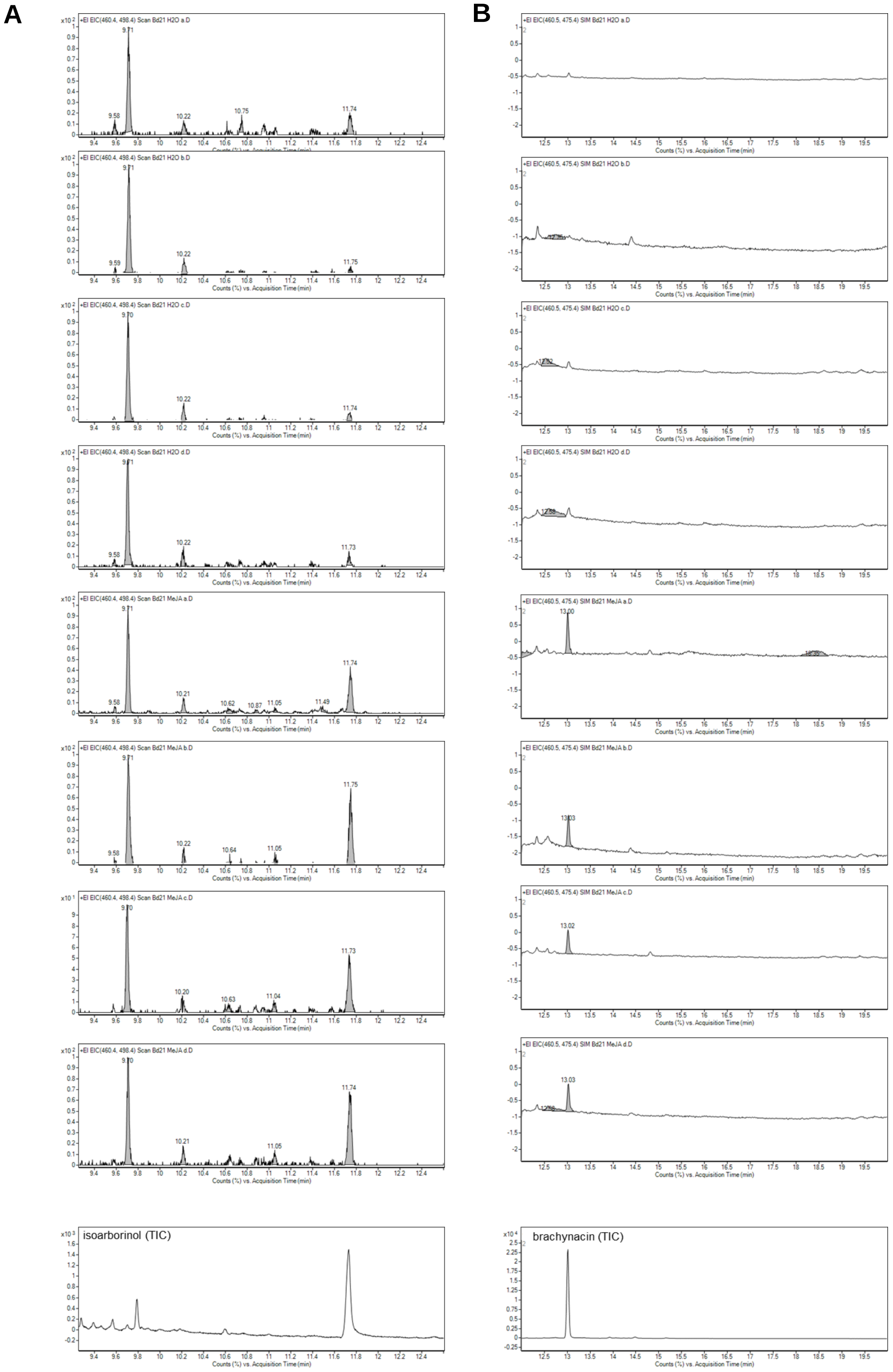

Fig. S32. GC-MS analysis of TMS-derivatized extracts from *B. distachyon* leaves treated with methyl jasmonate (MeJa), or H_2O (control) for 12 hours. Extracted ion chromatograms are for ions representing (A) isoarborinol (498.4, Rt 11.73), 5α-cholestan-3β-ol (460.4, Rt 9.70) and (B) brachynacin (475.4, Rt 13.02). Y-axes are linked to peak of internal standard- 5α-cholestan-3β-ol. Extracts from four biological replicates are shown. *B. distachyon* extracts were compared to isoarborinol and brachynacin purified from *N. benthamiana* for identification.

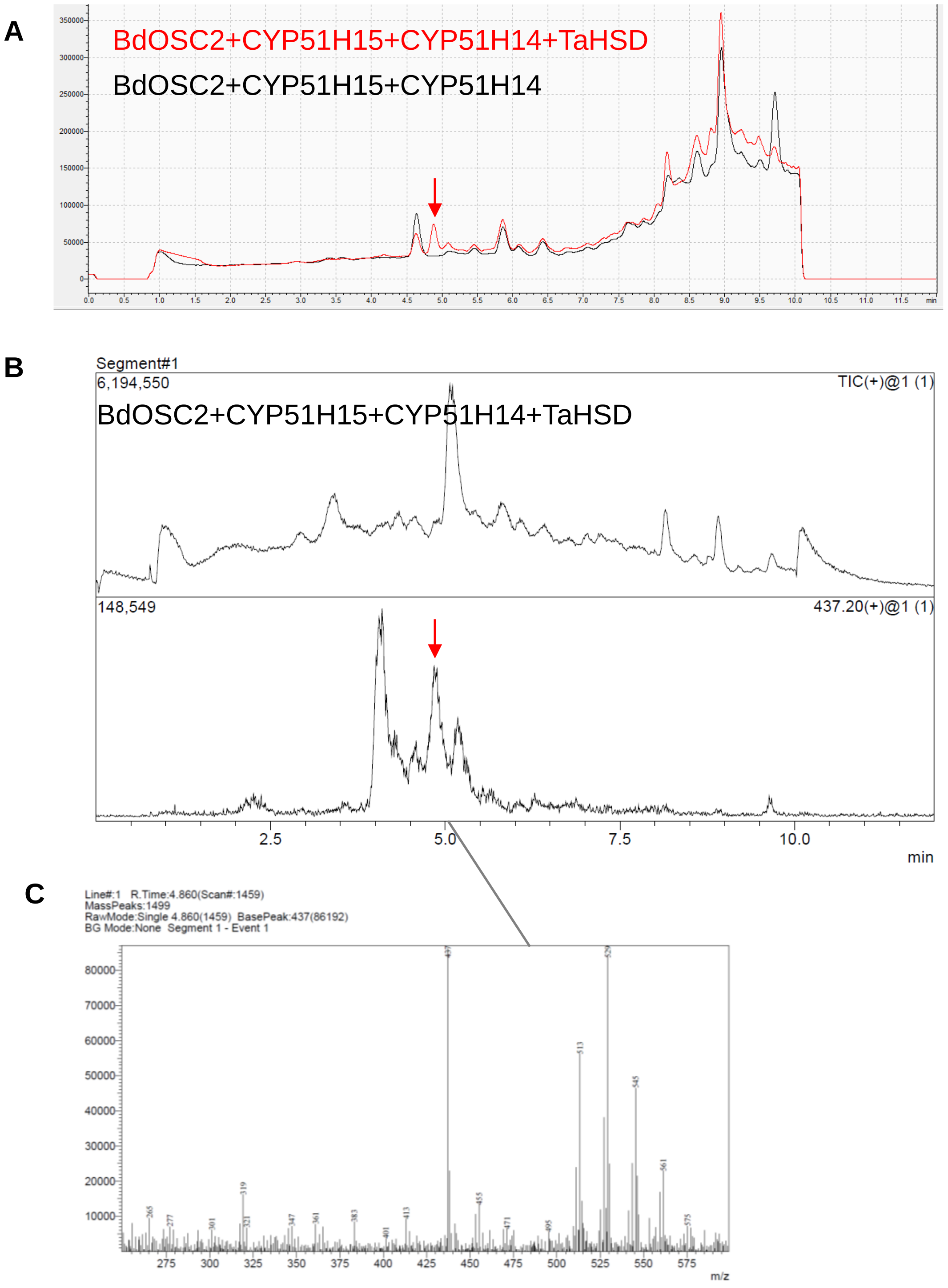

Fig. S33. LC-MS detection of 7,9,28-trihydroxy-isoarborinone molecular ion. (A) CAD chromatogram. (B) MS chromatogram- TIC and EIC of 437.2. (C) mass spectra at peak retention time.

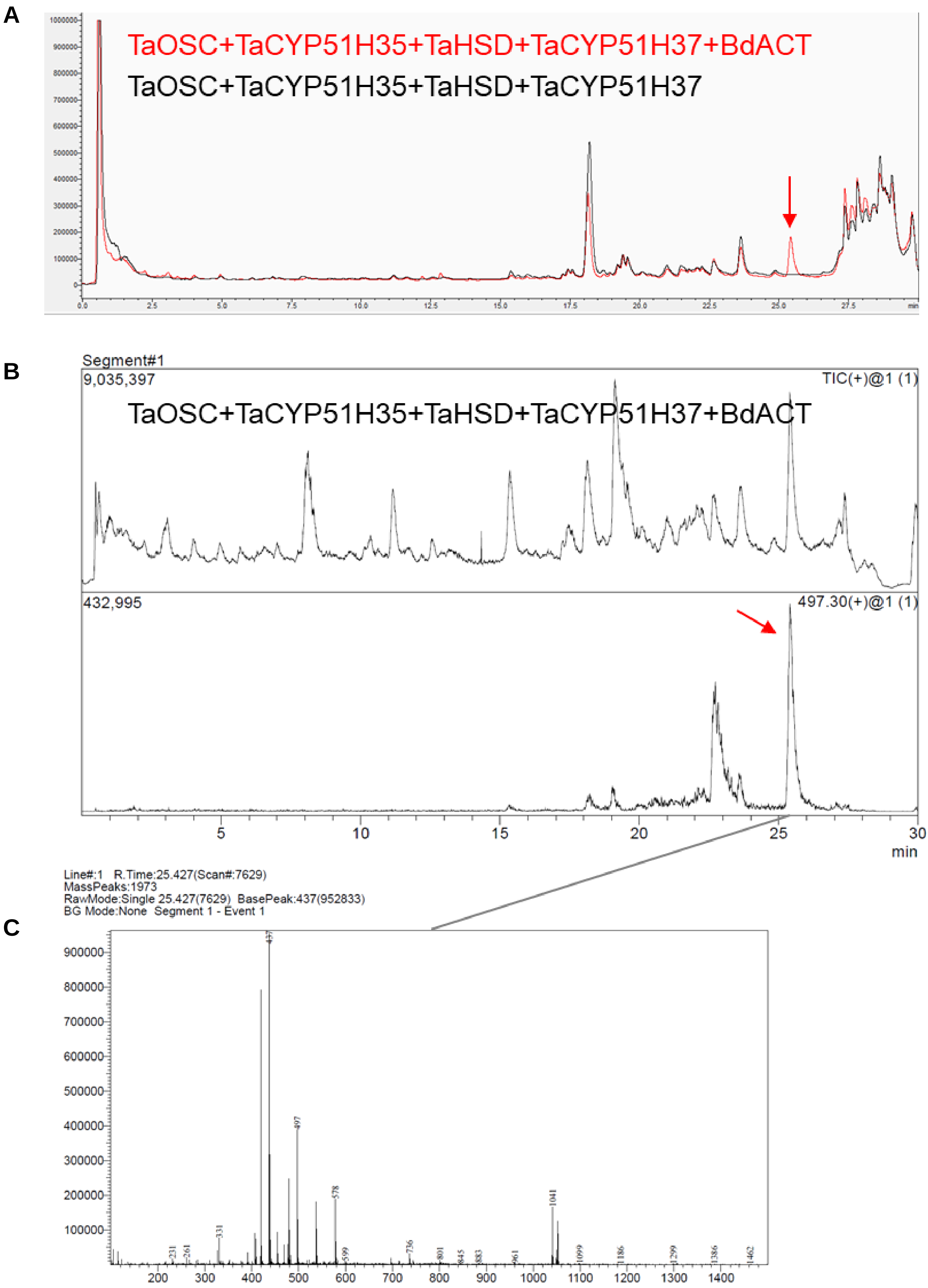

Fig. S34. LC-MS detection of acetyl-ellarinacin molecular ion. (A) CAD chromatogram. (B) MS chromatogram- TIC and EIC of 497.3. (C) mass spectra at peak retention time.

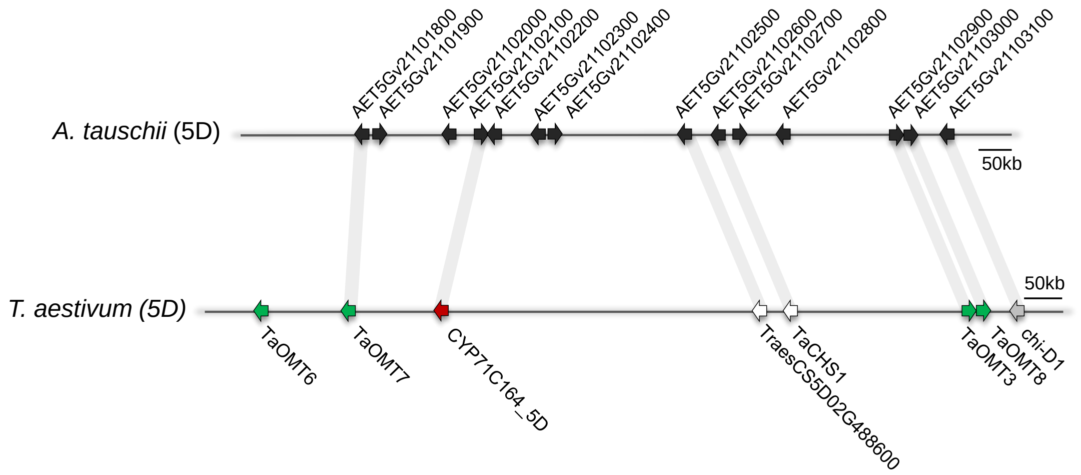

Fig. S35. Wheat flavonoid producing cluster, BGC 4(5D), is conserved in the *Aegilops tauschii* genome (Aet v4.0). Grey lines denote orthologous genes, as assigned by Ensembl Plants (http://www.plants.ensembl.org). Genomic location of the *TaOMT6 A. tauschii* ortholog, *AET0Gv20112500*, is unassigned.

A

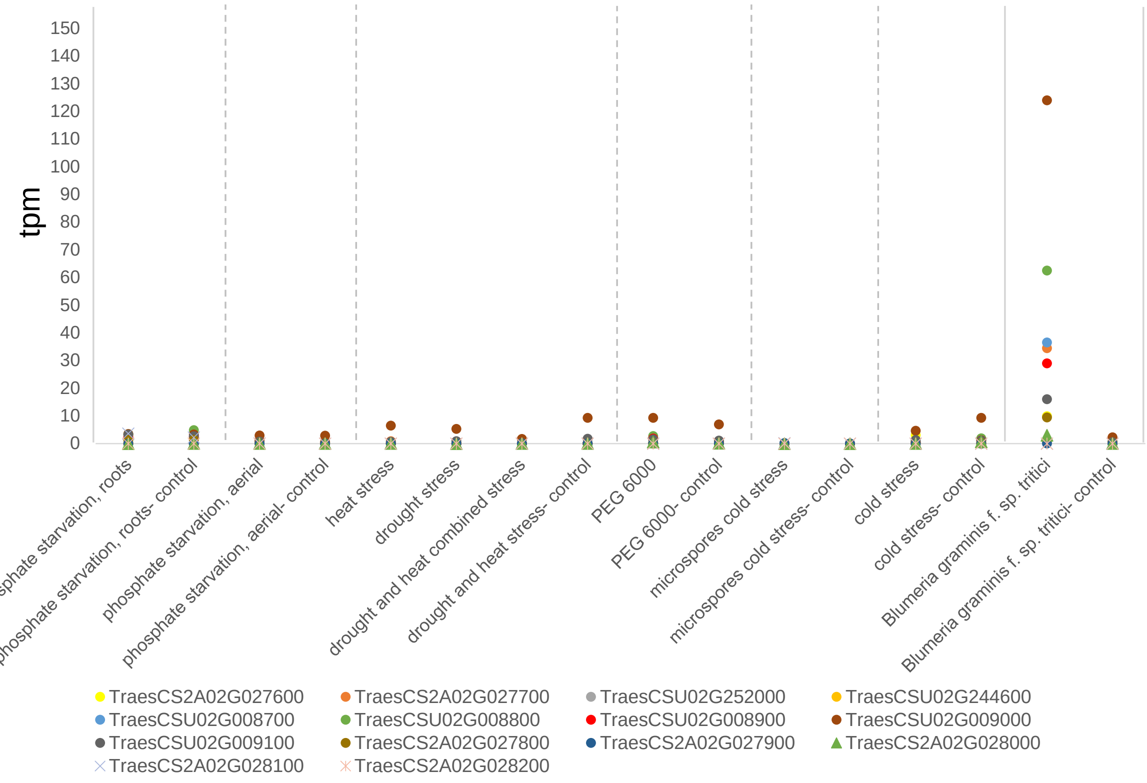

B

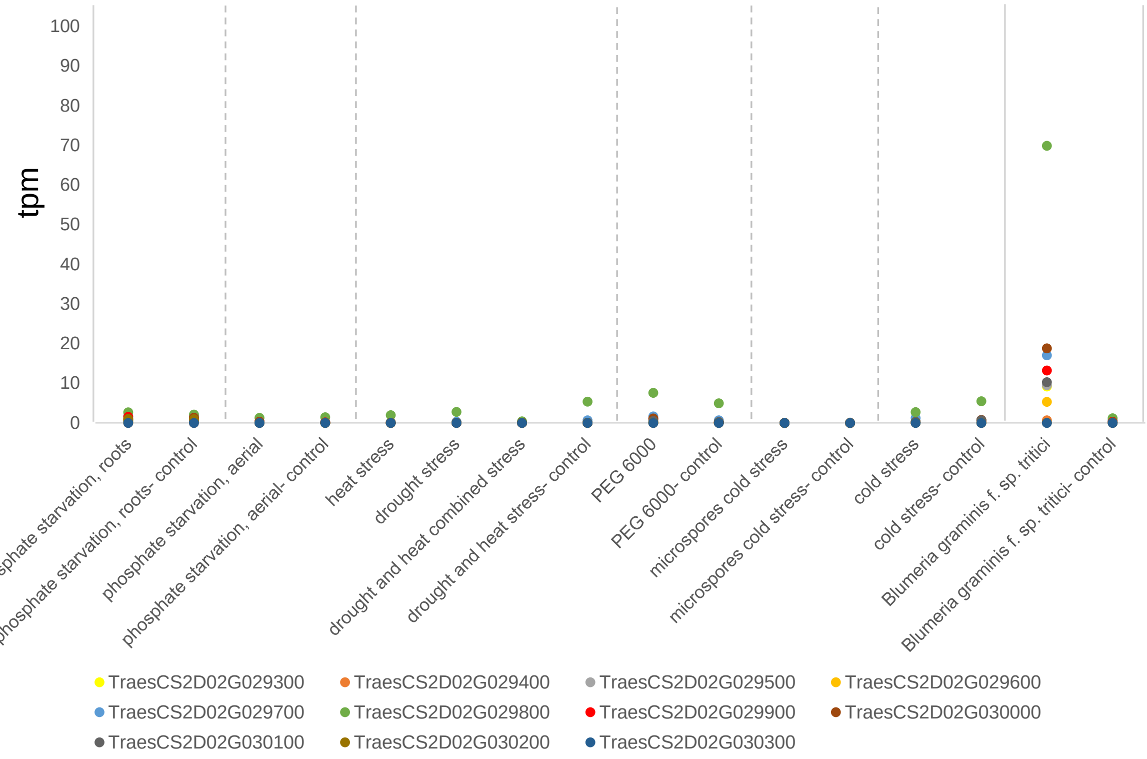

Fig. S36. Expression of wheat diterpene BGCs 1(2A/2D) under abiotic stress. (A) Genes in Chr.2A cluster. (B) Genes in Chr.2D cluster. Expression data extracted from http://www.wheat-expression.com includes studies applying phosphate starvation (41), heat and drought stress (42), PEG 6000, microspores cold stress (43) and cold stress (44). Data of powdery mildew infection experiment (*Blumeria graminis f. sp. tritici*) (39) is shown for comparison. tpm, transcripts per million. See Table S1 for functional annotations of gene IDs.

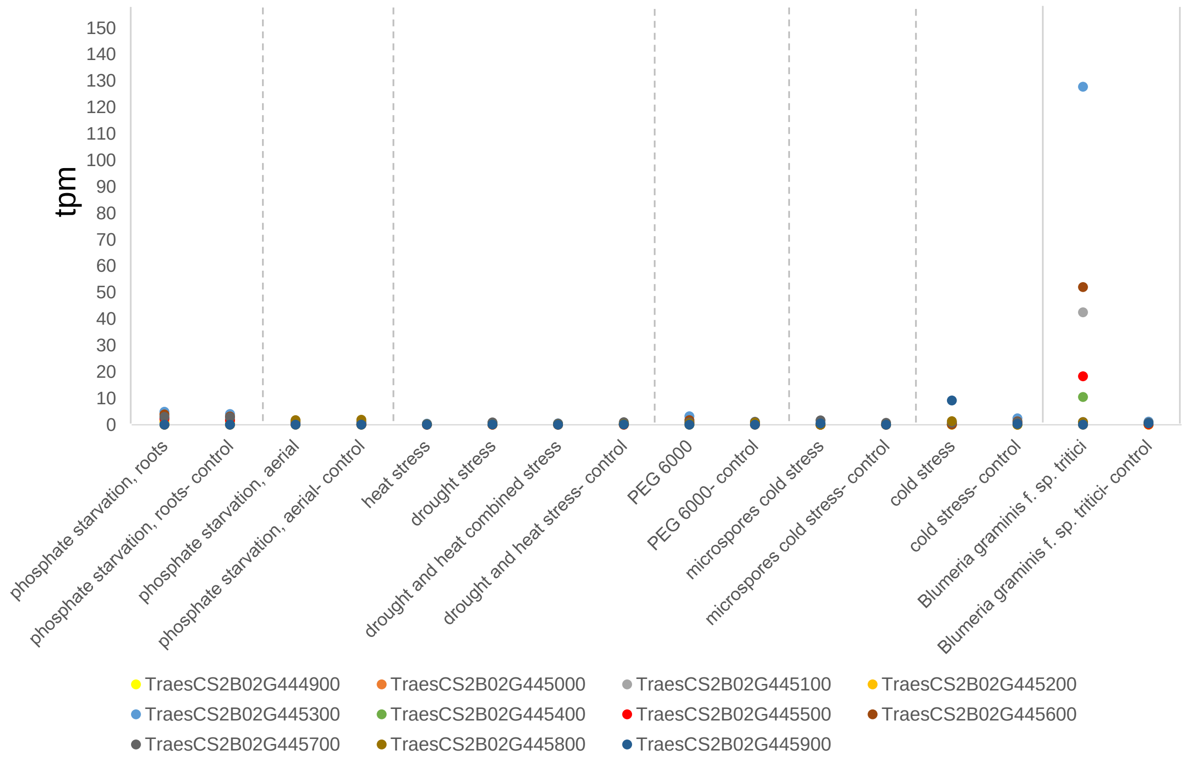

Fig. S37. Expression of wheat diterpene BGC 2(2B) under abiotic stress. Expression data extracted from http://www.wheat-expression.com includes studies applying phosphate starvation (41), heat and drought stress (42), PEG 6000, microspores cold stress (43) and cold stress (44). Data of powdery mildew infection experiment (*Blumeria graminis f. sp. tritici*) (39) is shown for comparison. tpm, transcripts per million. See Table S1 for functional annotations of gene IDs.

SI Tables

Table S1. Genes comprising six wheat BGCs, and the co-expression network modules to which they belong. In bold: genes that are co-expressed (r-val>0.7) with bait genes (asterisked)

BGC no.	Gene ID	Gene annotation	Gene name in this study	Co-expression network module
**1(2A)**	TraesCS2A02G027600	Kaurene synthase		8
	TraesCS2A02G027700	Cytochrome P450		8
	TraesCSU02G252000	Copalyl diphosphate synthase		34
	TraesCSU02G244600	Copalyl diphosphate synthase		34
	TraesCSU02G244600	Copalyl diphosphate synthase		34
	**TraesCSU02G008700**	**Kaurene synthase***	**TaKSL1**	**25**
	**TraesCSU02G008800**	**Cytochrome P450**		**25**
	**TraesCSU02G008900**	**Cytochrome P450**		**25**
	**TraesCSU02G009000**	**Glycosyltransferase**		**12**
	**TraesCSU02G009100**	**Cytochrome P450**		**25**
	**TraesCS2A02G027800**	**Copalyl diphosphate synthase**	**TaCPS2**	**25**
	TraesCS2A02G027900	RING/FYVE/PHD zinc finger superfamily protein		N/A
	**TraesCS2A02G028000**	**Glycosyltransferase**		**25**
	TraesCS2A02G028100	Kaurene synthase		N/A
	TraesCS2A02G028200	Copalyl diphosphate synthase		N/A
**1(2D)**	TraesCS2D02G029300	Cytochrome P450		8
	TraesCS2D02G029400	Kaurene synthase		34
	TraesCS2D02G029500	Cytochrome P450		34
	**TraesCS2D02G029600**	**Copalyl diphosphate synthase**	**TaCPS-D2**	**25**
	**TraesCS2D02G029700**	**Cytochrome P450**		**12**
	**TraesCS2D02G029800**	**Glycosyltransferase**		**25**
	**TraesCS2D02G029900**	**Cytochrome P450**		**25**
	**TraesCS2D02G030000**	**Cytochrome P450**		**25**
	**TraesCS2D02G030100**	**Kaurene synthase***	**TaKSL-D1**	**25**
	TraesCS2D02G030200	Kaurene synthase	TaKSL4	N/A
	TraesCS2D02G030300	Copalyl diphosphate synthase		N/A
**2(2B)**	TraesCS2B02G444900	Kaurene synthase		N/A
	TraesCS2B02G445000	Cytochrome P450		N/A
	**TraesCS2B02G445100**	**Kaurene synthase**	**TaKSL3**	**25**
	**TraesCS2B02G445200**	**Kaurene synthase***	**TaKSL2**	**25**
	**TraesCS2B02G445300**	**Cytochrome P450**		**25**
	TraesCS2B02G445400	Cytochrome P450		0
	**TraesCS2B02G445500**	**Ent-copalyl diphosphate synthase**	**TaCPS1**	**25**
	**TraesCS2B02G445600**	**Cytochrome P450**		**25**
	TraesCS2B02G445700	Ent-kaurene synthase		0
	TraesCS2B02G445800	Kaurene synthase		N/A
	TraesCS2B02G445900	Kaurene synthase		N/A
**3(5A)**	**TraesCS5A02G004500**	**Cytochrome P450-like protein**	**TaCYP51H35_5A**	**34**
	**TraesCS5A02G004600**	**Cytochrome P450-like protein**	**TaCYP51H13_5A**	**25**
	**TraesCS5A02G004700**	**Cytochrome P450-like protein**	**TaCYP51H37_5A**	**25**
	**TraesCS5A02G004800**	**Hydroxysteroid dehydrogenase, putative**	**TaHSD_5A**	**25**
	**TraesCS5A02G004900**	**Terpene cyclase/mutase family member***	**TaOSC_5A**	**25**
**3(5D)**	**TraesCS5D02G011800**	**Terpene cyclase/mutase family member***	**TaOSC (TaIAS)**	**25**
	**TraesCS5D02G011900**	**Hydroxysteroid dehydrogenase, putative**	**TaHSD (TaHID)**	**25**
	**TraesCS5D02G012000**	**Cytochrome P450-like protein**	**TaCYP51H37_5D (TaHIO)**	**25**
	**TraesCS5D02G012100**	**Cytochrome P450-like protein**	**TaCYP51H13P_5D**	**25**
	**TraesCS5D02G012200**	**Obtusifoliol 14-alpha demethylase**	**TaCYP51H13P_5D**	**25**
	**TraesCS5D02G012300**	**Cytochrome P450-like protein**	**TaCYP51H35_5D (TaIAH)**	**34**
**4(5D)**	**TraesCS5D02G488300**	**O-methyltransferase-like protein**	**TaOMT6**	**25**
	**TraesCS5D02G488400**	**O-methyltransferase-like protein**	**TaOMT7**	**25**
	**TraesCS5D02G488500**	**Cytochrome P450**	**TaCYP71C164_5D**	**25**
	TraesCS5D02G488600	Chalcone synthase		0
	**TraesCS5D02G488700**	**Chalcone synthase***	**TaCHS1**	**25**
	**TraesCS5D02G488800**	**O-methyltransferase**	**TaOMT3**	**25**
	**TraesCS5D02G488900**	**O-methyltransferase**	**TaOMT8**	**25**
	TraesCS5D02G489000	Chalcone-flavanone isomerase family protein	chi-D1	10

Table S2. Genomic location of benzoxazinoid biosynthetic genes in the wheat genome

Chromosomal arm location	Gene name	GenBank accession	Gene ID	Gene annotation	Genomic position
2BL	Taglu1b	AB236422*	TraesCS2B02G599800	Beta-glucosidase	Chr.2B: 782,533,511-782,538,118
	Taglu1a	AB100035*	TraesCS2B02G600200	Beta-glucosidase	Chr.2B: 783,226,574-783,231,181
2DL	Taglu1c	AB236423*	TraesCS2D02G594400	Beta-glucosidase	Chr.2D: 648,238,204-648,242,750
4AS	TaBx1A	AB094060†	TraesCS4A02G097400	Tryptophan synthase alpha chain	Chr.4A: 108,369,445-108,371,437
	TaBx2A	AB042630†	TraesCS4A02G097500	Cytochrome P450	Chr.4A: 108,373,749-108,375,763
4BL	TaBx2B	AB042631†	TraesCS4B02G207000	Cytochrome P450	Chr.4B: 440,921,340-440,923,458
	TaBx1B	AB124849†	TraesCS4B02G207100	Tryptophan synthase alpha chain	Chr.4B: 440,925,239-440,927,352
4DL	TaBx2D	AB124851†	TraesCS4D02G207800	Cytochrome P450	Chr.4D: 357,525,599-357,527,592
	TaBx1D	AB124850†	TraesCS4D02G207900	Tryptophan synthase alpha chain	Chr.4D: 357,529,483-357,531,474
5AS	TaBx5A	AB042629†	TraesCS5A02G008700	Cytochrome P450	Chr.5A: 6,287,359-6,290,472
	TaBx4A	AB124854†	TraesCS5A02G008800	Cytochrome P450	Chr.5A: 6,411,078-6,413,189
	TaBx3A	AB042628†	TraesCS5A02G008900	Cytochrome P450	Chr.5A: 6,422,019-6,424,272
5BS	TaBx5B	AB124856†	TraesCS5B02G007000	Cytochrome P450	Chr.5B: 8,185,879-8,188,188
	TaBx4B	AB124855†	TraesCS5B02G007100	Cytochrome P450	Chr.5B: 8,342,582-8,344,606
	TaBx3B	AB124853†	TraesCS5B02G007200	Cytochrome P450	Chr.5B: 8,351,751-8,353,860
5DS	TaBx5D	AB124857†	TraesCS5D02G014100	Cytochrome P450	Chr.5D: 7,964,211-7,966,925
	TaBx4D	AB042627†	TraesCS5D02G014200	Cytochrome P450	Chr.5D: 8,020,016-8,022,015
	TaBx3D	AB124852†	TraesCS5D02G014300	Cytochrome P450	Chr.5D: 8,033,202-8,035,560
7BS	TaGTb	AB547238*	TraesCS7B02G016800	UDP-glycosyltransferase	Chr.7B: 14,726,749-14,728,414
7DL	TaGTd	AB547240*	TraesCS7D02G116700	UDP-glycosyltransferase	Chr.7D: 71,837,050-71,839,162
Unassigned	Taglu1d	AB548284*	TraesCSU02G036600	Beta-glucosidase, putative	Chr.Un: 31,204,475-31,209,405
	TaGTc	AB547239*	TraesCSU02G093300	UDP-glycosyltransferase	Chr.Un: 82,568,510-82,570,472
	TaGTa	AB547237*	TraesCSU02G095600	UDP-glycosyltransferase	Chr.Un: 84,308,887-84,310,663

*Reference for GenBank accession number: (16)
†Reference for GenBank accession number: (45)

Table S3. Gene expression values of 3-5A and 3-5D cluster paralogs

Gene accession	Annotation	Average expression (tpm)	Max expression (tpm)
TraesCS5D02G006200	Cytochrome P450-like protein	0.112±0.035	8.896
TraesCS5D02G006300	Hydroxysteroid dehydrogenase, putative	0.009±0.003	0.792
TraesCS5D02G006400	Cytochrome P450-like protein	0±0	0.018
TraesCS5D02G006500	Cytochrome P450-like protein	0.018±0.004	0.963
TraesCS5D02G006600	Terpene cyclase/mutase family member	0.009±0.004	1.257
TraesCS5D02G006700	Terpene cyclase/mutase family member	0.011±0.003	0.566
TraesCS5A02G005600	Cytochrome P450-like protein	0.006±0.001	0.186
TraesCS5A02G005900	Terpene cyclase/mutase family member	0.025±0.01	2.936
TraesCS5A02G006000	Cytochrome P450-like protein	0.0075±0.003	0.897
TraesCS5B01G004500	Cytochrome P450-like protein	0.148±0.031	5.928
TraesCS5B01G004800	Terpene cyclase/mutase family member	0.005±0.001	0.213
TraesCS5B01G004900	Cytochrome P450-like protein	0.669±0.2334	61.012
TraesCS5B01G004600	Cytochrome P450-like protein	0.250±0.0779	24.462

Average and maximal normalized gene expression values of TaOSC, TaHSD and TaCYP51H35/37 paralogs, across 379 datapoints in http://www.wheat-expression.com transcriptomes dataset. tpm, transcripts per million.

Table S4. ^{13}C δ of **19-hydroxy-isoarborinol** in this work [100 MHz] compared to the literature (**rubiarbonol K**) [125 MHz]. P**yridine-d5** [referenced to the most downfield peak reported in the literature] (33).

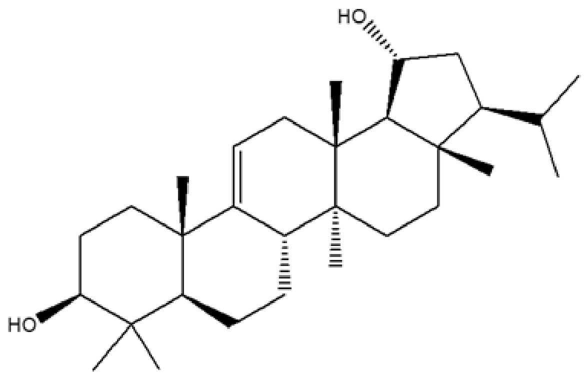

^{13}C δ This work	^{13}C δ Lit	Δ (^{13}C δ This work - Lit)
148.8	148.8	0.0
114.9	114.9	0.0
78.1	78.1	0.0
70.1	70.2	-0.1
59.0	59.0	0.0
57.7	57.8	-0.1
52.9	52.9	0.0
44.0	44.1	-0.1
41.9	42.0	-0.1
41.1	41.1	0.0
39.9	40.0	-0.1
39.7	39.8	-0.1
38.4	38.5	-0.1
37.6	37.6	0.0
37.2	37.2	0.0
36.6	36.6	0.0
36.5	36.6	-0.1
30.7	30.8	-0.1
29.9	29.9	0.0
28.9	29.0	-0.1
28.7	28.7	0.0
27.1	27.2	-0.1
23.2	23.2	0.0
22.4	22.5	-0.1
22.2	22.3	-0.1
21.9	21.9	0.0
17.5	17.6	-0.1
16.8	16.8	0.0
16.6	16.6	0.0
15.9	15.9	0.0

Table S5. ^{13}C & ^{1}H δ assignments for **ellarinacin**. **Pyridine-d5** [referenced to residual solvent peak (^{1}H δ: 8.74) (^{13}C δ: 150.3)]. Assignments were made via a combination of ^{1}H, ^{13}C, DEPT-edited HSQC, HMBC, COSY and 2D NOESY experiments. Where signals overlap ^{1}H δ is reported as the centre of the respective HSQC crosspeak. C3-C2 epoxide was assigned as beta due to an NOE observed between C2-H and C5-H.

Carbon numbering scheme and selected COSY, HMBC and NOESY

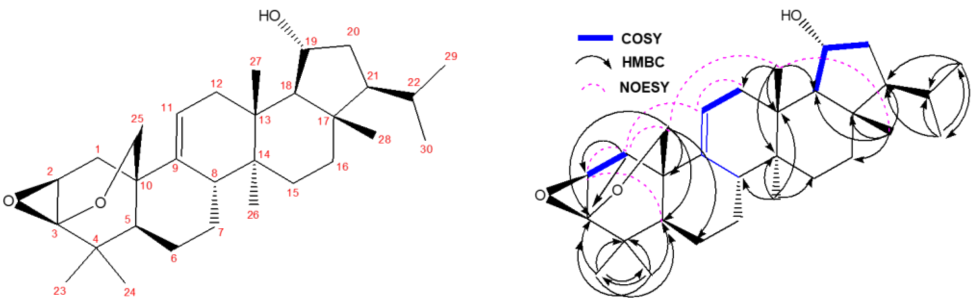

Carbon #	^{13}C δ (100 MHz)	^{1}H δ (400 MHz)	Carbon #	^{13}C δ (100 MHz)	^{1}H δ (400 MHz)
**9**	139.69	/	**13**	38.37	/
**11**	123.34	5.69 (1H, m)	**12**	38.34	2.47 (2H, m)
**3**	98.76	/	**16**	37.50	1.67 (2H, m)
**25**	72.56	4.51 (1H, dd *J* = 8.5, 3.1)<br>3.65 (1H, dd *J* = 8.5, 1.0)	**1**	37.08	2.33 (1H, m)<br>2.21 (1H, m)
**2**	72.21	3.92 (1H, td *J* = 10.7, 3.1)	**15**	32.11	2.98 (1H, m)<br>1.86 (1H, m)
**19**	70.69	4.50 (1H, m)	**6**	31.83	2.31 (1H, m)<br>1.50 (1H, m)
**18**	59.45	2.04 (1H, m)	**22**	31.13	1.41 (1H, m)
**21**	58.21	1.39 (1H, m)	**7**	30.87	2.40 (1H, m)<br>2.18 (1H, m)
**8**	50.62	2.22 (1H, m)	**24**	28.67	1.26 (3H, s)
**5**	48.05	1.63 (1H, m)	**29**	23.61	0.86 (3H, d *J* = 5.9)
**17**	44.15	/	**30**	22.57	0.90 (3H, d *J* = 5.9)
**20**	42.31	2.07 (1H, m)<br>1.97 (1H, m)	**23**	19.93	1.40 (3H, s)
**4**	40.44	/	**27**	17.21	1.10 (3H, s)
**14**	40.29	/	**26**	16.45	1.31 (3H, s)
**10**	39.00	/	**28**	16.39	0.90 (3H, s)

Table S6. Pairwise alignment of putative proteins from orthologous biosynthetic gene clusters in common wheat, *Aegilops tauschii*, and wild emmer wheat.

T. aestivum **(Chr.5D)**	***A. tauschii***	**% identity**
TraesCS5D02G011800<br>TaOSC_5D	AET5Gv20013100	99.9
TraesCS5D02G011900<br>TaHSD_5D	AET5Gv20013000	100
TraesCS5D02G012000<br>TaCYP51H37_5D	AET5Gv20012900	100
TraesCS5D02G012100/<br>TraesCS5D02G012200<br>TaCYP51H13P_5D	AET5Gv20012800	99.4
TraesCS5D02G012300<br>TaCYP51H35_5D	AET5Gv20012700	99.6

T. aestivum **(Chr.5A)**	***T. turgidum ssp. diccocoides***	**% identity**
TraesCS5A02G004500<br>TaCYP51H35_5A	TRIDC5AG000720	99.6
TraesCS5A02G004600<br>TaCYP51H13_5A	TRIDC5AG000730	99.4
TraesCS5A02G004700<br>TaCYP51H37_5A	TRIDC5AG000740	100
TraesCS5A02G004800<br>TaHSD_5A	TRIDC5AG000750	99.7
TraesCS5A02G004900<br>TaOSC_5A	TRIDC5AG000760	99.9

Table S7. Normalized expression values (RPKM) of *AsOSC1* and *AsCYP51H73* in an RNA-seq dataset sampling six *Avena strigosa* tissues (28). Average and maximum RPKM values of entire dataset are shown.

	**Leaf**	**Root tip**	**Panicle**	**Root**	**Shoot**	**Spikelet**
AsOSC1	0.00	0.00	0.30	0.00	0.00	0.01
AsCYP51H73	0.00	0.00	0.00	0.00	0.00	0.00
Avg. RPKM	25.94	22.46	20.48	23.02	30.79	19.40
Max RPKM	358635.00	452433.07	76143.40	316116.93	530585.92	30338.72

Table S8. ^{13}C & ^{1}H δ assignments for **brachynacin**. **Pyridine-d5** [referenced to residual solvent peak (^{1}H δ: 8.74) (^{13}C δ: 150.3)]. Assignments were made via a combination of ^{1}H, ^{13}C, DEPT-edited HSQC, HMBC, COSY, TOCSY and 2D ROESY experiments. Where signals overlap ^{1}H δ is reported as the centre of the respective HSQC crosspeak. C1-OAc was assigned as beta due to NOEs observed between C1-H and C3-H, and C5-H. C7-OH was assigned as beta due to NOEs observed between C7-H and C26-H_3 and C5-H.

Carbon numbering scheme and selected COSY, HMBC and TOCSY

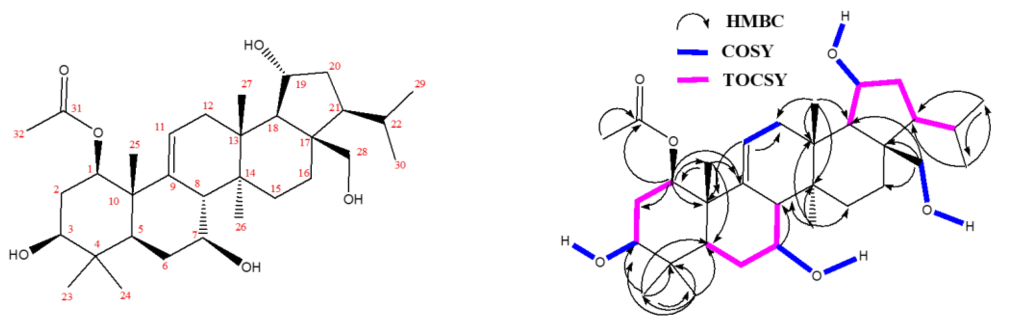

Carbon #	^{13}C δ (150 MHz)	^{1}H δ (600 MHz)	Carbon #	^{13}C δ (150 MHz)	^{1}H δ (600 MHz)
**31**	170.84	/	**4**	40.09	/
**9**	143.87	/	**13**	38.66	/
**11**	118.92	5.49 (1H, m)	**12**	38.47	2.62 (2H, m)
**1**	76.75	5.37 (1H, dd *J* = 11.5, 4.3)	**2**	34.67	2.52 (1H, m)<br>2.16 (1H, m)
**3**	74.75	3.67 (1H, m)	**6**	33.56	2.33 (1H, m)<br>2.18 (1H, m)
**7**	72.26	4.09 (1H, m)	**15**	33.50	2.03 (1H, m)<br>1.64 (1H, m)
**19**	71.07	5.11 (1H, m)	**16**	33.43	2.88 (1H, m)<br>2.03 (1H, m)
**28**	63.38	4.26 (1H, dd *J* = 11.1, 4.2)<br>4.12 (1H, dd *J* = 11.1, 4.2)	**22**	31.18	2.17 (1H, m)
**18**	60.50	2.40 (1H, d *J* = 9.9)	**23**	28.56	1.22 (3H, s)
**21**	58.50	1.61 (1H, m)	**30**	24.03	0.99 (3H, d *J* = 6.5)
**8**	49.47	2.50 (1H, m)	**29**	23.81	1.11 (3H, d *J* = 6.5)
**17**	49.38	/	**32**	21.59	1.96 (3H, s)
**5**	48.00	1.20 (1H, m)	**26**	17.67	1.34 (3H, s)
**10**	45.51	/	**27**	16.99	1.53 (3H, s)
**20**	43.80	2.65 (1H, m)<br>2.19 (1H, m)	**25**	16.84	1.43 (3H, s)
**14**	40.62	/	**24**	16.29	1.13 (3H, s)

Exchangable Protons (Assigned by COSY)

C3-OH: δ 6.19 (1H, d, *J* = 5.2); **C7-OH:** δ 5.75 (1H, d, *J* = 6.2)**;** **C28-OH:** δ 5.66 (1H, brt, *J* = 3.9), **C19-OH:** δ 5.42 (1H, d, J= 5.8)**.**

Table S9. *B. distachyon* putative terpene cluster homologous to wheat BGC 2(2B)

Gene ID	Location	Description
Bradi5g21383	Bd5:24179552..24182576 (+ strand)	farnesyl diphosphatase/FPP phosphatase
Bradi5g21387	Bd5:24182863..24188013 (+ strand)	ent-kaurene synthase
Bradi5g21400	Bd5:24188931..24190869 (- strand)	cytochrome P450
Bradi5g21410	Bd5:24194057..24196081 (- strand)	cytochrome P450
Bradi5g21420	Bd5:24200947..24203100 (- strand)	germacrene A alcohol dehydrogenase
Bradi5g21430	Bd5:24205464..24207731 (- strand)	germacrene A alcohol dehydrogenase
Bradi5g21440	Bd5:24210985..24218157 (+ strand)	ent-kaurene synthase
Bradi5g21447	Bd5:24218971..24221276 (- strand)	cytochrome P450
Bradi5g21460	Bd5:24222480..24225443 (- strand)	cytochrome P450
Bradi5g21465	Bd5:24227072..24227779 (+ strand)	unknown protein
Bradi5g21470	Bd5:24229095..24230866 (- strand)	cytochrome P450
Bradi5g21480	Bd5:24238153..24242304 (+ strand)	ent-kaurene synthase
Bradi5g21488	Bd5:24244530..24246038 (+ strand)	cytochrome P450
Bradi5g21492	Bd5:24246237..24247028 (+ strand)	unknown protein
Bradi5g21497	Bd5:24270996..24274843 (+ strand)	ent-kaurene synthase

Table S10. Oligonucleotides used in this study

**Full CDS cloning**	
TaCYP51H35_5D F	GCGCCGTCTCGCTCGAATGGACTTAGCAAGTCTC
TaCYP51H35_5D R	GCGCCGTCTCGCTCGAAGCCTACAAAATGCCATTCCT
TaCYP51H37_5D F	GCGCCGTCTCGCTCGAATGGAGATGGCAAGTAGCGC
TaCYP51H37_5D R	GCGCCGTCTCGCTCGAAGCCTAGCCTAGCAGCTGGCGCCTCTTGTAG
TaCYP51H13_5A F	GCGCCGTCTCGCTCGAATGGACTTGACAAGTCTCACTACG
TaCYP51H13_5A R	GCGCCGTCTCGCTCGAAGCTTAAATTCCATTTCTCGTATATCTCA
A. tauschii IAH F	GGGGACAAGTTTGTACAAAAAAGCAGGCTATGGACTTAGCAAGTCTCAC
A. tauschii IAH R	GGGGACCACTTTGTACAAGAAAGCTGGGTCTAATTAAGTGACTGCAAAATGC
BdOSC2 F	GCGCCGTCTCGCTCGAATGTGGAAGCTAAAGATCGCA
BdOSC2 R	GCGCCGTCTCGCTCGAAGCTTATGCCTTTTGTGCTTGCAG
BdCYP51H14 F	GCGCCGTCTCGCTCGAATGTTCATGACAAGTAGCGCC
BdCYP51H14 R	GCGCCGTCTCGCTCGAAGCCTAGCCCAACAGCCGATGTC
BdCYP51H15 F	GCGCCGTCTCGCTCGAATGGACTTGGCAAGCACAGC
BdCYP51H15 R	GCGCCGTCTCGCTCGAAGCCTAAGTGCTAGCACGGCAGC
BdCYP51H16 F	GCGCCGTCTCGCTCGAATGGAATTTACAAGTGGCGAC
BdCYP51H16 R	GCGCCGTCTCGCTCGAAGCCTAGGCTGACATCCTCGATC
TaCYP71C164_5D F	GGGGACAAGTTTGTACAAAAAAGCAGGCTATGGAAGATCTCGTGAAGAAACC
TaCYP71C164_5D R	GGGGACCACTTTGTACAAGAAAGCTGGGTCTACATCCAGGATTTTGGAATTAACAATAG
TaCYP71F53_5D F	GGGGACAAGTTTGTACAAAAAAGCAGGCTATGGAGGGTTGGTTAACCTTATGTTTC
TaCYP71F53_5D R	GGGGACCACTTTGTACAAGAAAGCTGGGTCTATATAGTGGAGCGTACATATGGAATAGC
TaOMT6 F	GGGGACAAGTTTGTACAAAAAAGCAGGCTATGGCGCCCACCCAAGGCAAGCAGAGTTCTCA
TaOMT6 R	GGGGACCACTTTGTACAAGAAAGCTGGGTTCAAGGGTAGAGCTCAATAACAGATCTAACTC
TaOMT3 F	GGGGACAAGTTTGTACAAAAAAGCAGGCTATGGGCTCCACTGCCGTGGAGAAGGTC
TaOMT3 R	GGGGACCACTTTGTACAAGAAAGCTGGGTCTATTTGACGAACTCAATGACCCATGC
TaOMT8 F	GGGGACAAGTTTGTACAAAAAAGCAGGCTATGGGTTCTATCTCCGACGACGAGGCG
TaOMT8 R	GGGGACCACTTTGTACAAGAAAGCTGGGTCTATTTGTTCAACTCAATGACCCATATG
***T. aestivum* RT-PCR**	
TaCYP51H35_5D F	GGTGGGCTGTGGCTCTT
TaCYP51H35_5D R	CTACAAAATGCCATTCCTCTT
TaCYP51H13_5A F	ACCAACATTGATCCACAGC
TaCYP51H13_5A R	TTAAATTCCATTTCTCGTATATCTC
TaCYP51H37_5D F	CCGCTGCTCCAGCCAGTGA
TaCYP51H37_5D R	CTAGCCTAGCAGCTGGCGTCTC
TaOSC_5D F	GGGAGTTCGATCCTGCC
TaOSC_5D R	CTATCTCTTCCAAGCGAATGTG
TaOSC_5A F	TGGAACAACATGGGTATCACATA
TaOSC_5A R	CAGGGCAGTCCGCACG
***T. aestivum* qRT-PCR**	
TaOSC_5D qRT-PCR F	GGGACTGCATATCGAGGGAA
TaOSC_5D qRT-PCR R	CCCCAAGCAATCTCAAAGCA
TaHSD qRT-PCR F	GCCTACTTCTCTGTCGACGT
TaHSD qRT-PCR R	GTTGTTGGTATGATCCGCCG
TaCYP51H35_5D qRT-PCR F	TGGAAGACACATTTGCACCG
TaCYP51H35_5D qRT-PCR R	CGAGCTCAAAGTTCCTCAGC
TaCYP51H37_5D qRT-PCR F	GCTGAACCCACCAACAACAA
TaCYP51H37_5D qRT-PCR R	GTCTTGTCCGCACTGTGAAA
TUBB qPCR F	CAAGGAGGTGGACGAGCAGATG
TUBB qPCR R	GACTTGACGTTGTTGGGGATCCA
***B. distachyon* RT-PCR**	
BdOSC1 F	CATCAGAAGGAGATTCGGAGATA
BdOSC1 R	CCATCGTCATTCATCAGTGATAA
BdOSC2 F	CATCAGAAAGAGATGCGGAGATA
BdOSC2 R	CCATCCTCGTTCATCAGAGATAAC
BdCYP51H14 F	ATGCATACTTCAACAAGGATCTAT
BdCYP51H14 R	CCTTGATGCAACTATGGAGTGTA
BdACT F	ACAAGAATCACATGTGCATTCC
BdACT R	GAATCAAGTAGTGCTGGCGT
BdCYP51H15 F	CTCTTCTTCTCATCACTGCTTTAG
BdCYP51H15 R	TTGACTACAATAGGACTCGCTAC
BdCYP51H16 F	ATGGAATTTACAAGTGGCGAC
BdCYP51H16 R	CTGCCAAGGTATTGTAGTCGA
BdGAPDH F	ATGGGCAAGATTAAGATCGGAA
BdGAPDH R	TTACTGAGTCTTGGCCATGT
***B. distachyon* qRT-PCR**	
BdOSC1 F	CAGGGGCTGGTGTTATTCAA
BdOSC1 R	GTAATCTGCAGCCTTTCGGA
BdOSC2 F	CTCCTGAATTGGCTGGTGAG
BdOSC2 R	GCCATCCTCGTTCATCAGAG
BdCYP51H14 F	GCTGCTCTCTGAAATCGTGA
BdCYP51H14 R	GGCCGTCTTTGTACTTGGAA
BdACT F	AATCACATGTGCATTCCGGT
BdACT R	GCACTCTTTATCGTCTCGGC
BdCYP51H15 F	TCGGTCTCCTATTTGCTGGA
BdCYP51H15 R	ACATTGGGTGGCTAAGCAAA
BdCYP51H16 F	CTCCTTGGACTTCTACACGC
BdCYP51H16 R	CACCTTTTGTCCAAGCAAGC
BdGAPDH F	TTGCTCTCCAGAGCGATGAC
BdGAPDH R	CTCCACGACATAATCGGCAC
**Site-directed mutagenesis**	
TaOSC_5D(I581S) F	GTACCCCAAACACAGTCGCTTGGAAGAG
TaOSC_5D(I581S) R	CTCTTCCAAGCGACTGTGTTTGGGGTAC

Table S11. Isolera Prime gradient conditions

Compound	Column	Solvents	Gradient
Ellarinacin	SNAP Ultra 50 gr	A: hexane<br>B: ethyl acetate	0-100% B (10 CV)<br>100-100% B (1 CV)
	KP- sil 25 gr	A: hexane<br>B: ethyl acetate	50-70% B (60 CV)
	SNAP Ultra 10 gr	A: hexane<br>B: ethyl acetate	0-70% B (117 CV)
Brachynacin	SNAP Ultra 50 gr	A: hexane<br>B: ethyl acetate	0-100% B (10 CV)<br>100-100% B (1 CV)
	Sfar silica D 30 gr	A: hexane<br>B: ethyl acetate	10-100% B (43 CV)<br>100-100% B (5 CV)

REFERENCES

1. A. Untergasser *et al.*, Primer3-new capabilities and interfaces. *Nucleic Acids Research* **40** (2012).
2. M. W. Pfaffl, A new mathematical model for relative quantification in real-time RT-PCR. *Nucleic Acids Research* **29** (2001).
3. J. J. Scholtz, B. Visser, Reference gene selection for qPCR gene expression analysis of rust-infected wheat. *Physiological and Molecular Plant Pathology* **81**, 22-25 (2013).
4. R. Makandar, J. S. Essig, M. A. Schapaugh, H. N. Trick, J. Shah, Genetically engineered resistance to Fusarium head blight in wheat by expression of Arabidopsis NPR1. *Mol Plant Microbe Interact* **19**, 123-129 (2006).
5. J. M. Ruijter *et al.*, Amplification efficiency: linking baseline and bias in the analysis of quantitative PCR data. *Nucleic Acids Research* **37** (2009).
6. R. De La Peña, E. S. Sattely, Rerouting plant terpene biosynthesis enables momilactone pathway elucidation. *Nat Chem Biol* **17**, 205-212 (2021).
7. L. Zheng, U. Baumann, J. L. Reymond, An efficient one-step site-directed and site-saturation mutagenesis protocol. *Nucleic Acids Res* **32**, e115 (2004).
8. C. Roberts *et al.* (1997) A Comprehensive Set of Modular Vectors for Advanced Manipulations and Efficient Transformation of Plants. in *pCAMBIA Vector Release Manual Rockefeller Foundation Meeting of the International Program on Rice Biotechnology, September 15-19, Malacca, Malaysia*.
9. A. Sarrion-Perdigones *et al.*, GoldenBraid 2.0: A Comprehensive DNA Assembly Framework for Plant Synthetic Biology. *Plant Physiology* **162**, 1618-1631 (2013).

10. T. Dahan-Meir *et al.*, Efficient in planta gene targeting in tomato using geminiviral replicons and the CRISPR/Cas9 system. *Plant Journal* **95**, 5-16 (2018).
11. F. Sainsbury, E. C. Thuenemann, G. P. Lomonossoff, pEAQ: versatile expression vectors for easy and quick transient expression of heterologous proteins in plants. *Plant Biotechnology Journal* **7**, 682-693 (2009).
12. J. Reed *et al.*, A translational synthetic biology platform for rapid access to gram-scale quantities of novel drug-like molecules. *Metab Eng* **42**, 185-193 (2017).
13. M. J. Stephenson, J. Reed, B. Brouwer, A. Osbourn, Transient Expression in Nicotiana Benthamiana Leaves for Triterpene Production at a Preparative Scale. *Jove-Journal of Visualized Experiments* 10.3791/58169 (2018).
14. R. H. Ramirez-Gonzalez *et al.*, The transcriptional landscape of polyploid wheat. *Science* **361**, 662-+ (2018).
15. P. Shannon *et al.*, Cytoscape: A software environment for integrated models of biomolecular interaction networks. *Genome Research* **13**, 2498-2504 (2003).
16. B. Makowska, B. Bakera, M. Rakoczy-Trojanowska, The genetic background of benzoxazinoid biosynthesis in cereals. *Acta Physiologiae Plantarum* **37** (2015).
17. P. Borrill, R. Ramirez-Gonzalez, C. Uauy, expVIP: a Customizable RNA-seq Data Analysis and Visualization Platform. *Plant Physiology* **170**, 2172-2186 (2016).
18. M. C. Luo *et al.*, Genome sequence of the progenitor of the wheat D genome Aegilops tauschii. *Nature* **551**, 498-+ (2017).
19. R. Avni *et al.*, Wild emmer genome architecture and diversity elucidate wheat evolution and domestication. *Science* **357**, 93-97 (2017).
20. R. Appels *et al.*, Shifting the limits in wheat research and breeding using a fully annotated reference genome. *Science* **361** (2018).
21. H. Tang *et al.*, Unraveling ancient hexaploidy through multiply-aligned angiosperm gene maps. *Genome Res* **18**, 1944-1954 (2008).
22. M. Alonge, A. Shumate, D. Puiu, A. V. Zimin, S. L. Salzberg, Chromosome-Scale Assembly of the Bread Wheat Genome Reveals Thousands of Additional Gene Copies. *Genetics* **216**, 599-608 (2020).

23. Y. Kawahara *et al.*, Improvement of the Oryza sativa Nipponbare reference genome using next generation sequence and optical map data. *Rice (N Y)* **6**, 4 (2013).

24. P. S. Schnable *et al.*, The B73 Maize Genome: Complexity, Diversity, and Dynamics. *Science* **326**, 1112-1115 (2009).

25. S. Beier *et al.*, Construction of a map-based reference genome sequence for barley, Hordeum vulgare L. *Sci Data* **4**, 170044 (2017).

26. J. P. Vogel *et al.*, Genome sequencing and analysis of the model grass Brachypodium distachyon. *Nature* **463**, 763-768 (2010).

27. S. Ouyang *et al.*, The TIGR Rice Genome Annotation Resource: Improvements and new features. *Nucleic Acids Research* **35**, D883-D887 (2007).

28. Y. Li *et al.,* Subtelomeric assembly of a multi-gene pathway for antimicrobial defense compounds in cereals. *Nature Communications* **12** (2021).

29. D. M. Goodstein *et al.*, Phytozome: a comparative platform for green plant genomics. *Nucleic Acids Research* **40**, D1178-D1186 (2012).

30. M. Krzywinski *et al.*, Circos: An information aesthetic for comparative genomics. *Genome Research* **19**, 1639-1645 (2009).

31. J. K. M. Brown, M. S. Wolfe, Structure and evolution of a population of Erysiphe-graminis f sp hordei. *Plant Pathology* **39**, 376-390 (1990).

32. M. Xu, M. L. Hillwig, S. Prisic, R. M. Coates, R. J. Peters, Functional identification of rice syn-copalyl diphosphate synthase and its role in initiating biosynthesis of diterpenoid phytoalexin/allelopathic natural products. *Plant J* **39**, 309-318 (2004).

33. J. T. Fan *et al.*, Biologically active arborinane-type triterpenoids and anthraquinones from Rubia yunnanensis. *J Nat Prod* **74**, 2069-2080 (2011).

34. J. P. Huelsenbeck, F. Ronquist, MRBAYES: Bayesian inference of phylogenetic trees. *Bioinformatics* **17**, 754-755 (2001).

35. A. Rahier, S. Darnet, F. Bouvier, B. Camara, M. Bard, Molecular and enzymatic characterizations of novel bifunctional 3 beta-hydroxysteroid dehydrogenases/C-4 decarboxylases from Arabidopsis thaliana. *Journal of Biological Chemistry* **281**, 27264-27277 (2006).

36. J. Ma *et al.*, Transcriptome and allele specificity associated with a 3BL locus for Fusarium crown rot resistance in bread wheat. *PLoS One* **9**, e113309 (2014).

37. J. J. Powell *et al.*, The Fusarium crown rot pathogen Fusarium pseudograminearum triggers a suite of transcriptional and metabolic changes in bread wheat (Triticum aestivum L.). *Ann Bot* **119**, 853-867 (2017).

38. J. J. Rudd *et al.*, Transcriptome and Metabolite Profiling of the Infection Cycle of Zymoseptoria tritici on Wheat Reveals a Biphasic Interaction with Plant Immunity Involving Differential Pathogen Chromosomal Contributions and a Variation on the Hemibiotrophic Lifestyle Definition. *Plant Physiology* **167**, 1158-+ (2015).

39. H. Zhang *et al.*, Large-scale transcriptome comparison reveals distinct gene activations in wheat responding to stripe rust and powdery mildew. *BMC Genomics* **15**, 898 (2014).

40. A. Dobon, D. C. E. Bunting, L. E. Cabrera-Quio, C. Uauy, D. G. O. Saunders, The host-pathogen interaction between wheat and yellow rust induces temporally coordinated waves of gene expression. *Bmc Genomics* **17** (2016).

41. Y. Oono *et al.*, Characterisation of the wheat (Triticum aestivum L.) transcriptome by de novo assembly for the discovery of phosphate starvation-responsive genes: gene expression in Pi-stressed wheat. *BMC Genomics* **14**, 77 (2013).

42. Z. Liu *et al.*, Temporal transcriptome profiling reveals expression partitioning of homeologous genes contributing to heat and drought acclimation in wheat (Triticum aestivum L.). *BMC Plant Biol* **15**, 152 (2015).

43. F. Seifert, S. Bössow, J. Kumlehn, H. Gnad, S. Scholten, Analysis of wheat microspore embryogenesis induction by transcriptome and small RNA sequencing using the highly responsive cultivar "Svilena". *BMC Plant Biol* **16**, 97 (2016).

44. Q. Li *et al.*, Understanding the biochemical basis of temperature-induced lipid pathway adjustments in plants. *Plant Cell* **27**, 86-103 (2015).

45. T. Nomura, A. Ishihara, R. C. Yanagita, T. R. Endo, H. Iwamura, Three genomes differentially contribute to the biosynthesis of benzoxazinones in hexaploid wheat. *Proceedings of the National Academy of Sciences of the United States of America* **102**, 16490-16495 (2005).
